# Supplementary material for: Immunogenicity and safety of a recombinant gE-Fc fusion protein subunit vaccine for herpes zoster in adults ≥50 years of age: a randomised, active-controlled, non-inferiority trial
Source: Nat Commun. 2025 Aug 15;16:7590. doi: 10.1038/s41467-025-62800-z (PMC12356908; doi:10.1038/s41467-025-62800-z)
Supplement: Supplementary file 1 — Supplementary Information [file 41467_2025_62800_MOESM1_ESM.pdf]

Supplementary Information

Supplementary Method 2

Table 1. The proportion of participants with positive responses to each cytokine in per-protocol set 6

Table 2. The proportion of participants with positive responses to only 1 cytokine or simultaneous to any combination of 2, 3, or 4 cytokines producing CD4/CD8+ T cells 30 days after vaccination in per-protocol set 7

Table 3. The frequencies of cytokine-producing T cells per 10^5 $CD4^+$ T cells in per-protocol set 8

Table 4. The frequencies of cytokine-producing T cells per 10^5 $CD8^+$ T cells in per-protocol set 10

Table 5. The proportion of participants with simultaneous positive responses to ⩾2 cytokines in full-analysis set 12

Table 6. Geometric mean concentrations and seroconversion rates of gE-specific IgG antibody in full-analysis set 13

Table 7. Multivariable Analysis of anti-gE antibody and CD4+/CD8+ T cells expressing cytokines 30 days after vaccination 15

Table 8. List of severe adverse events 17

Table 9. List of the second dose beyond the allowed window 18

Fig.1 gE-specific IgG antibody concentrations according to age 19

Fig.2 Correlation matrices of antibody levels with cell-mediated immunity responses to LZ901 vaccine and HZ/su vaccine 30 days after vaccination. 20

Fig.3 Solicited adverse reactions that occurred within 30 days after each dose 21

Fig.4 Gating strategy 22

Supplementary Method

Inclusion and exclusion criteria

The volunteers had to meet all following inclusion and exclusion criteria to be eligible for the study.

Inclusion criteria

1. Age 50 years or older on the day of enrollment, regardless of gender, and able to provide legal identification.
2. Able to understand the trial procedures, voluntarily agree to participate in thes tudy, and sign the "Informed Consent Form."
3. Female subjects are not pregnant or lactating. Female subjects with childbearing potential should take reliable contraceptive measures, and have no pregnancy and fertility plan within 8 months;
4. Axillary temperature ≤ 37.0°C on the day of enrollment.
5. Able to attend all scheduled follow-up visits and able to comply with protocol requirements.

Exclusion criteria

1. History of herpes zoster within the past 5 years.
2. Previous vaccination with varicella vaccine or zoster vaccine (including use of registered products or participation in clinical trials for varicella or zoster vaccines).
3. Allergy to any component of the study vaccine: Previous allergy to any recombinant vaccine derived from CHO cells (such as recombinant hepatitis B vaccine [CHO cells]), polysorbates, or a history of severe allergic reactions to any vaccination. (Severe allergic reactions: Anaphylactic shock, allergic laryngeal edema, allergic purpura, thrombocytopenic purpura, localized allergic necrotic reaction (Arthus reaction), severe urticaria, etc.
4. Diagnosis of immune deficiency diseases (such as congenital or acquired immunodeficiency, HIV infection, etc.), or receiving immunosuppressive/cytotoxic treatment (e.g., chemotherapy, organ transplantation, or planned treatment during the

clinical trial within 6 months before vaccination).

5. Subjects who received immunosuppressive therapy within 3 months before vaccination, or planning to receive such treatment within 1 month after full immunization (e.g., long-term use of systemic glucocorticoids for ≥ 14 days, dose ≥ 2 mg/kg/day or ≥ 20 mg/day prednisone or equivalence);

6. Receiving an inactivated vaccine, recombinant vaccine, or mRNA vaccine within 14 days or any live vaccine within 28 days prior to vaccination;

7. Subjects with acute illness or in the acute phase of a chronic disease within 3 days prior to vaccination.

8. Asplenia or splenectomy.

9. Subjects who receive blood products or immunoglobulin therapy within 3 months before vaccination, or planning to use such products within 2 months after vaccination.

10. Participating in other clinical studies of investigational or un-registered products (drugs, vaccines or devices, etc.), or planning to participate in other clinical studies before the end of this clinical study.

11. Any significant underlying medical condition (e.g., life-threatening diseases that may limit survival to less than 4 years) or other conditions that the investigator believes may interfere with the completion of the study.

Measurement of gE-Specific Antibody Concentrations

The levels of gE-specific IgG antibodies were determined by ELISA. gE protein (Sino Biological, Inc., Beijing, China) was diluted with coating buffer to a final concentration of 2 μg/mL and then used to coat 96-well microplates. After washing 3 times with phosphate-buffered saline-Tween (PBS-T) solution, the plates were blocked with 200 μL/well solution (1% bovine serum albumin [BSA] in phosphate-buffered saline [PBS]) at 37 ◦C for 1 h. After removing the blocking solution, the plates were incubated in threefold-diluted human serum (100 μL/well) starting at 50-fold at 37 ◦C for 1 h. After washing 4 times, horseradish peroxidase (HRP)-labeled anti-human IgG antibody was added to each well, and plates were incubated at 37 ◦C for 1 h. After washing 5 times,3,3′,5,5′-tetramethylbenzidine (TMB substrate (BD Biosciences, San Diego, CA, USA)) was added to the plate. Eight minutes later, the reaction was stopped, and the absorbance was measured at 450 nm with a microplate reader. The standard curve between antibody concentration and absorbance OD value was obtained by using varicella-zoster virus (VZV) antibody standard. The absorbance values of each well of the sample were substituted into the standard curve to calculate the antibody concentration in each well. If the deviation of antibody concentration between successive dilutions of the same sample did not exceed 30%, the mean value was used as the final antibody concentration. Anti-gE antibody cut-off value is 100 milli-International Units (mIU)/mL.

Flow Cytometry Analysis

Cryopreserved PBMCs were rapidly thawed at 37°C, resuspended in pre-warmed complete medium, and centrifuged (800 rpm, 10 min). Cell viability was determined using trypan blue exclusion, and density was adjusted to 2.0×10^6 cells/mL. Viability of thawed PBMCs, checked prior to testing by intracellular cytokine staining, was required to be >90%. PBMCs (2×10^6 cells/mL) were plated in 24-well plates (500 μL/well) and stimulated with recombinant VZV gE-His protein (200 μg/mL) for 20 h at 37°C under 5% CO_2. Protein Transport Inhibitor (containing Brefeldin A, 1 μ L/well) was added to test samples 4 h prior to harvest. Leukocyte Activation Cocktail

(BD GolgiPlug™, 2 μL/well) was used for positive controls. Cells were incubated with Human BD Fc Block™ (1:50 dilution) for 15 min at 4°C. Cell surface stained with anti-CD3-PE, CD4-FITC, CD8-APC-Cy7, and CD154-BB700 antibodies (30 min, 4°C). Cells were fixed using BD Cytofix/Cytoperm™ solution (20 min, 4°C), followed by two washes with Perm/Wash buffer. Intracellular stained with anti-IFN-γ-BV650, IL-2-PE-Cy7, and TNF-α-BV421 antibodies (30 min, 4°C). Stained cells were analyzed on a BD FACSCanto™ II flow cytometer equipped with FACSDiva™ software (v8.0.1). Finally, the cells were gated (FSC/SSC), and 10^5 T cells were analyzed with a FACS CantoTM flow cytometer (BD Biosciences, USA) and FlowJo X 10.0.7 R2 (Tree Star Inc., San Carlos, CA, USA). Recombinant gE was supplied by Beijing Luzhu Biotechnology Co., Ltd., Beijing, China.

Information of antibodies

	catalog number	clone number	lot number	manufacturer
FITC Mouse Anti-Human CD4	555346	RPA-T4	3152878	BD
PE Mouse Anti-Human CD3	555333	UCHT1	2238757	BD
APC-Cy™7 Mouse Anti-Human CD8	745814	TRAP1	3360086	BD
BV650 Mouse Anti-Human IFN-γ	563416	4S.B3	3278390	BD
PE-Cy™7 Rat Anti-Human IL-2	560707	MQ1-17H12	3306154	BD
BV421 Mouse Anti-Human TNF	562783	MAb11	3018685	BD
BB700 Mouse Anti-Human CD154	745814	TRAP1	3360085	BD
Human BD Fc Block™	564220	Fc1	3075820	BD

Table 1. The proportion of participants with positive responses to each cytokine in per-protocol set

	CD4+ T-cell responses			CD8+ T-cell responses		
	**LZ901 group n (%)**	**HZ/su group n (%)**	***p* value**	**LZ901 group n (%)**	**HZ/su group n (%)**	***p* value**
N	141	136		141	136	
IFN-γ	113 (80.1)	58 (42.6)	<0.0001	61 (43.3)	13 (9.6)	<0.0001
IL-2	114 (80.9)	91 (66.9)	0.01217	96 (68.1)	25 (18.4)	<0.0001
TNF-α	77 (54.6)	72 (52.9)	0.8745	21 (14.9)	10 (7.4)	0.0719
CD40L	40 (28.4)	19 (14.0)	0.0054	30 (21.3)	24 (17.6)	0.5415
⩾ 1 cytokine	138 (97.9)	118 (86.8)	0.0011	121 (85.8)	58 (42.6)	<0.0001

For each cytokine, a positive responder was defined as a 2-fold increase in cytokine-secreting T cells post-vaccination compared to pre-vaccination levels. Two-sided χ^2 test or Fisher's exact test was used for comparisons between two groups. In HZ/su group, there was one sample from each of 50-59 years and 60-69 years group that could not be tested due to insufficient cell quantity. IFN-γ, interferon-γ; IL-2, interleukin-2; TNF-α, tumor necrosis factor-α; CD40L, cluster of differentiation 40 ligand.

Table 2. The proportion of participants with positive responses to only 1 cytokine or simultaneous to any combination of 2, 3, or 4 cytokines producing CD4/CD8+ T cells 30 days after vaccination in per-protocol set

Combination of four cytokines	CD4+ T-cell responses			CD8+ T-cell responses		
	LZ901 group (N=141)	HZ/su group (N=136)	*p* value	LZ901 group (N=141)	HZ/su group (N=136)	*p* value
4 cytokines						
IFN-γ+IL-2+TNF-α+ CD40L	18 (12.8)	6 (4.4)	0.0240	1 (0.7)	0	>0.9999
3 cytokines						
IFN-γ+IL-2+TNF-α	40 (28.4)	29 (21.3)		10 (7.9)	1 (0.7)	
IFN-γ+IL-2+CD40L	7 (5.0)	1 (0.7)		8 (5.7)	0	
IFN-γ+TNF-α+CD40L	5 (3.6)	0		0	1 (0.7)	
IL-2+TNF-α+CD40L	1 (0.7)	1 (0.7)		1 (0.7)	0	
Total	53 (37.6)	31 (22.8)	0.0109	19 (13.48)	2 (1.5)	0.0004
2 cytokines						
IFN-γ+IL-2	27 (19.2)	12 (8.8)		20 (14.2)	1 (0.7)	
IFN-γ+TNF-α	6 (4.3)	7 (5.2)		4 (2.8)	5 (3.7)	
IFN-γ+CD40L	5 (3.6)	0		5 (3.5)	0	
IL-2+TNF-α	6 (4.3)	20 (14.7)		4 (2.8)	0	
IL-2+CD40L	2 (1.4)	2 (1.5)		13 (9.2)	4 (2.9)	
TNF-α+CD40L	0	1 (0.7)		0	0	
Total	46 (32.6)	42 (30.9)	0.8554	46 (32.6)	10 (7.4)	<0.0001
1 cytokine						
IFN-γ	5 (3.6)	3 (2.2)		13 (9.2)	5 (3.7)	
IL-2	13 (9.2)	20 (14.7)		39 (27.7)	19 (14.0)	
TNF-α	1 (0.7)	8 (5.9)		1 (0.7)	3 (2.2)	
CD40L	2 (1.4)	8 (5.9)		2 (1.4)	19 (14.0)	
Total	21 (14.9)	39 (28.7)	0.0002	55 (39.0)	46 (33.8)	0.4406
0 cytokine	3 (2.1)	18 (13.2)		20 (14.2)	78 (57.4)	<0.0001

Data are n (%): n = the number of participants with positive to only 1 cytokine or simultaneous to any combination of 2, 3, or 4 cytokines, % = proportion of participants; N= the number of samples analyzed; For each cytokine (IFN-γ, IL-2, TNF-α, or CD40L), a positive responder was defined as a 2-fold increase in cytokine-secreting T cells post-vaccination compared to pre-vaccination levels. Two-sided χ^2 test or Fisher's exact test was used for comparisons between two groups. In HZ/su group, there was one sample from each of 50-59 years and 60-69 years group that could not be tested due to insufficient cell quantity. IFN-γ, interferon-γ; IL-2, interleukin-2; TNF-α, tumor necrosis factor-α; CD40L, cluster of differentiation 40 ligand.

Table 3. The frequencies of cytokine-producing T cells per 10^5 $CD4^+$ T cells in per-protocol set

	LZ901 group (N=141)				HZ/su group (N=136)				*p* value (LZ901vs.Hz/su)	
	Day 0	Day 30	Fold increase	*p* value	Day 0	Day 30	Fold increase	*p* value	Day 0	Day 30
IFN-γ										
All ages	76.0 (49.0, 99.0)	198.0 (148.0,261.0)	2.68 (2.09, 3.78)	<0.0001	87.0 (64.0,120.0)	165.0 (114.0,226.0)	1.84 (1.11, 2.77)	<0.0001	0.0254	0.0007
50~59 years	73.0 (49.0, 99.0)	200.0 (157.0,242.0)	2.66 (2.01, 3.98)	<0.0001	87.0 (67.0, 115.0)	161.0 (108.0, 215.0)	1.76 (1.20, 2.56)	<0.0001	0.0417	0.0077
60~69 years	78.5 (56.5, 104.5)	197.0 (148.0,290.0)	2.68 (2.20, 3.69)	<0.0001	88.0 (54.0, 132.0)	170.5 (124.0, 229.0)	1.87 (1.07, 3.62)	<0.0001	0.4065	0.0301
≥70 years	74.5 (42.5, 97.5)	178.0 (116.0,264.5)	2.69 (2.03, 3.42)	<0.0001	87.0 (68.0, 117.0)	134.0 (113.0, 250.0)	2.32 (1.08, 3.02)	0.0044	0.4138	0.5817
IL-2										
All ages	66.0 (45.0, 105.0)	219.0 (142.0,308.0)	3.35 (2.18, 4.83)	<0.0001	79.0 (48.0, 111.0)	210.5 (126.0, 288.0)	2.71 (1.43, 4.92)	<0.0001	0.4038	0.3132
50~59 years	84.0 (50.0, 118.0)	238.0 (138.0,312.0)	3.22 (2.05, 4.40)	<0.0001	83.5 (48.0, 115.0)	218.0 (143.0, 294.0)	2.86 (1.42, 5.09)	<0.0001	0.9631	0.7055
60~69 years	58.5 (38.5, 84.0)	222.0 (153.5,309.5)	3.80 (2.76, 5.29)	<0.0001	74.0 (46.0, 97.0)	203.0 (122.0, 289.0)	2.69 (2.04, 4.21)	<0.0001	0.1583	0.4110
≥70 years	85.5 (49.5, 127.0)	190.0 (119.0,256.0)	2.65 (2.01, 4.23)	0.0033	83.0 (63.0, 134.0)	165.0 (95.0, 227.0)	1.71 (0.984,3.00)	0.0397	0.8709	0.4024
TNF-α										
All ages	129.0 (90.0, 186.0)	266.0 (187.0, 364.0)	2.18 (1.26, 3.03)	<0.0001	147.5 (102.0,193.0)	277.5 (206.5, 458.0)	2.07 (1.24, 3.06)	<0.0001	0.1269	0.0769
50~59 years	128.0 (90.0, 190.0)	265.0 (180.0, 354.0)	2.29 (1.40. 2.98)	<0.0001	151.5 (102.0,197.0)	286.0 (202.0, 459.0)	2.06 (1.11, 2.94)	<0.0001	0.1279	0.1637

60~69 years	128.5 (87.0, 163.0)	264.0 (182.5, 403.5)	2.13 (1.17, 3.37)	<0.0001	150.0 (102.0,206.0)	275.0 (225.0, 467.0)	2.02 (1.28, 3.07)	<0.0001	0.1887	0.1266
≥70 years	156.5 (118.0,188.0)	300.0 (249.0, 354.5)	1.97 (1.65, 3.00)	0.0005	127.0 (81.0, 150.0)	248.0 (211.0, 387.0)	2.18 (1.79, 3.05)	0.0003	0.1986	0.6899
CD40L										
All ages	42.0 (29.0, 66.0)	58.0 (35.0, 103.0)	1.47 (0.92, 2.08)	0.0003	49.5 (29.0, 83.0)	51.5 (33.5, 89.0)	0.96 (0.72, 1.22)	0.7513	0.3774	0.0227
50~59 years	43.0 (26.0, 72.0)	52.0 (36.0, 94.0)	1.41 (0.92, 2.08)	0.0140	49.0 (29.0, 85.0)	49.0 (33.0, 95.0)	0.96 (0.74, 1.54)	0.5543	0.4343	0.2529
60~69 years	40.5 (31.0, 64.0)	59.0 (31.5, 104.5)	1.55 (1.03, 1.88)	0.0196	57.0 (36.0, 85.0)	54.0 (38.0, 85.0)	0.97 (0.68, 1.14)	0.7465	0.1060	0.2785
≥70 years	46.0 (38.0, 68.0)	76.0 (46.0, 119.0)	1.71 (0.82, 2.23)	0.1932	35.0 (18.0, 44.0)	34.0 (19.0, 52.0)	1.07 (0.93, 1.14)	0.7467	0.0151	0.0129

Data shows the median (interquartile range, IQR); Fold increase indicates the median fold of the frequencies of cytokine-producing CD4+ T cells day 30 postvaccination to that baseline; Differences between groups were analyzed using a two-sided t test after log-transformed T-cell frequency; In HZ/su group, there was one sample from each of 50-59 years and 60-69 years group that could not be tested due to insufficient cell quantity. Day 0, baseline before vaccination; Day 30, day 30 after the second dose. IFN-γ, interferon-γ; IL-2, interleukin-2; TNF-α, tumor necrosis factor-α; CD40L, cluster of differentiation 40 ligand; PBMCs, peripheral blood mononuclear cells.

Table 4. The frequencies of cytokine-producing T cells per 10^5 $CD8^+$ T cells in per-protocol set

	LZ901 group (N=141)				HZ/su group (N=136)				*p* value (LZ901 *vs.*Hz/su)	
	Day 0	Day 30	Fold increase	*p* value	Day 0	Day 30	Fold increase	*p* value	Day 0	Day 30
IFN-γ										
All ages	197.0 (130.0,289.0)	347.0 (222.0,495.0)	1.90 (1.08,2.52)	<0.0001	216.5 (151.0,326.0)	213.0 (154.5,328.5)	0.995 (0.792,1.15)	0.8221	0.0697	<0.0001
50~59 years	202.0 (137.0,311.0)	340.0 (217.0,523.0)	1.88 (1.07, 2.41)	<0.0001	229.5 (151.0,351.0)	242.0 (163.0,348.0)	0.992 (0.823,1.10)	0.9706	0.0649	0.0030
60~69 years	196.0 (131.5,286.0)	358.5 (243.0,486.0)	1.82 (1.11, 2.55)	0.0002	198.0 (153.0,299.0)	200.5 (151.0,306.0)	0.998 (0.773, 1.22)	0.7488	0.9300	0.0002
≥70 years	158.0 (117.0,244.5)	328.0 (251.0,468.5)	2.35 (1.51, 2.70)	0.0003	177.0 (140.0,359.0)	185.0 (154.0,403.0)	1.04 (0.773, 1.12)	0.8714	0.2132	0.1368
IL-2										
All ages	97.0 (50.0, 164.0)	235.0 (140.0,376.0)	2.57 (1.61, 3.71)	<0.0001	110.5 (79.0, 152.0)	136.5 (83.5, 204.0)	1.12 (0.92, 1.54)	0.0024	0.1380	<0.0001
50~59 years	92.0 (50.0, 164.0)	268.0 (149.0,377.0)	2.92 (1.95, 3.93)	<0.0001	108.0 (84.0, 156.0)	150.0 (109.0,220.0)	1.61 (1.01, 1.70)	0.0065	0.0597	<0.0001
60~69 years	107.5 (55.0, 179.5)	225.0 (134.5,363.0)	2.23 (1.33, 3.42)	<0.0001	120.0 (68.0, 154.0)	134.5 (79.0, 186.0)	1.08 (0.89, 1.28)	0.1097	0.8562	<0.0001
≥70 years	89.5 (63.0, 163.5)	182.5 (123.5,265.5)	2.21 (1.05, 3.04)	0.0058	80.0 (53.0, 134.0)	82.0 (79.0, 135.0)	1.01 (0.768, 1.76)	0.6601	0.9650	0.0504
TNF-**α**										
All ages	514.0 (345.0,827.0)	598.0 (418.0,882.0)	1.01 (0.834,1.66)	0.1908	563.0 (395.0,906.0)	505.5 (331.5,746.0)	0.894 (0.612, 1.07)	0.0210	0.0652	0.0830
50~59 years	554.0 (347.0,811.0)	525.0 (330.0,803.0)	0.989 (0.791, 1.33)	0.9925	594.0 (395.0,986.0)	501.0 (306.0,756.0)	0.797 (0.561, 1.04)	0.0143	0.0583	0.6270
60~69 years	533.5 (348.5,840.0)	710.5 (461.5,1016.0)	1.03 (0.862, 1.92)	0.1433	555.0 (395.0,719.0)	542.5 (389.0,737.0)	0.948 (0.741, 1.06)	0.5399	0.6754	0.0905

≥70 years	452.5 (306.0,865.5)	675.0 (523.5, 865.5)	1.26 (0.943, 1.90)	0.2049	506.0 (416.0,1117.0)	464.0 (343.0,704.0)	0.925 (0.505, 1.57)	0.7501	0.5989	0.3502
CD40L										
All ages	68.0 (45.0, 120.0)	92.0 (46.0, 165.0)	1.41 (0.902, 1.98)	0.0108	67.0 (40.0, 114.0)	76.5 (38.0, 132.0)	0.993 (0.697, 1.59)	0.8007	0.9111	0.0298
50~59 years	74.0 (40.0, 115.0)	96.0 (49.0, 162.0)	1.52 (0.927, 2.00)	0.0224	67.5 (40.0, 119.0)	84.0 (42.0, 147.0)	1.01 (0.741, 1.82)	0.3815	0.9828	0.1732
60~69 years	70.5 (45.5, 127.5)	83.0 (44.5, 173.0)	1.34 (0.873, 1.87)	0.3039	64.0 (42.0, 107.0)	73.5 (35.0, 131.0)	0.956 (0.726, 1.32)	0.8563	0.4043	0.1448
≥70 years	53.0 (47.5, 91.5)	80.5 (35.5, 152.5)	1.43 (0.855, 1.86)	0.4303	83.0 (51.0, 142.0)	51.0 (38.0, 99.0)	0.758 (0.32, 1.59)	0.2656	0.2995	0.3272

Data shows the median (interquartile range, IQR); Fold increase indicates the median fold of the frequencies of cytokine-producing CD8+ T cells day 30 postvaccination to that baseline; Differences between groups were analyzed using a two-sided t test after log-transformed T-cell frequency. Day 0, baseline before vaccination; In HZ/su group, there was one sample from each of 50-59 years and 60-69 years group that could not be tested due to insufficient cell quantity. Day 30, day 30 after the second dose. IFN-γ, interferon-γ; IL-2, interleukin-2; TNF-α, tumor necrosis factor-α; CD40L, cluster of differentiation 40 ligand; PBMCs, peripheral blood mononuclear cells.

Table 5. The proportion of participants with simultaneous positive responses to ⩾2 cytokines in full-analysis set

	**LZ901 group n/N (%)**	**HZ/su group n/N (%)**	***p* value**
**The proportion of $CD4^{2+}$ T-cell responders**			
All age	120/146 (82.2)	79/137 (57.7)	<0.0001
50-59 years	59/75 (78.7)	42/74 (56.8)	<0.0001
60-69 years	47/53 (88.7)	29/50 (58.0)	<0.0001
⩾70 years	14/18 (77.8)	8/13 (61.5)	0.0578
**The proportion of $CD8^{2+}$ T-cell responders**			
All age	68/146 (46.6)	12/137 (8.8)	<0.0001
50-59 years	37/75 (49.3)	4/74 (5.4)	<0.0001
60-69 years	22/53 (41.5)	6/50 (12.0)	<0.0001
⩾70 years	9/18 (50.0)	2/13 (15.4)	0.001952

N= the number of participants included in the analysis; n=the number of participants with simultaneous positive responses to ⩾2 cytokines (referred to as $CD4^{2+}$ and $CD8^{2+}$ T-cell responder). Two-sided χ^2 test or Fisher's exact test was used for comparisons between two groups. In HZ/su group, there was one sample from each of 50-59 years and 60-69 years group that could not be tested due to insufficient cell quantity.

Table 6. Geometric mean concentrations and seroconversion rates of gE-specific IgG antibody in full-analysis set

	LZ901 group	HZ/su group	p value
All ages			
N	146	139	
GMC (Day 0)	1182.0 (1020.0, 1369.9)	1283.2 (1107.1, 1487.4)	0.4375
GMC (Day 30)	28715.8 (25950.2, 31776.1)	66711.1 (60813.3, 73180.9)	<0.0001
Seroconversion rate (%)	98.6 (95.1, 99.8)	100 (97.4, 100.0)	0.4985
50-59 years			
N	75	75	
GMC (Day 0)	1080.6 (888.5, 1314.4)	1138.9 (940.7, 1378.8)	0.7028
GMC (Day 30)	29580.5 (26069.2, 33564.8)	63869.7 (56583.8, 72093.8)	<0.0001
Seroconversion rate (%)	100 (95.2, 100.0)	100.0 (95.2, 100.0)	1.0000
60-69 years			
N	53	51	
GMC (Day 0)	1240.5 (953.3, 1614.2)	1462.7 (1110.1, 1927.2)	0.3877
GMC (Day 30)	28436.2 (24055.5, 33614.5)	73540.8 (62316.1, 86787.4)	<0.0001
Seroconversion rate (%)	96.2 (87.0, 99.5)	100.0 (93.0, 100.0)	0.4923
⩾70 years			
N	18	13	
GMC (Day 0)	1490.3 (933.0, 2380.4)	1528.5 (1016.5, 2298.4)	0.9311
GMC (Day 30)	26118.3 (16668.7, 40925.0)	58504.3 (42535.4, 80468.3)	0.004
Seroconversion rate (%)	100.0 (81.5, 100.0)	100.0 (75.3, 100.0)	0.3692

Data shown are the geometric mean (95% CI) for continuous variables and the percent (95% CI) for binary variables. N=the number of samples analyzed. Seroconversion was defined as at least a fourfold increase in the antibody concentration after vaccination compared to the baseline level. GMC=geometric mean concentration. For GMC, differences between groups were analyzed using a two-sided t test after log-transformed antibodies. For seroconversion rate, differences between groups

were analyzed using a two-sided χ^2 test or Fisher's exact test

Table 7. Multivariable Analysis of anti-gE antibody and CD4+/CD8+ T cells expressing cytokines 30 days after vaccination

Variables	D30 IgG		D30 IFN-γ$^+$CD4$^+$		D30 IL-2$^+$CD4$^+$		D30 TNF-α$^+$CD4$^+$		D30 CD40L$^+$CD4$^+$		D30 IFN-γ$^+$CD8$^+$		D30 IL-2$^+$CD8$^+$		D30 TNF-α$^+$CD8$^+$		D30 CD40L$^+$CD8$^+$	
	**β(SE)**	**p value**	**β(SE)**	**p value**	**β(SE)**	**P value**	**β(SE)**	**p value**	**β(SE)**	**p value**	**β(SE)**	**p value**	**β(SE)**	**p value**	**β(SE)**	**p value**	**β(SE)**	**p value**
Sex (reference: female)	0.004 (0.073)	0.955	0.034 (0.056)	0.543	-0.002 (0.068)	0.980	0.077 (0.061)	0.208	-0.017 (0.077)	0.829	0.041 (0.062)	0.510	-0.002 (0.063)	0.979	0.089 (0.065)	0.171	0.001 (0.083)	0.987
Age（reference:50-59 years）																		
60-69 years	0.022 (0.077)	0.777	0.092 (0.059)	0.121	0.005 (0.072)	0.941	0.059 (0.064)	0.359	-0.036 (0.082)	0.659	-0.012 (0.066)	0.854	-0.151 (0.067)	0.025	0.188 (0.068)	0.006	-0.108 (0.088)	0.217
≥70 years	-0.111 (0.121)	0.360	0.014 (0.093)	0.882	-0.215 (0.113)	0.057	0.170 (0.101)	0.092	-0.043 (0.128)	0.736	0.068 (0.104)	0.514	-0.287 (0.105)	0.007	0.224 (0.107)	0.038	-0.341 (0.137)	0.014
Vaccine group (reference: HZ/su)	**-0.815 (0.072)**	**<0.001**	**0.267 (0.055)**	**<0.001**	**0.141 (0.067)**	**0.036**	0.060 (0.060)	0.320	**0.255 (0.076)**	**<0.001**	**0.437 (0.062)**	**<0.001**	**0.640 (0.062)**	**<0.001**	**0.199 (0.064)**	**0.002**	**0.197 (0.082)**	**0.016**
Log(D0 IgG)	**0.163 (0.041)**	**<0.001**	0.053 (0.031)	0.093	0.043 (0.038)	0.262	-0.0009 (0.034)	0.980	0.021 (0.043)	0.634	0.039 (0.035)	0.264	0.099(0.035)	0.005	0.003 (0.036)	0.931	0.022 (0.046)	0.638
Log(D0 IFN-γ$^+$CD4$^+$)	-0.040 (0.075)	0.598	**0.291 (0.058)**	**<0.001**	0.019 (0.070)	0.790	-0.0009 (0.063)	0.989	-0.135 (0.080)	0.093	-0.074 (0.064)	0.252	0.055 (0.065)	0.399	-0.107 (0.067)	0.109	-0.202 (0.086)	0.019
Log(D0 IL-2$^+$CD4$^+$)	-0.012 (0.055)	0.827	0.037 (0.042)	0.379	**0.340 (0.051)**	**<0.001**	0.002 (0.046)	0.972	-0.026 (0.058)	0.659	0.085 (0.047)	0.072	0.102 (0.048)	0.033	0.110 (0.049)	0.025	0.033 (0.625)	0.596
Log(D0 TNF-α$^+$CD	0.046 (0.070)	0.510	0.070 (0.054)	0.195	-0.058 (0.065)	0.379	**0.316 (0.058)**	**<0.001**	0.005 (0.074)	0.949	-0.034 (0.060)	0.575	-0.088 (0.061)	0.151	-0.065 (0.062)	0.296	0.044 (0.080)	0.581

4^{+})																		
Log(D0 CD40L^{+}CD4^{+})	0.066 (0.056)	0.242	0.024 (0.043)	0.585	0.042 (0.053)	0.427	0.059 (0.047)	0.212	**0.683 (0.060)**	**<0.001**	0.023 (0.048)	0.637	-0.012 (0.049)	0.809	0.055 (0.050)	0.270	-0.041 (0.064)	0.522
Log(D0 IFN-γ^{+}CD8^{+})	0.019 (0.092)	0.839	0.086 (0.071)	0.223	-0.006 (0.086)	0.949	0.032 (0.077)	0.679	-0.061 (0.098)	0.535	**0.487 (0.079)**	**<0.001**	-0.007 (0.080)	0.927	-0.050 (0.082)	0.540	-0.129 (0.104)	0.221
Log(D0 IL-2^{+}CD8^{+})	-0.013 (0.056)	0.810	-0.016 (0.043)	0.711	0.102 (0.052)	0.049	-0.047 (0.046)	0.313	-0.058 (0.059)	0.327	0.028 (0.048)	0.560	**0.511 (0.048)**	**<0.001**	-0.069 (0.049)	0.166	0.003 (0.063)	0.958
Log(D0 TNF-α^{+}CD8^{+})	0.0003 (0.078)	0.997	-0.066 (0.060)	0.275	0.061 (0.073)	0.404	-0.069 (0.065)	0.291	0.086 (0.083)	0.302	0.073 (0.067)	0.278	0.102 (0.068)	0.133	**0.693 (0.069)**	**<0.001**	0.146 (0.089)	0.102
Log(D0 CD40L^{+}CD8^{+})	-0.034 (0.051)	0.507	-0.807 (0.039)	0.039	-0.036 (0.047)	0.447	-0.0007 (0.042)	0.987	-0.039 (0.054)	0.474	0.012 (0.043)	0.790	-0.026 (0.044)	0.563	0.080 (0.045)	0.076	**0.659 (0.058)**	**<0.001**

Generalized linear regression models were used to identify independent factors associated with gE-specific IgG antibodies and CMI responses at 30 days after vaccination, involving variables including vaccine group, sex, age group (50-59 years, 60-69 years and ≥70 years), pre-vaccination antibody levels and pre-vaccination CMI responses. Day 0, baseline before vaccination; Day 30, day 30 after the second dose.IFN-γ, interferon-γ; IL-2, interleukin-2; TNF-α, tumor necrosis factor-α; CD40L, cluster of differentiation 40 ligand.

Table 8. List of severe adverse events

Group	Disease	Time of vaccination	Start time	End time	Outcome	Correlation with vaccination
LZ901	Bronchiectasis with infection, candida pneumonia, type II respiratory failure	2023/12/25	2024/2/4	2024/2/16	Improvement	NO
LZ901	Hepatocellular carcinoma	2023/12/26	2024/5/9	2024/8/28	Improvement	NO
LZ901	Incarcerated inguinal hernia	2023/12/27	2024/5/10	2024/5/18	Recovery	NO
LZ901	Coronary atherosclerotic cardiopathy	2023/12/29	2024/3/27	2024/4/2	Improvement	NO
HZ/su	hypohepatia	2023/12/26	2024/8/5	2024/8/20	Recovery	NO
HZ/su	Lung abscess	2023/12/26	2024/1/29	2024/5/20	Recovery	NO
HZ/su	Polypofcolon	2023/12/27	2024/7/17	2024/7/23	Recovery	NO
HZ/su	Enign tumor of hepatic flexure of colon	2023/12/28	2024/7/20	2024/7/25	Recovery	NO

Table 9. List of the second dose beyond the allowed window

ID	Group	age	Date of 1stdose	Date of 2nd dose	Reasons
D101-019	LZ901	58	2023/12/25	2024/2/26	acute upper respiratory infections
D101-063	LZ901	74	2023/12/26	2024/2/26	acute upper respiratory infections
D101-065	LZ901	73	2023/12/26	2024/2/7	acute upper respiratory infections
D101-123	LZ901	58	2023/12/27	2024/2/26	acute upper respiratory infections
D101-128	LZ901	68	2023/12/27	2024/2/7	acute upper respiratory infections
D101-008	HZ/su	58	2023/12/25	2024/3/2	acute upper respiratory infections

Fig.1 gE-specific IgG antibody concentrations according to age

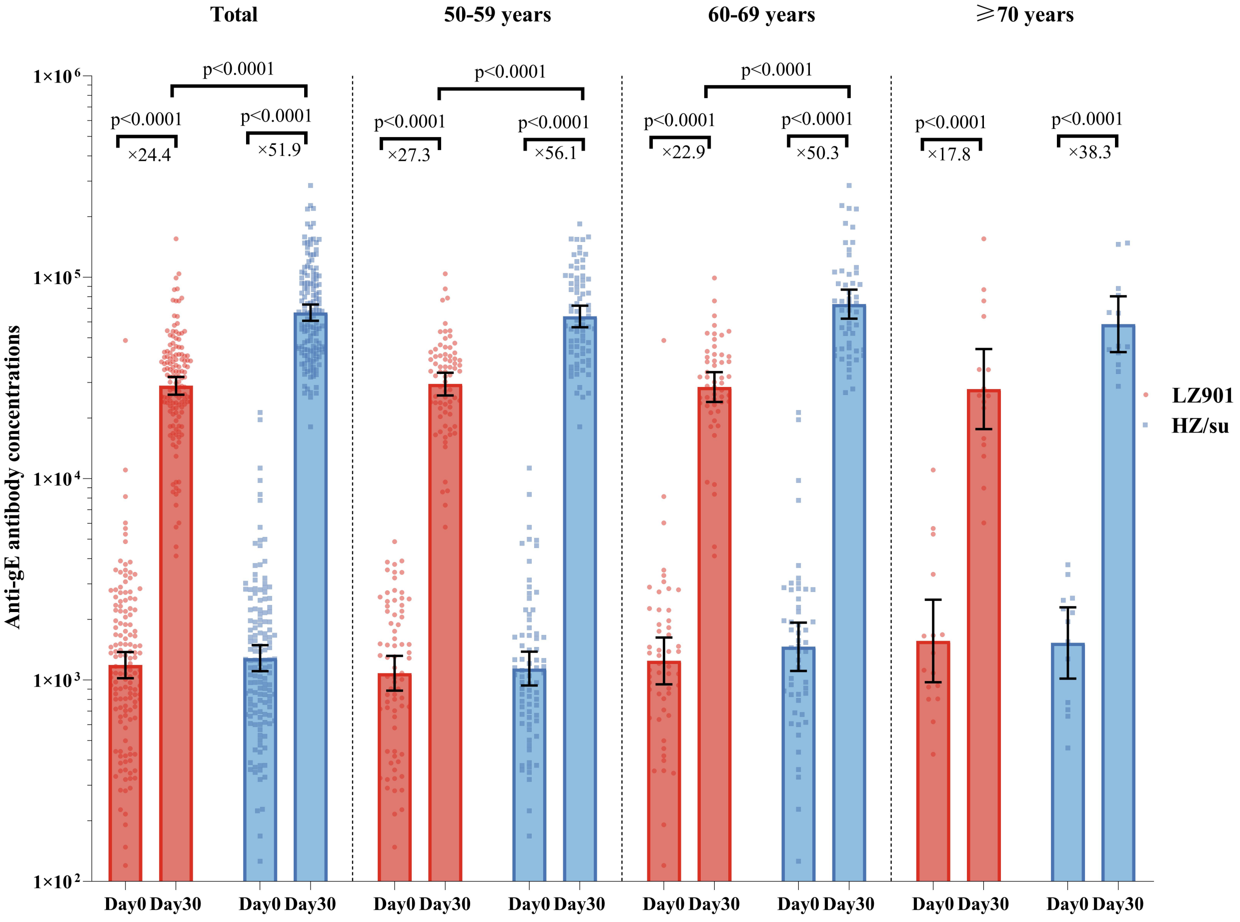

gE-specific IgG antibody concentrations according to age in the LZ901 group (n=141) and HZ/su group (n=138). Error bars represent 95% confidence intervals. Differences between groups were analyzed using a two-sided t test after log-transformed antibodies. The number under p value indicate that geometric mean fold increase (GMFI) of gE-specific IgG antibody day 30 after the second dose to that baseline. Day 0, baseline before vaccination; Day 30, day 30 after the second dose.

Fig.2 Correlation matrices of antibody levels with cell-mediated immunity responses to LZ901 vaccine and HZ/su vaccine 30 days after vaccination.

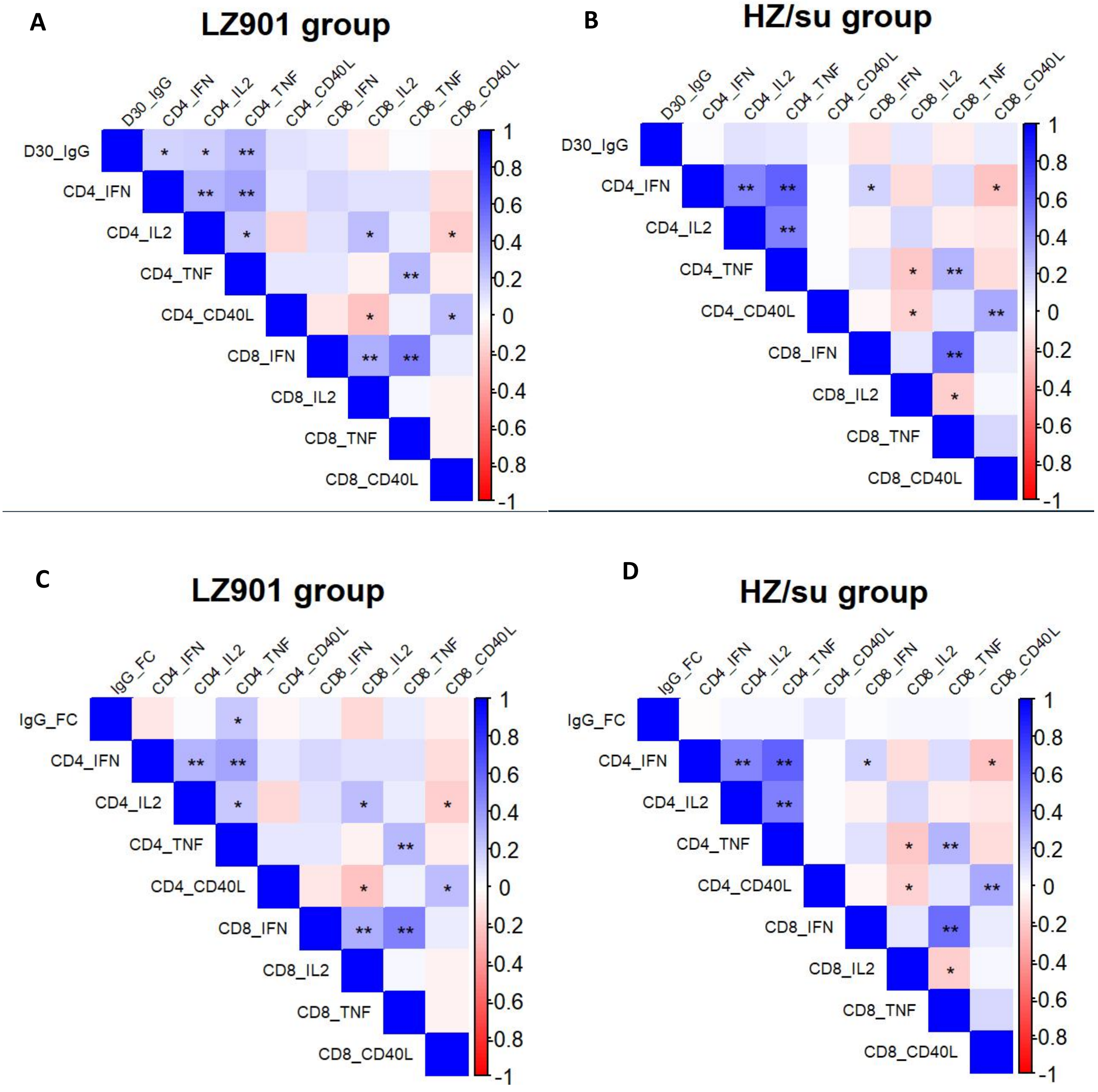

A, B The correlation of antibody concentration with CD4+/CD8+ T-cell response. **C, D** The correlation of antibody fold-rise with CD4+/CD8+ T-cell response. Correlation analyzes were performed using Spearman's rank correlation with the two-tailed p-value. Colors indicate Spearman's correlation coefficient. *$p < 0.05$; **$p < 0.01$; blank cells: $p > 0.05$. FC, fold change; IFN-γ, interferon-γ; IL-2, interleukin-2; TNF-α, tumor necrosis factor-α; CD40L, cluster of differentiation 40 ligand.

Fig.3 Solicited adverse reactions that occurred within 30 days after each dose

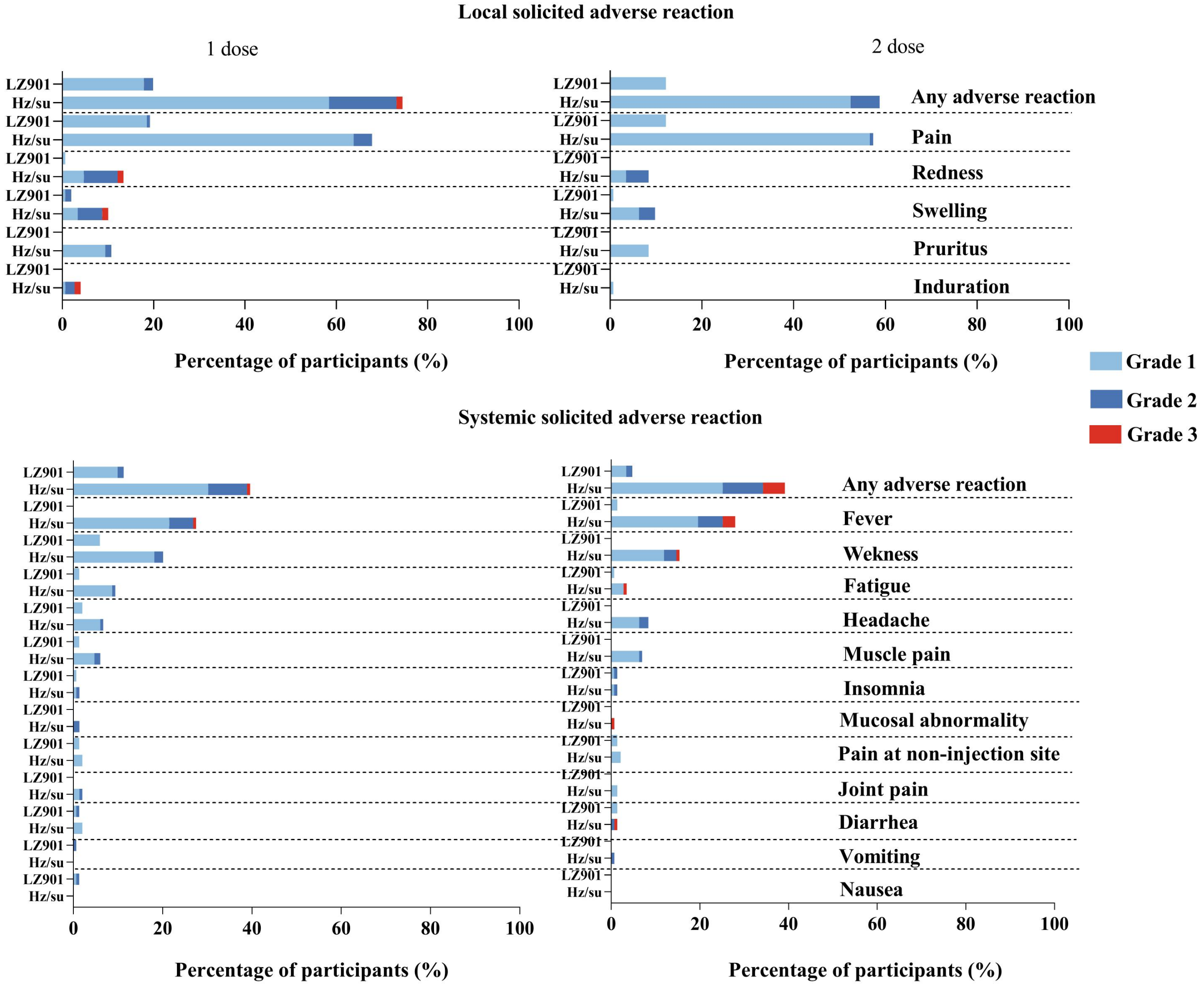

Fig.4 Gating strategy

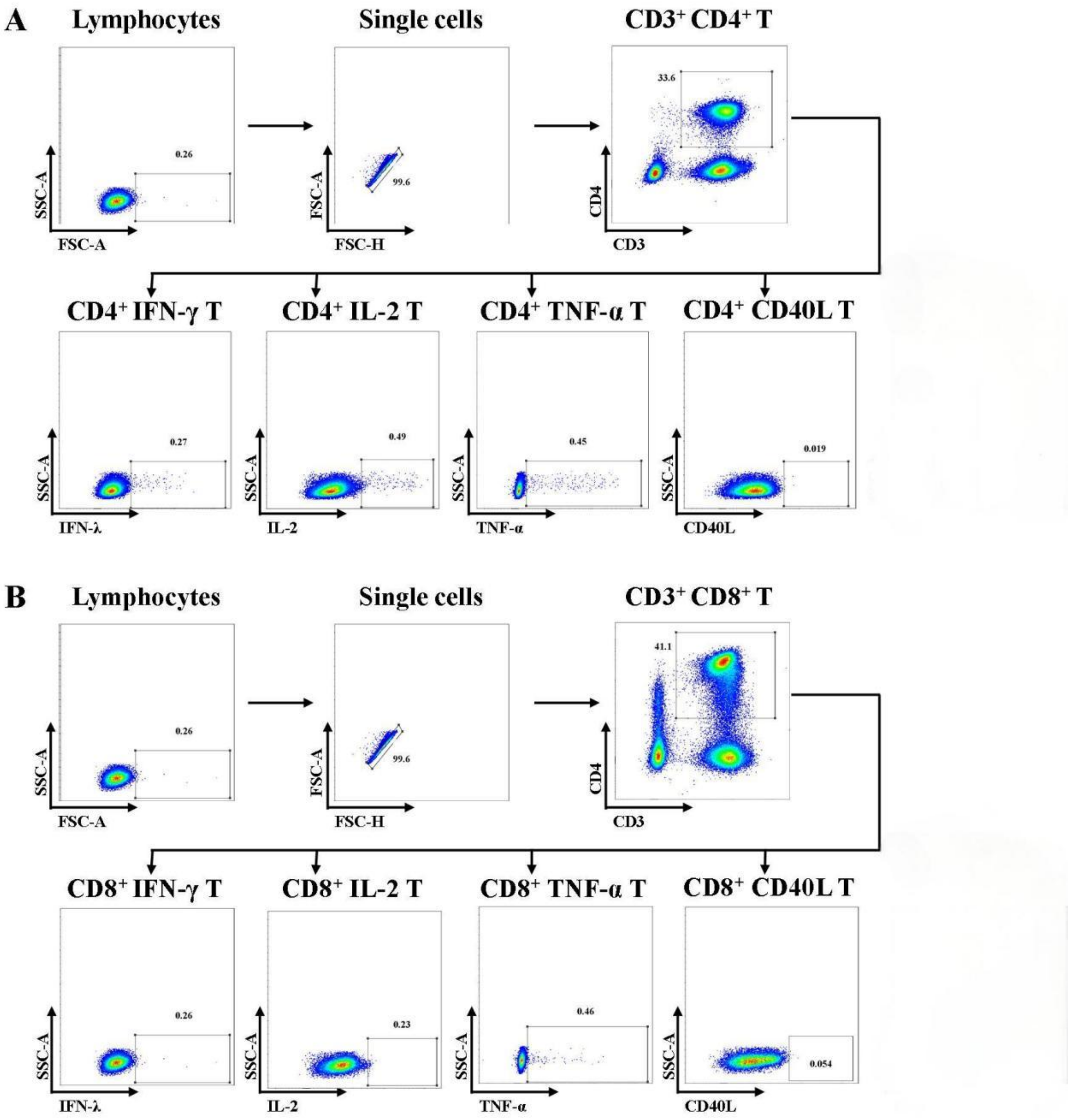

A Gating strategy to identify CD4+ T-cell expressing IFN-γ, IL-2,TNF-αand CD40L. **B** Gating strategy to identify CD8+ T-cell expressing IFN-γ, IL-2, TNF-α and CD40L. IFN-γ, interferon-γ; IL-2, interleukin-2; TNF-α, tumor necrosis factor-α; CD40L, cluster of differentiation 40 ligand.

CONSORT 2010 checklist of information to include when reporting a randomised trial*

Section/Topic	Item No	Checklist item	Reported on page No
**Title and abstract**			
	1a	Identification as a randomised trial in the title	Page 1
	1b	Structured summary of trial design, methods, results, and conclusions (for specific guidance see CONSORT for abstracts)	Page 3
**Introduction**			
Background and objectives	2a	Scientific background and explanation of rationale	Page 4-5
	2b	Specific objectives or hypotheses	Page 5
**Methods**			
Trial design	3a	Description of trial design (such as parallel, factorial) including allocation ratio	Page 13
	3b	Important changes to methods after trial commencement (such as eligibility criteria), with reasons	NA
Participants	4a	Eligibility criteria for participants	Page 13, Supplementary methods
	4b	Settings and locations where the data were collected	Page 13
Interventions	5	The interventions for each group with sufficient details to allow replication, including how and when they were actually administered	Page 14
Outcomes	6a	Completely defined pre-specified primary and secondary outcome measures, including how and when they were assessed	Page 14-16
	6b	Any changes to trial outcomes after the trial commenced, with reasons	NA
Sample size	7a	How sample size was determined	Page 16
	7b	When applicable, explanation of any interim analyses and stopping guidelines	NA
Randomisation:			

Sequence generation	8a	Method used to generate the random allocation sequence	Page 14
	8b	Type of randomisation; details of any restriction (such as blocking and block size)	Page 14
Allocation concealment mechanism	9	Mechanism used to implement the random allocation sequence (such as sequentially numbered containers), describing any steps taken to conceal the sequence until interventions were assigned	Page 14
Implementation	10	Who generated the random allocation sequence, who enrolled participants, and who assigned participants to interventions	Page 14
Blinding	11a	If done, who was blinded after assignment to interventions (for example, participants, care providers, those assessing outcomes) and how	Page 14
	11b	If relevant, description of the similarity of interventions	NA
Statistical methods	12a	Statistical methods used to compare groups for primary and secondary outcomes	Page 16-17
	12b	Methods for additional analyses, such as subgroup analyses and adjusted analyses	Page 17
**Results**			
Participant flow (a diagram is strongly recommended)	13a	For each group, the numbers of participants who were randomly assigned, received intended treatment, and were analysed for the primary outcome	Page 5 Figure 1
	13b	For each group, losses and exclusions after randomisation, together with reasons	Page 5 Figure 1
Recruitment	14a	Dates defining the periods of recruitment and follow-up	Page 5
	14b	Why the trial ended or was stopped	NA
Baseline data	15	A table showing baseline demographic and clinical characteristics for each group	Table 1
Numbers analysed	16	For each group, number of participants (denominator) included in each analysis and whether the analysis was by original assigned groups	Figure 1
Outcomes and estimation	17a	For each primary and secondary outcome, results for each group, and the estimated effect size and its precision (such as 95% confidence interval)	Page 6-9
	17b	For binary outcomes, presentation of both absolute and relative effect sizes is recommended	Page 5-9
Ancillary analyses	18	Results of any other analyses performed, including subgroup analyses and adjusted analyses, distinguishing pre-	Page 7-8

		specified from exploratory	
Harms	19	All important harms or unintended effects in each group (for specific guidance see CONSORT for harms)	NA
**Discussion**			
Limitations	20	Trial limitations, addressing sources of potential bias, imprecision, and, if relevant, multiplicity of analyses	Page 12
Generalisability	21	Generalisability (external validity, applicability) of the trial findings	Page 13
Interpretation	22	Interpretation consistent with results, balancing benefits and harms, and considering other relevant evidence	Page 9-11
**Other information**			
Registration	23	Registration number and name of trial registry	Page 13
Protocol	24	Where the full trial protocol can be accessed, if available	Protocol v1.1
Funding	25	Sources of funding and other support (such as supply of drugs), role of funders	Page 20

*We strongly recommend reading this statement in conjunction with the CONSORT 2010 Explanation and Elaboration for important clarifications on all the items. If relevant, we also recommend reading CONSORT extensions for cluster randomised trials, non-inferiority and equivalence trials, non-pharmacological treatments, herbal interventions, and pragmatic trials. Additional extensions are forthcoming: for those and for up to date references relevant to this checklist, see www.consort-statement.org.

Clinical Trial Protocol

A randomized, blinded clinical trial to evaluate the immunogenicity and safety of the recombinant zoster vaccine (CHO cells) LZ901 compared with a active vaccine in subjects aged 50 years and older

Registration Category:	Preventive biologics
Protocol Number:	LZ901-330
Version No.:	V1.1
Version Date:	Dec.8, 2023
Sponsor:	Beijing Luzhu Biotechnology Co., Ltd.<br>Luzhu Biopharmaceuticals (Zhuhai) Co., Ltd.

Version	Data
V1.0	Aug.5, 2023
V1.1	Dec.8, 2023

CONFIDENTIALITY STATEMENT

The information contained in this document, especially unpublished data, is the property of Beijing Luzhu Biotechnology Co., Ltd. / Luzhu Biopharmaceuticals (Zhuhai) Co., Ltd. (hereinafter referred to as the "Sponsor") or under the control of the Sponsor, and is hereby provided in a confidential form to you as the investigator, potential investigator, or consultant for review by you, your staff, and relevant Ethics Committee. All information will be used only when you are authorized to conduct clinical studies with the investigation vaccine described in this protocol. Except for the information that needs to be introduced to a certain extent to the people who may use the study vaccine when obtaining informed consent, no information shall be disclosed to others without the written permission of the sponsor.

SPONSOR SIGNATORY

PROTOCOL TITLE: A randomized, blinded clinical trial to evaluate the immunogenicity and safety of the recombinant zoster vaccine (CHO cells) LZ901 compared with a active vaccine in subjects aged 50 years and older

PROTOCOL No:LZ901-330

VERSION: 1.1

This Protocol has been approved by

Beijing Luzhu Biotechnology Co., Ltd./Luzhu Biopharmaceuticals (Zhuhai) Co., Ltd.

Name: Date

Title:

Beijing Luzhu Biotechnology Co., Ltd

Luzhu Biopharmaceuticals (Zhuhai) Co., Ltd.

SIGNATURE OF INVESTIGATOR

PROTOCOL TITLE: A randomized, blinded clinical trial to evaluate the immunogenicity and safety of the recombinant zoster vaccine (CHO cells) LZ901 compared with a active control vaccine in subjects aged 50 years and older

PROTOCOL NO: LZ901-330

VERSION: 1.1

This protocol is a confidential communication of the Sponsor. I confirm that I have read this protocol, I understand it, and I will work according to this protocol. I will also work consistently with the ethical principles that have their origin in the Declaration of Helsinki and that are consistent with Good Clinical Practices (GCPs) and the applicable laws and regulations. Acceptance of this document constitutes my agreement that no unpublished information contained herein will be published or disclosed without prior written approval from the Sponsor.

I have read this protocol in its entirety and agree to conduct the study accordingly:

Signature of Investigator: ______________________ Date: ________

Printed Name: ___________________________

Investigator Title: ________________________

Name/Address of Center:

Jiangsu Provincial Center for Disease Control and Prevention

172 Jiangsu Road, Nanjing

Contents

SPONSOR SIGNATORY 2
SIGNATURE OF INVESTIGATOR 3
Contents 4
List of Abbreviations 9
Definitions of Terms 11
Protocol Synopsis 12
Schedule of Activities 18
1. Introduction 20
1.1 Background 20
1.2 Benefits and Risks 23
1.2.1 Benefits 23
1.2.2 Risks 23
1.2.3 Summary of benefits and risks 24
2. Study objectives and endpoints 25
2.1 Study Objectives 25
2.1.1 Primary objective 25
2.1.2 Secondary objective 25
2.2 Study Endpoints 25
2.2.1 Primary endpoints 25
2.2.2 Secondary endpoints 25
3. Study design 25
3.1 Overall Design 25
3.1.1 Sample Collection 26
3.1.1.1 Blood sample collection 26
3.1.1.2 Collection of Biological Samples for Suspected Herpes Zoster Cases 26
3.1.1.3 Urine sample collection (pregnancy test) 27
3.1.2 Vaccination 27
3.1.3 Follow-up visit 27
3.1.3.1 Immunogenicity observation 27
3.1.4 Study period 27
3.2 Rationale for the Selection of the Study Design 28
3.2.1 Rationale for the Selection of the Control Vaccine 28
3.2.2 Preclinical trial data 28
3.2.3 Phase I/II Clinical Trial Data 30
3.3 Start and end of the study 32
4. Study Population 32
4.1 The selection of the study population 32

4.2 The selection basis for the study population ... 32
4.3 Inclusion criteria ... 32
4.4 Exclusion criteria ... 32
4.5 Screening Failure ... 33
5. Study intervention ... 34
5.1 Study intervention procedure ... 34
5.1.1 Subject recruitment ... 34
5.1.2 Informed Consent ... 34
5.1.3 Screening ... 34
5.1.4 Randomization of Subjects ... 34
5.1.5 Sample Collection ... 35
5.1.6 The first dose of vaccination ... 35
5.1.7 The second dose of vaccination ... 35
5.1.8 Medical Observation ... 36
5.1.9 Safety Follow-up ... 36
5.1.10 Concomitant Medications ... 37
5.2 Basis for Intervention Selection ... 38
5.3 Dosage and Administration ... 39
5.4 Preparation, Handling, Storage, and Counting ... 39
5.4.1 Preparation of Study Intervention ... 39
5.4.2 Blinding of Study Interventions ... 40
5.4.3 Handling and Storage of Study Interventions ... 40
5.4.4 Packaging and Labeling ... 41
5.4.5 Inventory of Study Interventions ... 41
5.4.6 Reserve vaccine ... 42
5.5 Subject Assignment, Randomization, and Blinding ... 42
5.5.1 Randomization of Subjects ... 42
5.5.2 Randomization ... 42
5.5.3 Blinding and Unblinding ... 42
5.6 Study Intervention Compliance ... 43
6. Discontinuation of Study Intervention and Subject Withdrawal from the Study ... 44
6.1 Discontinuation of Study Intervention ... 44
6.1.1 Postponement Criteria for the Second Vaccination ... 44
6.1.2 Exclusion Criteria for the Second Dose ... 44
6.2 Subject Withdrawal from Study ... 44
6.2.1 Criteria for Subject Withdrawal from Study ... 44
6.2.2 Reasons for Subject Withdrawal ... 45
6.3 Discontinuation of Study Vaccine ... 45
6.4 Loss to Follow-Up ... 46
6.5 Criteria for study suspension ... 46

6.6 Criteria for study termination 46
7. Study Evaluation and Procedures 46
7.1 Screening Evaluation and Procedures 47
7.2 Immunogenicity Evaluation and Procedures 47
7.3 Safety Evaluation and Procedures 47
7.3.1 Physical Examination 47
7.3.2 Temperature Check 47
7.3.3 Safety Assessment 47
7.4 Adverse events and serious adverse events 48
7.4.1 Definitions of AE and SAE 48
7.4.2 Identification and Documentation of AEs and SAEs 48
7.4.3 Follow-up for AEs and SAEs 49
7.4.4 SAE Reporting 49
7.4.5 SAE Regulatory Reporting Requirements 50
7.4.6 Suspected Unexpected Serious Adverse Reaction (SUSAR) 50
7.4.7 Adverse Events of Special Interest (AESIs) 51
7.4.8 Disease-Related Events Not Meeting AE or SAE Criteria 51
7.5 Pregnancy and Postpartum Information 51
7.5.1 Subjects Who Become Pregnant During the Study 51
7.6 Immunogenicity Evaluation 52
7.6.1 Biological Sample Collection 52
8. Statistical Considerations 53
8.1 Statistical Hypotheses 53
8.2 Sample Size Determination 54
8.3 Analysis Sets 54
8.4 Analysis Methods 55
8.4.1 General Principles 55
8.4.2 Cellular Immunogenicity 55
8.4.3 Humoral Immunogenicity 55
8.5 Protocol Deviations 56
9. General Considerations: Regulatory, Ethical, and Trial Oversight 57
9.1 Regulatory and Ethical Considerations 57
9.1.1 Investigator Responsibilities 58
9.1.2 Sponsor Responsibilities 58
9.2 Ethics Committee 59
9.2.1 Follow-Up Review 59
9.2.2 Amendment Review 59
9.2.3 Annual/Periodic Follow-Up Review 59
9.2.4 Serious Adverse Event Review 60
9.2.5 Non-Compliance/Protocol Violation Review 60

9.2.6 Premature Termination Review 60
9.2.7 Study Completion Review 60
9.3 Informed Consent Process 60
9.4 Early Closure of Research Center or Study Termination 61
10. General Considerations: Risk Management and Quality Assurance 61
10.1 Data Quality Assurance 61
10.1.1 Investigator 61
10.1.2 Sponsor 61
10.1.3 Clinical Research Associate (CRA) 62
10.1.4 Protocol Amendments 62
10.1.5 Biological Sample Management 62
10.1.6 Vaccine Management 63
10.1.7 Instrument and Equipment Calibration 64
10.1.8 Original Data Management 64
10.1.9 Study Documentation 64
10.2 Source Data 65
10.2.1 eCRF Design 65
10.2.2 eCRF Completion Guidelines 65
10.2.3 eCRF Annotation 65
10.2.4 Database Design 66
10.2.5 Permission Allocation 66
10.2.6 eCRF Completion 66
10.2.7 Query Issuance and Resolution 66
10.2.8 Data Modification and Review 66
10.2.9 Medical Coding 66
10.2.10 SAE Consistency Comparison 67
10.2.11 Data Review Meeting 67
10.2.12 Database Lock and Unlock 67
10.2.13 Data Backup and Recovery 67
10.2.14 Data Storage 67
10.2.15 Data Confidentiality 67
11. Appendix: Adverse Events (AEs) and Serious Adverse Events (SAEs) – Definitions, Severity, and Causality 68
11.1 Further Details and Explanations on the Definition of AE 68
11.2 Further Details and Explanations on the Definition of SAE 68
11.3 Severity 68
11.4 Causality 72
12. Appendix: Definitions and Supportive Operational Details 73
12.1 Contraception and Pregnancy Testing 73
12.1.1 Definitions Related to Fertility 73

12.1.2 Contraception 73
12.1.3 Pregnancy Testing 74
13. Disclosure and Publication of Data 74
14. Reference 74

List of Abbreviations

Abbreviation	Expanded Term
AE	Adverse Event
AESI	Adverse Events of Special Interest
NMPA	National Medical Products Administration
CDC	Centers for Disease Control
CEC	Clinical Event Committee
CRF	Case Report Form
CHO cell	Chinese Hamster Ovary cell
CRO	Contract Research Organization
eCRF	Electronic Case Report Form
EDC	Electronic data capture
IRB	Institutional Review Board
FAS	Full Analysis Set
GCP	Good Clinical Practice
GMC	Geometric Mean Concentration
GMFI	Geometric Mean Fold Increase
HZ	Herpes Zoster
MedDRA	Medical Dictionary for Regulatory Activities
PCR	Polymerase Chain Reaction
PHN	Post-herpetic Neuralgia
PPS	Per Protocol Set
PT	Preferred Term
PV	Pharmacovigilance
qPCR	Real-time Quantitative PCR Detecting System
SAE	Serious Adverse Event
SOC	System Organ Class
SOP	Standard Operation Procedure
SS	Safety Set

Abbreviation	Expanded Term
SUSAR	Suspected Unexpected Serious Adverse Reaction
TEAE	Treatment Emergent Adverse Event
VZV	Varicella Zoster Virus
WHODD	WHO Drug Dictionary
ZBPI	Zoster Brief Pain Inventory

Definitions of Terms

Month	30 days.
Women of Childbearing Age	Women from menarche until menopause are considered capable of childbearing unless it has been clearly confirmed that they have undergone a hysterectomy, bilateral salpingectomy, and bilateral oophorectomy.
Severe Medical History	Diseases requiring emergency treatment, hospitalization, and/or surgical procedures.
Antibody Positivity Rate and Antibody Seroconversion Rate	1) Abnormal antibody concentration (measured by ELISA): Specific anti-gE antibody concentration >0.1 IU/mL (or 100 mIU/mL) is defined as positive.<br>2) Pre-immunization antibody positivity rate: The proportion of subjects with anti-gE antibody positivity in pre-immunization serum samples.<br>3) Post-immunization antibody concentration ≥4 times the pre-immunization level is defined as seroconversion.<br>4) Seroconversion rate: The proportion of subjects achieving seroconversion in post-immunization serum samples.
Antibody Fold Increase	The fold increase in specific anti-gE antibody concentration (measured by ELISA) compared to the pre-immunization level.
HZ Suspected Cases	Suspected herpes zoster (HZ) cases meet at least one of the following criteria:<br>a) Unilateral pain or rash.<br>b) Dermatomal, unilateral distribution of grouped vesicles on an erythematous base.<br>c) Neuralgia.

Protocol Synopsis

Study title	A randomized, blinded clinical trial to evaluate the immunogenicity and safety of the recombinant zoster vaccine (CHO cells) LZ901 compared with a similar active vaccine in subjects aged 50 years and older
Sponsor	Beijing Luzhu Biotechnology Co., Ltd.<br>Luzhu Biopharmaceuticals (Zhuhai) Co., Ltd.
Protocol No.	LZ901-330, V1.1
Study phase	III+
Study vaccine	Recombinant zoster vaccine (CHO cells) (LZ901)
	Recombinant Zoster Vaccine (RZV, Shingrix™)
Indications	To prevent herpes zoster (HZ) and its complications caused by Varicella-Zoster Virus (VZV) infection.
**Study Objectives**	
Primary objective	To evaluate the immunogenicity of cellular immune response induced by recombinant zoster vaccine (CHO cell) in subjects aged 50 years and older, in comparison to the active control vaccine.
Secondary objective	To evaluate the immunogenicity and safety of humoral immune responses induced by recombinant zoster vaccine (CHO cells) in subjects aged 50 years and older.
**Study Endpoints**	
Primary endpoints	The proportion of specific CD4+/CD8+ T cells activating at least two cytokines (TNF-α, IFN-γ, IL-2, CD40L) 30 days after full immunization.
Secondary endpoints	**Immunogenicity endpoints**<br>1. Geometric Mean Concentration (GMC) and seroconversion rate of anti-gE antibodies 30 days after full immunization;<br>2. Geometric Mean Fold Increase (GMFI) in anti-gE antibodies 30 days after the whole immunization course compared to pre-immunization (Day 0).<br>**Safety endpoints**<br>1. Incidence of solicited adverse events (AE) within 0-7 days after each dose, including local (injection site) and systemic (non-injection site) reactions;<br>2. Incidence of unsolicited AEs within 0-30 days after each dose;<br>3. Incidence of AEs within 30 minutes after each dose;<br>4. Incidence of adverse events of special interest (AESIs) from the first dose through 30 days after full immunization;<br>5. Incidence of serious adverse events (SAEs) from the first dose through 6 months after full immunization.
**Study Design**	
Overall design	**Study design:** Randomized, partially blinded<br>**Immunization schedule:** Experimental vaccine at 0 and 30 days; active control vaccine at 0 and 60 days

Study groups: Experimental group and control group

Blinding: LZ901-330 is a partially blinded study, with a double-blind design prior to the second dose, and an open-label design at the time of the second dose.

Number of subjects and groups

Approximately 300 subjects aged 50 and older will be enrolled and randomly assigned in a 1:1 ratio to the experimental group and the control group. The experimental group will receive two doses of the recombinant zoster vaccine (CHO cell), developed and manufactured by Beijing Luzhu Biotechnology Co., Ltd. / Luzhu Biopharmaceuticals (Zhuhai) Co., Ltd., with an immunization schedule of 0 and 30 days. The control group will receive two doses of the Recombinant Zoster Vaccine (RZV, Shingrix^{TM}), products of GlaxoSmithKline Biologicals SA, with an immunization schedule of 0 and 60 days.

Table 1 Treatment Groups

Age	Experimental group	Control group	Total target enrollment
50 and older	150	150	300

Vaccine administration

Subjects in the experimental group will be administered two doses of the experimental vaccine on days 0 and 30, while subjects in the control group will be administered two doses of the control vaccine on days 0 and 60.

Study follow-up visit

The specific visit schedule and visit content are detailed in the "Study Schedule."

Immunogenicity observation: Blood samples will be collected from all subjects once before vaccination and once 30 days after full immunization, for a total of two blood samples. The ELISA method will be used to measure varicella-zoster virus (VZV) gE-specific antibodies, and flow cytometry will be used to assess VZV gE-specific CD4+/CD8+ T cell activation. The proportion of subjects activating at least two cytokines (TNF-α, IFN-γ, IL-2, CD40L) will be calculated to evaluate the vaccine humoral and cellular immune response. Detailed blood sample collection time points, volumes, and purposes are shown in the table below:

Table 2 Detailed blood sample collection time, volumes, and purposes

Visit Time	Test items and blood collection volume（ml）		
	Serum Antibody	Cellular Immune	Total
D0	4.0-5.0	15.0-20.0	19.0-25.0
30 days after full immunization	4.0-5.0	15.0-20.0	19.0-25.0

Note: Serum antibody testing is performed using procoagulant tubes for blood collection; cellular immunity indicators are tested using anticoagulant tubes for blood collection.

Safety observation:

Collect all AEs (adverse events) within 30 days after vaccination (including solicited AEs within 30 minutes, 0-7 days, and all unsolicited AEs within 0-30 days). Follow up on all SAEs (serious adverse events) and pregnancy-related

<table>
<tr><td></td><td>events from the first dose of vaccination to 6 months after full immunization.
Solicited AEs include:
<table>
<tr><td>Injection site (local) AEs</td><td>Pain，pruritus，induration，swelling，rash，redness</td></tr>
<tr><td>Non-injection site (systemic) AEs</td><td>Fatigue, tiredness, fever, headache, insomnia, myalgia, skin and mucosal abnormalities, pain at non-injection sites
, diarrhea, constipation, vomiting, nausea, acute allergic reactions, arthritis, arthralgia</td></tr>
</table>
Unsolicited AEs include: Any adverse event that is not a solicited AE or any solicited AE occurring outside the solicitation period (e.g., If the solicited AEs mentioned above occur more than 7 days after vaccination, they should be recorded as unsolicited AEs).
Collection of AESIs (Adverse Events of Special Interest):
Suspected cases of herpes zoster within 30 days following the first dose of vaccination to full immunization.</td></tr>
<tr><td rowspan="2">study vaccine</td><td>Experimental Vaccine:
Vaccine Name: Recombinant zoster vaccine (CHO Cells)
Specification: 100μg/0.5ml/ vial
Lot Number: 20230201
Manufacturer: Luzhu Biopharmaceuticals (Zhuhai) Co., Ltd.</td></tr>
<tr><td>Control Vaccine:
Vaccine Name: Recombinant Zoster Vaccine (RZV, Shingrix™)
Specification: 50 μg/0.5 ml/vial
Lot Numbers: 3472D, X4937
Manufacturer: GlaxoSmithKline Biologicals SA</td></tr>
<tr><td colspan="2">Study Population</td></tr>
<tr><td>Age</td><td>Aged 50 and older</td></tr>
<tr><td>Sex</td><td>Male and female (relatively balanced)</td></tr>
<tr><td>Inclusion criteria</td><td>1. Age 50 years or older on the day of enrollment, regardless of gender, and able to provide legal identification.
2. Able to understand the trial procedures, voluntarily agree to participate in the study, and sign the "Informed Consent Form."
3. Female subjects are not pregnant or lactating. Female subjects with childbearing potential should take reliable contraceptive measures, and have no pregnancy and fertility plan within 8 months;
4. Axillary temperature ≤ 37.0°C/99.0°F on the day of enrollment.
5. Able to attend all scheduled follow-up visits and able to comply with protocol requirements.</td></tr>
</table>

Exclusion Criteria	1. History of herpes zoster within the past 5 years.<br>2. Previous vaccination with varicella vaccine or zoster vaccine (including use of registered products or participation in clinical trials for varicella or zoster vaccines).<br>3. Allergy to any component of the study vaccine: Previous allergy to any recombinant vaccine derived from CHO cells (such as recombinant hepatitis B vaccine [CHO cells]), polysorbates, or a history of severe allergic reactions* to any vaccination.<br>a) *Severe allergic reactions: Anaphylactic shock, allergic laryngeal edema, allergic purpura, thrombocytopenic purpura, localized allergic necrotic reaction (Arthus reaction), severe urticaria, etc.<br>4. Diagnosis of immune deficiency diseases (such as congenital or acquired immunodeficiency, HIV infection, etc.), or receiving immunosuppressive/cytotoxic treatment (e.g., chemotherapy, organ transplantation, or planned treatment during the clinical trial within 6 months before vaccination).<br>5. Subjects who received immunosuppressive therapy within 3 months before vaccination, or planning to receive such treatment within 1 month after full immunization (e.g., long-term use of systemic glucocorticoids for ≥ 14 days, dose ≥ 2 mg/kg/day or ≥ 20 mg/day prednisone or equivalence);<br>6. Receiving an inactivated vaccine, recombinant vaccine, or mRNA vaccine within 14 days or any live vaccine within 28 days prior to vaccination;<br>7. Subjects with acute illness or in the acute phase of a chronic disease within 3 days prior to vaccination.<br>8. Asplenia or splenectomy.<br>9. Subjects who receive blood products or immunoglobulin therapy within 3 months before vaccination, or planning to use such products within 2 months after vaccination.<br>10. Participating in other clinical studies of investigational or un-registered products (drugs, vaccines or devices, etc.), or planning to participate in other clinical studies before the end of this clinical study.<br>11. Any significant underlying medical condition (e.g., life-threatening diseases that may limit survival to less than 4 years) or other conditions that the investigator believes may interfere with the completion of the study.
Postponement Criteria for the Second Vaccination	The study intervention (second dose of vaccine) should be suspended (or delayed) until the situation resolves in the following cases:<br>1. Axillary temperature > 37.0°C/99.0°F ;<br>2. Acute illness within 3 days prior to vaccination (e.g., acute upper respiratory infection with symptoms such as fever, cough, sore throat, difficulty breathing) or during an acute stage of a chronic disease, or use of antipyretics, analgesics, or antihistamines;<br>3. Insufficient interval between vaccinations (receiving an inactivated vaccine, recombinant vaccine, or mRNA vaccine within 14 days prior to vaccination, or any live attenuated vaccine within 28 days prior to vaccination);<br>4. Any Grade 3 or higher adverse event on the day of vaccination;

	5. Other circumstances where the investigator considers vaccination should be delayed.
Exclusion Criteria for the Second Dose	The investigator will terminate the administration of the second dose of the study vaccine if any of the following occur:<br>1. Any serious adverse event (SAE) related to the vaccination;<br>2. Severe allergic reaction following vaccination;<br>3. Suspected varicella-zoster virus (VZV) infection after the first dose of immunization;<br>4. Discovery of any new condition that meets the "exclusion criteria for the first dose";<br>5. Any other reason, as determined by the investigator, that requires discontinuation of the study vaccine administration.
Criteria for Subject Withdrawal from Study	A subject will be withdrawn from the study if any of the following conditions occur:<br>1. Development of intolerable severe adverse events or any condition that makes the subject unsuitable to continue participation in the study.<br>2. Occurrence of a severe allergic reaction related to the experimental vaccine, and the investigator assesses that the subject is no longer suitable to continue in the study.<br>3. The subject voluntarily withdraws from the study.<br>4. Poor compliance, failure to adhere to the study protocol, or loss to follow-up.<br>5. If an enrolled subject develops a condition during the study that makes continued participation unsuitable, and the investigator assesses that the risks of participation outweigh the potential benefits, the investigator may decide to withdraw the subject from the study.
Criteria for suspension and termination of the study	**Criteria for study suspension:**<br>1. New data regarding the study vaccine obtained from this study or any other study, or management reasons suggesting that the risks outweigh the benefits, prompting the sponsor, investigator, and/or ethics committee to recommend a suspension;<br>2. The occurrence of a serious adverse event that is potentially life-threatening or leads to the subject death, which may be related to the vaccination;<br>3. If ≥ 15% of subjects experience Grade 3 or higher adverse events during the study, these events do not resolve to below Grade 3 within 48 hours.<br>If the study is suspended, the ethics committee (IRB) and provincial or national drug regulatory authorities or relevant departments must be notified. An expert meeting will be conducted to assess safety, and a blinded analysis may be conducted if necessary to determine whether the study should continue. The study may resume once safety, protocol compliance, and data quality issues are resolved and the requirements of the sponsor, ethics committee, and/or national regulatory authorities are met.<br>**Criteria for study termination:**<br>(1) Reaching the study endpoints;<br>(2) Any subject experiencing a life-threatening or fatal serious adverse event

	related to the study vaccine during the study;<br>(3) The study meets the criteria for suspension (⩾15% of subjects experience ⩾ Grade 3 adverse events, and these do not resolve to below Grade 3 within 48 hours), and it is determined that these events are related to the study vaccine.<br>If the study is terminated early, the sponsor will immediately notify the investigator, clinical trial institution, ethics committee, and regulatory authorities of the reason for termination in accordance with the relevant regulatory requirements. The investigator should report to the clinical trial institution and IRB, providing a detailed written explanation.<br>Regardless of the reason for suspension or termination, the investigator must immediately inform the subjects and ensure appropriate follow-up is conducted.
**Study period**	Duration of participation for each subject: From the first visit to the final visit, each subject is expected to participate in the study for approximately 7 to 8 months.

Schedule of Activities

Visit	V1	V2	V3	V4	V5	V6	V7	V8
	D0	D7	D30	D37	D60	D67	D90	6 months after full immunization
**Time window (day)**	/	+3	-2/+7	+3	-2+7	+3	-2+7	-7+15
Signed informed consent	■							
Demographic and other basic data	■							
Medical history	■							
Physical examination[1]	■		●		★			
Inclusion criteria/Exclusion criteria	■		●		★			
Pregnancy test[2]	■		●		★			
Axillary temperature	■		●		★			
Randomization	■							
Blood Collection[3]	■				●		★	
Vaccination	■		●		★			
30 minutes observation	■		●		★			
Dispense and train Diary Card	■		●		★			
Review and collect Diary Card		■		●		★		
Dispense Contact Card		■		●		★		

Review and collect Contact Card			■		●		★	
Collection of Concomitant Medication	■	■	■	●	■	★	★	
Collection of Pregnancy Events	■	■	■	●	■	★	★	■
Serious Adverse Events	■	■	■	●	■	★	★	■

.

Note: ■: All subjects; ●: Experimental group subjects; ★: Control group subjects.

1.The physical examination includes height, weight, skin examination, blood pressure, etc. Height and weight may not be measured before the second dose.

2.Before each dose, female subjects of childbearing potential must undergo a urine pregnancy test, and the test must be negative in order to continue the study.

3.All subjects will have blood drawn before vaccination and 30 days after full immunization.

1. Introduction

1.1 Background

Varicella-zoster virus (VZV) causes two clinically distinct diseases. Primary infection with VZV results in varicella, which is characterized by generalized conditions and widely scattered rashes and small blisters. In general, it occurs in infants or primary school-age children. After initial varicella infection in infant and young child recovers, the virus establishes latency in the spinal ganglia and other parts of the body. When latent VZV is reactivated, herpes zoster (HZ) occurs, usually manifested as local rash or blisters, accompanied by severe pain.

Typical HZ presents with rash and blisters, usually lasting for 2 to 4 weeks. It is often accompanied by intense pain being described as burning, stabbing pain. In some patients, even gentle touching may cause severe pain in the affected area, which is often referred to as allodynia. The HZ-related pain may be severe, with or without itching, and sometimes is too severe to tolerate.

The most common complication of HZ is post-herpetic neuralgia (PHN). PHN is the pain that persists after the rash/blister resolves. Affected patients often report persistent burning, throbbing, intermittent intense or shock-like pain, or indescribable allodynia. Age is the main risk factor for PHN. PHN may last for several months, and approximately 70% to 80% will resolve within 1 year, despite some may torture patients for many years [Dworkin, 2007]. Other risk factors may include severe HZ rash, which may be a precursor of PHN.

Other complications of HZ include disorders of the eyes, nerves, skin, and viscera, which can lead to severe disabilities. The most common ocular complications of HZ are keratitis and uveitis. Other ophthalmologic complications include ptosis, scleritis, retinitis, secondary glaucoma, and cataracts [Schmader, 2008; Carter, 2008]. Neurological complications of HZ include myelitis, motor neuropathy, brain, and spinal cord ischemic infarction, aneurysms, subarachnoid hemorrhage, and intracerebral hemorrhage [Gilden, 2009; Schmader, 2008].

The seroprevalence of VZV-IgG exceeds 90% in most adult populations, therefore, substantial individuals are at risk of developing HZ. Age is considered as the most common risk factor for shingles, and the incidence is relatively stable until the age of 40 years, with 2-3 in 1000 per year, and then gradually increases with age, with 5 in 1000 per year at the age of 50-59, and 10 in 1000 per year at ≥ 60 years [CDC, 2008; Oxman, 2005]. Although most data on the incidence of shingles comes from the United States (the Americas) and Europe, the available data indicate that the incidence of shingles is similar in other parts of the world (including Japan, South Korea, Australia and Latin America, mainland China).

About more than half of HZ cases occur in adults over 60, and >85 elders have a 50% chance of having shingles in their lives [Oxman, 2005]. Older patients also have the

highest risk of developing PHN, with those aged 70 years and older representing 18-50% of PHN [Oxman, 2005; Scott, 2006]. Patients with impaired cell-mediated immunity (CMI) due to disease, medication, medical intervention, or advanced age are at increased risk for HZ [Cohen, 2007].

Individuals who have received the attenuated VZV strain (OKA strain) vaccine or who have acquired protective immunity after natural VZV infection. VZV-specific antibodies (Abs) help control viral spread in immunocompromised individuals, potentially contributing to limiting the severity of herpes zoster (HZ). Additionally, in the efficacy study of Zostavax™, a correlation was observed between the concentration of anti-VZV antibodies generated after vaccination and protection against HZ [Levin, 2008]. The Oka strain live attenuated vaccine was approved by the U.S. FDA in 1995, followed by approval from regulatory bodies such as the National Medical Products Administration of China and the European Union for pediatric vaccination to prevent wild-type varicella infection in children. The high-dose OKA strain live attenuated vaccine, Zostavax™ (19,400 PFU/dose), was first approved by the U.S. FDA in 2006 for the prevention of herpes zoster in individuals aged 60 and above. In 2011, the indication was expanded to include those aged 50 and above. A single dose (0.65 mL, 19,400 PFU/dose) is administered subcutaneously in the deltoid region of the upper arm. Occasional adverse reactions, such as headache and local injection site reactions, may occur. Large-scale, multicenter clinical trials have verified that vaccination reduced the incidence of herpes zoster by 69.8% in immunocompetent individuals aged 50–59. This product was discontinued in the U.S. market in November 2020.

Shingrix™ is a recombinant subunit zoster vaccine developed by GlaxoSmithKline Biologicals (GSK Biologicals), which consists of VZV glycoprotein E (gE) and a special adjuvant. VZV gE was selected as the subunit vaccine antigen because gE is the target of the host immune response [Cohen, 2007]. gE plays an important role in viral infection and is the antigen to induce neutralizing antibodies against VZV. The vaccine does not contain live virus and has been shown to be safe in all populations, including immunocompromised population. The adjuvant system AS01B, used in combination with the VZV gE antigen (molecular weight approximately 70 KDa), was developed by GSK Biologicals. It is an oily adjuvant composed of the immunostimulant MPL (3-O-deacyl-4'-monophosphoryl lipid A), QS21, liposomes, etc. MPL is chemically detoxified LPA derived from Gram-negative bacteria Salmonella Minnesota. QS-21 is a saponin molecule (triterpene glycoside) extracted and purified from the bark of Quillaja saponaria Molina. It also contains phosphatidylcholine and cholesterol. Vaccines with this oil-based adjuvant can produce high-titer specific antibodies after vaccination, but the incidence of local side effects is much higher than other vaccines, and local pain may last for 2 to3 weeks.

Shingrix™ in immunocompetent adults≥ 50 years reduced the incidence of herpes zoster and PHN by 97.2% and 91.2%, respectively, and by 89.8% and 88.8% in elders≥

70 years, which showed better efficacy than live attenuated vaccine Zostavax™. However, the ultra-high incidence of side effects limits its promising application. In August 2020, this vaccine was officially launched in mainland China for the prevention of herpes zoster in individuals aged 50 and older. The vaccine is administered in a 50μg dose via intramuscular injection into the deltoid muscle, with two doses given 1 to 6 months apart.

Currently, there are two types of zoster vaccines available on the market in China. One is the imported Shingrix™, and the other is the domestically produced live attenuated varicella-zoster virus vaccine, Ganwei®, developed by Changchun BCHT Biotechnology Co., Ltd., which was approved for sale on January 31, 2023. Ganwei® is approved for adults aged 40 and older and requires only a single dose. Given the increasing incidence of herpes zoster among younger individuals, the rising morbidity rate, the large potential patient population in China, and the limited availability of zoster vaccines, the launch and application of zoster vaccines have become even more urgent.

The recombinant zoster vaccine (code: LZ901 vaccine) used in this clinical study is developed by Beijing Luzhu Biotechnology Co., Ltd. The active ingredient is varicella-zoster virus glycoprotein E Fc fusion protein expressed in CHO cells by gene recombination technology. The cloning and screening process of the LZ901 cell line has been carried out in chemically defined media without serum or other animal-derived components, any source of contamination with adventitious agents. The cryopreservation media used in the establishment of cell banks at all levels does not contain any blood or animal-derived proteins or protein hydrolysates. The culture medium and feed liquid used in the cell culture process are chemically defined. After the purification process of affinity chromatography, formaldehyde inactivation, anion exchange, molecular sieve chromatography, and others, the purified product is added with vaccine-specific stabilizer free of protein and proteolytic solution. After filtration through 0.2-micron sterilizing filter membrane, the resulting LZ901 bulk is appropriately diluted and adsorbed to aluminum hydroxide adjuvant to formulate a recombinant zoster vaccine. Each vial contains 0.5 ml, with a single human dose of 0.5 ml, containing 100 μg of LZ901 protein. The residual DNA content in each dose of the vaccine is less than 1 pg (the Pharmacopoeia of China specifies a maximum of 100 pg), ensuring the safety of the vaccine to the greatest extent possible. This vaccine is primarily used for the prevention of herpes zoster and its complications caused by the varicella-zoster virus.

The LZ901 vaccine is a tetramer of varicella-zoster virus glycoprotein E (gE) and Fc fusion protein with a molecular weight of approximately 400 KDa. It is rich in O-glycosylation and N-glycosylation, with glycosylation types covering those have been detected in VZV infection in humans. The LZ901 molecule contains a structure that actively presents VZV gE to Fcγ receptors (FcγR) on the surfaces of dendritic cells and monocytes. This receptor-mediated endocytosis efficiently initiates an immune response

against VZV gE, generating high-titer antibodies and a robust cellular immune response. The results of Phase I and Phase II clinical trials (see data in section 4.2.3) show that the recombinant zoster vaccine is safe and well-tolerated in healthy individuals aged 50-70 years when administered with a 0 and 30-day immunization schedule via intramuscular injection into the deltoid muscle. Adverse reactions are limited to common vaccine-related side effects, with a lower incidence than the active control vaccine (Shingrix™). After full immunization, both anti-gE antibodies and anti-VZV antibodies were found to be at high levels, with a 100% seroconversion rate. Immunogenicity results were significantly superior to those of the placebo group, with the high-dose group showing more pronounced effects. In conclusion, the recombinant zoster vaccine is safe, well-tolerated, and immunogenic. The immunization schedule for this study is as follows: Experimental group: 100 μg/0.5 mL/dose of recombinant shingles vaccine, administered on days 0 and 30 (2 doses in total). Control group: 50 μg/0.5 mL/dose of recombinant shingles vaccine, administered on days 0 and 60 (2 doses in total).

1.2 Benefits and Risks

1.2.1 Benefits

Subjects may potentially benefit from receiving the study vaccine, specifically in preventing herpes zoster caused by VZV (Varicella Zoster Virus) infection.

Vaccination against herpes zoster is the most effective method of prevention, and currently, only two zoster vaccines are available on the market in China. The development and launch of our recombinant zoster vaccine holds significant social value and benefit for China large potential patient population. It will greatly alleviate the pressure caused by the severe shortage of zoster vaccines in the country, addressing the demand for zoster vaccines in China and contributing to the improvement of public health.

1.2.2 Risks

LZ901 Phase I clinical trial (40 subjects with study vaccine) risk summary: After receiving the experimental vaccine (both high and low doses), the occurrence of local and systemic adverse events was as follows: pain (13 cases), fatigue (4 cases), headache (4 cases), abdominal pain (2 cases), swelling (2 cases), pruritus (2 cases at the injection site), erythema (1 case), chills (1 case), myalgia (1 case), pharyngeal discomfort (1 case), maculopapular rash (1 case), pruritus (1 case at another site), and pruritic rash (1 case). These were mostly grade 1 adverse events (AEs), which resolved on their own without the need for treatment, and no grade 3 or higher AEs were observed.

Phase II clinical trial (300 subjects with study vaccine) risk summary: After receiving the experimental vaccine (both high and low doses), the occurrence of local and systemic adverse events was as follows: pain (41 cases), induration (5 cases), swelling (3 cases), erythema (9 cases), pruritus (12 cases), fatigue (18 cases), and headache (6

cases). In the high-dose group (100 μg/dose), the most common adverse event was pain at the injection site (17.45%). The severity of adverse events was as follows: grade 1 (26.85%), grade 2 (6.04%), and grade 3 (2.01%). These adverse events did not require treatment and resolved on their own. Overall, the safety of the vaccine was favorable.

Known Risks: Based on the results of Phase I and Phase II clinical trial and previous vaccine experience, intramuscular injection is an invasive procedure. Subjects may experience local adverse reactions at the injection site, such as pain, swelling, induration, erythema, and pruritus, as well as systemic reactions like fatigue, headache, fever, gastrointestinal discomfort (nausea, vomiting, diarrhea, abdominal pain), and, in rare cases, allergic reactions. Most of these adverse events are mild, do not require treatment, and resolve on their own.

Potential Risks: The LZ901 vaccine is a recombinant protein expressed in CHO cells, which may contain trace amounts of residual CHO cell proteins. This could potentially cause allergic reactions in a small number of individuals who have previously received the CHO cell-derived hepatitis B vaccine. The vaccine contains excipients such as polysorbate and aluminum hydroxide adjuvant, which may cause mild irritation to the muscles at the injection site, leading to slight pain. A few individuals may not achieve adequate immune protection and could still be at risk of contracting herpes zoster.

1.2.3 Summary of benefits and risks

This product is the first recombinant zoster vaccine in China to enter Phase III clinical trial, and the results of this study may support the vaccine approval for market release to meet the demand for zoster vaccines.

Subjects may gain immunity against related diseases through vaccination with the study vaccine, thereby preventing the occurrence of herpes zoster. Throughout the study, all subjects will receive the attention of the investigator. If any subject experiences intolerable adverse reactions, the investigator will promptly stop subsequent vaccinations or terminate the study and provide the necessary medical treatment until the event resolves, with full support from the sponsor.

The sponsor will closely monitor the risks of the study, maintain ongoing discussions with investigators about any adverse events occurring during the study, and promptly inform both investigators and subjects about the latest information regarding the experimental vaccine or similar vaccines. If any risks that may harm the subjects interests are identified, the study will be immediately suspended or terminated, and the relevant regulatory authorities will be notified.

2. Study objectives and endpoints

2.1 Study Objectives

2.1.1 Primary objective

To evaluate the immunogenicity of cellular immune response induced by recombinant zoster vaccine (CHO cell) in subjects aged 50 years and older, in comparison to the control vaccine.

2.1.2 Secondary objective

To evaluate the immunogenicity and safety of humoral immune responses induced by recombinant zoster vaccine (CHO cells) in subjects aged 50 years and older.

2.2 Study Endpoints

2.2.1 Primary endpoints

The proportion of specific CD4+/CD8+ T cells activating at least two cytokines (TNF-α, IFN-γ, IL-2, CD40L) 30 days after full immunization.

2.2.2 Secondary endpoints

Immunogenicity endpoints

1) Geometric Mean Concentration (GMC) and seroconversion rate of anti-gE antibodies 30 days after full immunization;
2) Geometric Mean Fold Increase (GMFI) in anti-gE antibodies 30 days after full immunization compared to baseline (Day 0).

Safety endpoints

1. Incidence of solicited adverse events (AE) within 0-7 days after each dose, including local (injection site) and systemic (non-injection site) reactions;
2. Incidence of unsolicited AEs within 0-30 days after each dose;
3. Incidence of AEs within 30 minutes after each dose;
4. Incidence of adverse events of special interest (AESIs) from the first dose through 30 days after full immunization;
5. Incidence of serious adverse events (SAEs) from the first dose through 6 months after full immunization.

3. Study design

3.1 Overall Design

The experimental group will receive two doses of the recombinant zoster vaccine (CHO cells) with an immunization schedule of 0 and 30 days. The control group will receive two doses of the Recombinant Zoster Vaccine (RZV, Shingrix^{TM}) with an immunization schedule of 0 and 60 days.

The number of subjects in each group is shown in the table below:

Table 3 Treatment Groups

Age	Experimental group	Control group	Total target enrollment
50 and older	150	150	300

3.1.1 Sample Collection

3.1.1.1 Blood sample collection

Blood samples will be collected from all subjects once before vaccination and once 30 days after full immunization, for a total of two blood samples. The ELISA method will be used to measure varicella-zoster virus (VZV) gE-specific antibodies, and flow cytometry will be used to assess VZV gE-specific CD4+/CD8+ T cell activation. The proportion of subjects activating at least two cytokines (TNF-α, IFN-γ, IL-2, CD40L) will be calculated to evaluate the vaccine humoral and cellular immune response. Detailed blood sample collection time points, volumes, and purposes are shown in the table below:

Visit Time	Test items and blood collection volume（ml）		
	Serum Antibody	Cellular Immune	Total
D0	4.0-5.0	15.0-20.0	19.0-25.0
30 days after full immunization	4.0-5.0	15.0-20.0	19.0-25.0

Note: Serum antibody testing is performed using procoagulant tubes for blood collection; cellular immunity indicators are tested using anticoagulant tubes for blood collection.

3.1.1.2 Collection of Biological Samples for Suspected Herpes Zoster Cases

For all suspected herpes zoster cases, skin lesion samples should be collected whenever possible and tested by qPCR. Methods for biological sample collection (any of the following three methods may be chosen):

a) Polyester Swab Method: Use a sterilized needle to puncture the vesicle, then vigorously rub the base of the ruptured blister with a sterile polyester swab to collect infected epithelial cells. Place the swab directly into a sample tube containing virus preservation solution.

b) Glass Slide Method: Use the edge of a glass slide to scrape the selected maculopapules, ensuring that the slide collects infected epithelial cells. Then, using the same sterile polyester swab, wipe the base of the ruptured lesion and further wipe the edge of the glass slide to obtain infected epithelial cells. Place the swab into a sample tube containing virus preservation solution.

c) Crust Method: Use a glass slide to scrape the crusts from the herpes lesions and place

the collected crust directly into a sample tube containing virus preservation solution.

3.1.1.3 Urine sample collection (pregnancy test)

Women of childbearing age (all women from menarche to menopause) should be tested with a urine pregnancy test before vaccination, which must be performed on the day of vaccination and before each dose. Urine samples do not need to be stored after testing.

3.1.2 Vaccination

The experimental group will receive two doses of the recombinant zoster vaccine (CHO cells) with an immunization schedule of 0 and 30 days. The control group will receive two doses of the Recombinant zoster vaccine (RZV, Shingrix^{TM}) with an immunization schedule of 0 and 60 days.

3.1.3 Follow-up visit

3.1.3.1 Immunogenicity observation

Blood samples will be collected from all subjects once before vaccination and once 30 days after full immunization, for a total of two blood samples. The ELISA method will be used to measure varicella-zoster virus (VZV) gE-specific antibodies, and flow cytometry will be used to assess VZV gE-specific CD4+/CD8+ T cell activation. The proportion of subjects activating at least two cytokines (TNF-α, IFN-γ, IL-2, CD40L) will be calculated to evaluate the vaccine humoral and cellular immune response.

3.1.3.2 Safety observation

Collect all AEs (adverse events) within 30 days after vaccination (including solicited AEs within 30 minutes, 0-7 days, and all unsolicited AEs within 0-30 days). Follow up on all SAEs (serious adverse events) and pregnancy-related events from the first dose of vaccination to 6 months after full immunization.

Solicited AEs include:

Injection site (local) AEs	Pain，pruritus，induration，swelling，rash，redness
Non-injection site (systemic) AEs	Fatigue, tiredness, fever, headache, insomnia, myalgia, skin and mucosal abnormalities, pain at non-injection sites , diarrhea, constipation, vomiting, nausea, acute allergic reactions, arthritis, arthralgia

Unsolicited AEs include: Any adverse event that is not a solicited AE or any solicited AE occurring outside the solicitation period (e.g., If the solicited AEs mentioned above occur more than 7 days after vaccination, they should be recorded as unsolicited AEs).

Collection of AESIs (Adverse Events of Special Interest): Suspected cases of herpes zoster within 30 days following the first dose of vaccination to full immunization.

3.1.4 Study period

Duration of participation for each subject: From the first visit to the last visit, each

subject is expected to participate in the study for about 7-8 months.
Duration of the study: From the first visit to the last visit, it is expected to be about 7 to 8 months.
Duration of visit: 6 months after full immunization.

3.2 Rationale for the Selection of the Study Design

3.2.1 Rationale for the Selection of the Control Vaccine

Shingrix™ is a Herpes Zoster subunit vaccine developed by GlaxoSmithKline Biologicals (GSK). The vaccine consists of the VZV glycoprotein E (gE) and a special adjuvant. Shingrix™ in immunocompetent adults ⩾ 50 years reduced the incidence of herpes zoster and PHN by 97.2% and 91.2%, respectively, and by 89.8% and 88.8% in elders ⩾ 70 years, which showed better efficacy than live attenuated vaccine Zostavax™. However, the ultra-high incidence of side effects limits its promising application. In August 2020, this vaccine was officially launched in mainland China for the prevention of herpes zoster in individuals aged 50 and older. The vaccine is administered in a 50μg dose via intramuscular injection into the deltoid muscle, with two doses given 1 to 6 months apart.
The herpes zoster subunit vaccine developed by GlaxoSmithKline Biologicals SA (CHO cells) (brand name: Shingrix™), which is already available on the market in China, has been selected as the active control to evaluate the immunogenicity and safety of the experimental vaccine. The experimental vaccine will follow a 0-30day immunization schedule with two doses, while the control vaccine will follow a 0-60day schedule with two doses. Both the experimental vaccine and the control vaccine will have a dose of 0.5 mL per administration.

3.2.2 Preclinical trial data

3.2.2.1 Comparison of Immunogenicity between LZ901 Vaccine and Shingrix™ Vaccine in BALB/c Mice.

Two weeks after the second immunization, the specific antibody titers (GMT) in mice induced by the LZ901 vaccine and Shingrix™ vaccine were 53.8 and 67.7 times higher, respectively, compared to three weeks after the first dose. The VZV gE GMT for LZ901 and Shingrix™ vaccines were 46,144 and 69,941 ($P > 0.05$), with no significant difference between the two, indicating that the humoral immune response levels induced by the LZ901 and Shingrix™ vaccines are comparable. After immunization with the LZ901 vaccine, mouse lymphocytes stimulated with VZV gE showed that 100% of CD4+ T cells expressed at least two activation markers, and 90% of CD8+ T cells expressed at least two activation markers. Among them, 90% of the gE-specific CD4+ T cells expressed IFN-γ, and 85% of the gE-specific CD8+ T cells expressed CD40L. After immunization with the Shingrix™ vaccine, mouse lymphocytes stimulated with

VZV gE showed that 70% of CD4+ T cells expressed at least two activation markers, and 85% of CD8+ T cells expressed at least two activation markers. Among them, 65% of the gE-specific CD4+ T cells expressed IFN-γ, and 85% of the gE-specific CD8+ T cells expressed CD40L. The expression of other cytokines induced by the LZ901 vaccine was slightly higher than that of the Shingrix™ vaccine. Both the LZ901 and Shingrix™ vaccines induced a Th1-type cell-mediated immune response characterized primarily by the secretion of IFN-γ.

3.2.2.2 Toxicity test of repeated intramuscular injection of LZ901 vaccine for 12 weeks in SD rats with 4 weeks of recovery period

LZ901 vaccine was administered via repeated intramuscular injections to SD rats at doses of 1 dose/rat and 3 doses/rat (50 μg/dose), with a dosing schedule of once every 3 weeks for a total of 12 weeks (5 doses in total). During the administration period, the main observations included local injection site irritation and immune response related increases in FIB and cytokines IL-2 and IL-6. No significant systemic toxicity reactions were observed, and the no observed adverse effect level (NOAEL) was determined to be 3 doses/rat (50 μg/dose). At both the 1 dose/rat and 3 doses/rat (50 μg/dose) levels, local injection site irritation was noted. LZ901 vaccination induced the production of certain levels of VZV gE-specific IgG antibodies and neutralizing antibodies in SD rats, with no signs of immunotoxicity.

3.2.2.3 Active systemic anaphylaxis test of LZ901 vaccine in guinea pigs

No allergic reactions were observed in guinea pigs sensitized by intramuscular injection at the doses of 0.2 and 1 dose/animal (50 μg/dose), and challenged by intravenous injection at the doses of 0.4 and 2 doses/animal (50 μg/dose), respectively.

3.2.2.4 Reproductive toxicity study of LZ901 vaccine

This study used a total of 336 SD rats (224 females and 112 males), which were randomly divided into 4 groups by gender (28 males/group; 56 females/group). The groups were as follows: negative control group (administered 0.9% sodium chloride injection), adjuvant control group (administered adjuvant control substance—recombinant zoster vaccine (CHO cells) placebo), low and high dose test groups (administered recombinant zoster vaccine (CHO cells) at doses of 1 dose (50 μg) per rat and 3 doses (150 μg) per rat). The vaccine was administered by intramuscular injection in the hind limb, with doses of 0.5 mL per rat and 1.5 mL per rat, respectively. Clinical observations were conducted throughout the study, including checks on the body weight, food intake, and reproductive abilities of the parent female and male rats. At gestation day (GD) 20, cesarean section was performed on female rats to inspect embryonic and fetal development, fetal appearance, bones, and internal organs. After birth, F1 offspring survival, body weight, and physical and reflex development were monitored. Additionally, serum levels of herpes zoster specific IgG antibodies and neutralizing

antibodies in both the GD20 pregnant females, fetal rats, and F1 offspring were measured.

Under the conditions of this study, LZ901 vaccine was administered by intramuscular injection to SD rats before mating, during pregnancy, and through lactation at doses of 1 dose per rat and 3 doses per rat. No significant adverse effects were observed on fertility, pregnancy, and lactation in the parental female and male rats, except for local swelling at the injection site in the 3dose group. No embryotoxicity, teratogenicity, or effects on F1 offspring growth and development were observed. Therefore, the No Observed Adverse Effect Level (NOAEL) for LZ901 vaccine in terms of reproductive toxicity, embryonic and fetal development, and offspring growth and development was determined to be 3 doses per rat. Furthermore, all dose groups of SD rats showed high levels of anti-VZV neutralizing antibodies. Offspring born through normal delivery exhibited higher levels of anti-VZV neutralizing antibodies compared to those born via cesarean section.

3.2.3 Phase I/II Clinical Trial Data

The recombinant zoster vaccine developed by our company has completed Phase I and Phase II clinical trial, evaluating its safety, tolerability, and immunogenicity in healthy individuals aged 50 to 70.

3.2.3.1 Phase I clinical trial

The Phase I clinical trial in healthy individuals aged 50-70 years was conducted with a 0 and 1month immunization schedule, administering one dose of the experimental vaccine or placebo to the deltoid muscle, and a 0 and 2month immunization schedule, administering one dose of the active control vaccine (Shingrix^{TM}) to the deltoid muscle. The trial results showed:

Adverse event rates in the high-dose group, low-dose group, placebo group, and active control group (Shingrix^{TM}) were 30.00%、50.00%、20.00% and 100.00%, respectively. The adverse event rate in both the experimental vaccine groups was similar to that in the placebo group, but significantly lower than in the active control group. The severity of adverse events was mainly Grade 1, requiring no treatment and resolving spontaneously.

Humoral Immunity: In both high-dose and low-dose groups, the seroconversion rate for anti-gE antibodies (ELISA) 1 month after full immunization was 100%, with antibody levels of 23.73 IU/mL and 24.84 IU/mL, respectively, representing a 16.88 and 34.04fold increase from baseline. The seroconversion rate for anti-VZV antibodies (FAMA) was also 100%, with geometric mean titers (GMT) of 512.00 and 194.01, respectively, reflecting a 36.76 and 13.93fold increase from baseline. The GMT for anti-VZV antibodies was significantly higher in the high-dose group compared to the low-dose group. There were no significant differences between the high-dose, low-dose groups, and the active control group in terms of the seroconversion rate, seroconversion

rate, or 2fold increase in anti-gE and anti-VZV antibodies.

Cellular Immunity: 30 days after full immunization, the proportion of gE-specific CD4+ and CD8+ T cells expressing two or more cytokines (IFN-γ, TNF-α, IL-2, and CD40L) was 85.00%、65.00%、20.00%、75.00%, and 70.00%、55.00%、25.00%、45.00%, and 45.00% in the high-dose, low-dose, placebo, and active control groups, respectively. Both the high-dose and low-dose experimental groups, as well as the active control group, showed significantly higher levels than the placebo group. There were no significant differences between the high-dose, low-dose experimental groups, and the active control group, although the high-dose test group exhibited a slight advantage.

3.2.3.2 Phase II clinical trial

A Phase II clinical trial was conducted in healthy individuals aged 50-70 years to evaluate the immunogenicity and safety of the vaccine. The trial involved administering one dose of the experimental vaccine (either 100μg per dose high-dose or 50μg per dose low-dose) or placebo to the deltoid muscle at 0 and 30 days, with 150 subjects in each group. The trial results showed:

- Subjects in both the low-dose and high-dose vaccine groups exhibited significantly higher humoral immune responses, including anti-gE and anti-VZV antibodies, compared to the placebo group.
- The seroconversion rates for anti-gE antibodies (ELISA) were 100% in both the high-dose and low-dose vaccine groups one month after the full immunization, and the seroconversion rates for anti-VZV antibodies (FAMA) were 100% in both groups. There were no significant differences between the high-dose and low-dose vaccine groups in terms of seropositivity, seroconversion rates, or the 2fold increase rate.
- One month after full immunization, the anti-gE antibody concentrations in the high-dose and low-dose vaccine groups were 24.19 IU/mL and 16.03 IU/mL, respectively, representing a 21.60fold and 17.42fold increase compared to pre-immunization levels. The geometric mean titer (GMT) for anti-VZV antibodies was 264.77 and 184.90, respectively, representing a 4.98fold and 3.85fold increase. The high-dose group showed significantly higher anti-gE antibody levels ($p<0.0001$) and anti-VZV antibody GMT ($p=0.0017$) compared to the low-dose group, suggesting a potential dose-response relationship.
- The incidence of solicited adverse reactions related to vaccination in the low-dose, high-dose, and placebo groups were 20.00%, 26.85%, and 11.26%, respectively. Both vaccine groups had a higher incidence of adverse reactions than the placebo group, with similar incidence rates between the low-dose and high-dose vaccine groups. The severity of adverse reactions was mostly grade 1, and the reactions were self-alleviating.

In conclusion, the recombinant zoster vaccine demonstrated acceptable safety and

tolerability. The high-dose group (100μg per dose) did not show a significant difference in safety compared to the low-dose group (50μg per dose); however, the high-dose group exhibited certain advantages in terms of anti-gE antibody levels and cellular immunity. Therefore, the immunization schedule for the Phase III clinical trial of LZ901 will select the 100μg per dose recombinant zoster vaccine administered at 0 and 30 days, with two doses in total.

3.3 Start and end of the study

The stud start is marked by the initiation meeting. The study will conclude when the conditions for the study completion analysis are met.

4. Study Population

4.1 The selection of the study population

This study is a randomized, partially blinded study, with plans to recruit approximately 300 subjects aged 50 years and older.

4.2 The selection basis for the study population

Since the LZ901-330 control vaccine is a Recombinant Zoster Vaccine (RZV, Shingrix^{TM}) developed by GlaxoSmithKline Biologicals SA, which is approved for use in adults aged 50 years and older, the recruitment age for both the experimental and control groups in the LZ901-330 study is set to 50 years and older.

4.3 Inclusion criteria

1) Age 50 years or older on the day of enrollment, regardless of gender, and able to provide legal identification.
2) Able to understand the trial procedures, voluntarily agree to participate in thes tudy, and sign the "Informed Consent Form."
3) Female subjects are not pregnant or lactating. Female subjects with childbearing potential should take reliable contraceptive measures, and have no pregnancy and fertility plan within 8 months;
4) Axillary temperature ≤ 37.0°C/99.0°F on the day of enrollment.
5) Able to attend all scheduled follow-up visits and able to comply with protocol requirements.

4.4 Exclusion criteria

1) History of herpes zoster within the past 5 years.
2) Previous vaccination with varicella vaccine or zoster vaccine (including use of registered products or participation in clinical trials for varicella or zoster vaccines).
3) Allergy to any component of the study vaccine: Previous allergy to any recombinant vaccine derived from CHO cells (such as recombinant hepatitis B vaccine [CHO cells]), polysorbates, or a history of severe allergic reactions* to any

vaccination.

a) *Severe allergic reactions: Anaphylactic shock, allergic laryngeal edema, allergic purpura, thrombocytopenic purpura, localized allergic necrotic reaction (Arthus reaction), severe urticaria, etc.

4) Diagnosis of immune deficiency diseases (such as congenital or acquired immunodeficiency, HIV infection, etc.), or receiving immunosuppressive/cytotoxic treatment (e.g., chemotherapy, organ transplantation, or planned treatment during the clinical trial within 6 months before vaccination).
5) Subjects who received immunosuppressive therapy within 3 months before vaccination, or planning to receive such treatment within 1 month after full immunization (e.g., long-term use of systemic glucocorticoids for ≥ 14 days, dose ≥ 2 mg/kg/day or ≥ 20 mg/day prednisone or equivalence);
6) Receiving an inactivated vaccine, recombinant vaccine, or mRNA vaccine within 14 days or any live vaccine within 28 days prior to vaccination;
7) Subjects with acute illness or in the acute phase of a chronic disease within 3 days prior to vaccination.
8) Asplenia or splenectomy.
9) Subjects who receive blood products or immunoglobulin therapy within 3 months before vaccination, or planning to use such products within 2 months after vaccination.
10) Participating in other clinical studies of investigational or un-registered products (drugs, vaccines or devices, etc.), or planning to participate in other clinical studies before the end of this clinical study.
11) Any significant underlying medical condition (e.g., life-threatening diseases that may limit survival to less than 4 years) or other conditions that the investigator believes may interfere with the completion of the study.

Exclusion Criteria for the Second Dose

The investigator will terminate the administration of the second dose of the study vaccine if any of the following occur:

1) Any serious adverse event (SAE) related to the vaccination;
2) Severe allergic reaction following vaccination;
3) Suspected varicella-zoster virus (VZV) infection after the first dose of vaccination;
4) Discovery of any new condition that meets the "exclusion criteria for the first dose";
5) Any other reason, as determined by the investigator, that requires discontinuation of the study vaccine administration.

4.5 Screening Failure

Screening failure refers to a situation where a subject has signed the informed consent form and agreed to participate in the study, but is not randomly assigned or included in the study. To ensure transparent reporting of screening failures, partial information

about the subjects, including demographic details and reasons for screening failure, will still be recorded in the database.

Subjects who do not meet the eligibility criteria for participation in the study prior to enrollment (when the inclusion and exclusion criteria specified in the protocol are temporarily not met) may undergo re-screening. During re-screening, the subject screening number should remain the same as the one assigned during the initial screening.

5. Study intervention

5.1 Study intervention procedure

5.1.1 Subject recruitment

The study will be conducted in accordance with all applicable regulatory requirements. It will also comply with the "Good Clinical Practice (GCP)" guidelines, all relevant subject privacy requirements, and the principles of the Declaration of Helsinki. Once the study sites are determined and ethical committee approval is obtained, before the study begins, the investigator or their authorized representative will issue recruitment notices to subjects who meet the inclusion criteria regarding age, vaccination history, and health status. These subjects will be contacted and registered, and invited to participate in the study.

5.1.2 Informed Consent

The informed consent process should be completed before any study procedures are conducted. Prior to enrollment, the investigator will provide the subject and/or their legal guardian with relevant information about the study. The subject and/or their legal guardian, along with the investigator, will sign the Informed Consent Form, in duplicate. One copy will be retained by the subject, and the other will be kept at the study site.

5.1.3 Screening

The investigator will inquire about the subject medical history, vaccination history, and other relevant information based on the "Inclusion and Exclusion Criteria." All subjects will undergo axillary temperature measurement and physical examination, including height, weight, skin examination, blood pressure, etc. Women of childbearing age will also undergo a urine pregnancy test. The investigator will record the screening information and demographic data (such as date of birth, gender, and ethnicity) in the "Vaccination and Follow-up Record Book."

5.1.4 Randomization of Subjects

Eligible subjects who pass screening will be assigned a study number in sequential order, which will be used to identify all procedures conducted after the subject is enrolled.

All subjects will be randomized through a centralized system in a 1:1 ratio to either the

experimental group or the control group. The centralized randomization system will assign the corresponding vaccine number based on the subject group allocation, and the subject will receive the vaccine corresponding to the assigned vaccine number.

5.1.5 Sample Collection

Subjects will have blood samples collected at two time points: prior to vaccination and 30 days after the full immunization. A total of two blood samples will be collected. The samples will be analyzed using the ELISA method to detect varicella-zoster virus (VZV) glycoprotein E (gE)-specific antibodies and flow cytometry will be used to assess the activation of VZV gE-specific CD4+/CD8+ T cells, analyzing the proportion of cells expressing at least two cytokines (TNF-α, IFN-γ, IL-2, CD40L). This will evaluate the humoral and cellular immune responses induced by the vaccine.

For suspected herpes zoster cases, samples should be collected from different vesicular/maculopapular lesions (three samples should be collected, using one of the three available collection methods).

Women of childbearing age (from menarche to pre-menopause) must undergo a urine pregnancy test prior to vaccination. The pregnancy test must be conducted on the day of vaccination and before each dose of the vaccine. There is no need to store urine samples after testing.

Detailed information on sample collection is provided in Section 3.1.1.

5.1.6 The first dose of vaccination

Subjects will receive the first dose of the vaccine on the day of enrollment, according to their assigned study number.

5.1.7 The second dose of vaccination

The experimental group will receive two dose recombinant zoster vaccine (CHO cells) developed by Beijing Luzhu Biotechnology Co., Ltd./Luzhu Biopharmaceuticals (Zhuhai) Co., Ltd., following a 0 and 30-day immunization schedule.

The control group will receive the two dose Recombinant Zoster Vaccine (RZV, Shingrix^{TM}) developed by GlaxoSmithKline Biologicals SA, following a 0 and 60-day immunization schedule.

Subjects in the experimental group will receive the second dose on Day 30, with a window period of -2/+7 days.

Subjects in the control group will receive the second dose on Day 60, with a window period of -2/+7 days.

If a subject has a relative or absolute contraindication, the vaccination schedule may be postponed or interrupted as described in Section 6.1.1.

Both the experimental and control vaccines will be administered via intramuscular

Group	Study Intervention	Dose	Vaccination Time		
			D0	D30	D60
experimental group	experimental vaccine	0.5mL/dose	√	√	_
control group	control vaccine	0.5mL/dose	√	_	√

injection into the deltoid muscle. The dose and schedule are as follows:

5.1.8 Medical Observation

After each dose of the study vaccine, the subject must be observed on-site for 30 minutes. The investigator will provide the subject with a Diary Card, thermometer, pen, and ruler, and guide them on how to measure and record their body temperature (axillary temperature) for 7 days after vaccination (the day of vaccination and the following 7 days), as well as record all adverse events. The subjects will be trained on how to use the thermometer and ruler, and how to monitor for adverse events and complete the Diary Card. A time will be scheduled for the collection of the Diary Card on day 7 after vaccination, along with the distribution of the Contact Card, and instructions on how to record all adverse events occurring between days 7-30 after vaccination. The Contact Card will be collected and reviewed 30 days after each dose of the vaccine.

The investigator will emphasize the researcher phone number on the Informed Consent Form and Diary Card, and instruct the subjects to immediately contact the investigator if they experience any symptoms or signs they consider severe or if they experience an event requiring hospitalization.

5.1.9 Safety Follow-up

5.1.9.1 Follow-up Time

(1) After each vaccine dose, the subject will be observed on-site for 30 minutes to monitor for any immediate adverse events.

(2) After each vaccine dose, subjects will be given a Diary Card containing information on potential adverse events. Subjects are instructed to measure their temperature daily and record all local and systemic adverse events from the day of vaccination to day 7, as per the instructions on the Diary Card.

(3) On day 7 after each vaccine dose, subjects will return the Diary Card, and the researcher will review and document any adverse reactions. Additionally, a Contact Card will be issued to the subjects to record adverse events from day 7 to day 30 after each dose.

(4) From the first vaccine dose to 6 months post-completion of the full immunization

schedule, adverse events (SAEs) and pregnancy-related events will be collected through active reporting by the subject and periodic telephone follow-up by the researcher.

(5) During each visit, the researcher will remind subjects that they can contact the researcher at any time through the phone numbers listed on the Diary Card, Contact Card, or Informed Consent Form.

5.1.9.2 Follow-up Content

The safety observation content includes all adverse events (AEs) and serious adverse events (SAEs) that occur during the study, such as: pain, pruritus，induration，swelling，rash， redness, fatigue, tiredness, fever, headache, myalgia, etc. It also includes the occurrence of all SAEs and pregnancy events from the first dose of vaccination until six months after the full immunization schedule is completed.

Solicited AEs include:

Injection site (local) AEs	Pain，pruritus，induration，swelling，rash，redness
Non-injection site (systemic) AEs	Fatigue, tiredness, fever, headache, insomnia, myalgia, skin and mucosal abnormalities, pain at non-injection sites , diarrhea, constipation, vomiting, nausea, acute allergic reactions, arthritis, arthralgia

Unsolicited AEs include: Any adverse event that is not a solicited AE or any solicited AE occurring outside the solicitation period (e.g., If the solicited AEs mentioned above occur more than 7 days after vaccination, they should be recorded as unsolicited AEs).

Serious Adverse Events (SAEs)

This study will collect all SAEs occurring from the first dose of vaccination until six months after the completion of the full immunization schedule.

Pregnancy Events

All pregnancy events occurring from the first dose of vaccination until six months after the full immunization schedule will be collected. Details on pregnancy reporting, follow-up, and assessment are provided in Section 7.5.

Specially Monitored Adverse Events

Suspected cases of herpes zoster occurring from the first dose of vaccination until 30 days after the completion of the full immunization schedule will be collected.

5.1.10 Concomitant Medications

During each visit or contact after vaccine administration and before the return of the Diary Card/Contact Card, the investigator must inquire whether the subject has taken any medications, received any treatments, or been vaccinated with any vaccines. All concomitant medications/vaccines (except for supplements, vitamins, and/or food additives) must be recorded on the Diary Card/Contact Card. The investigator should transcribe the concomitant medications into the electronic case report form (eCRF). The data management team will use the World Health Organization Drug Dictionary (WHO

DD) to code concomitant medications.

Concomitant Medications/Vaccines: These refer to all medications taken by the subject, excluding the study vaccine, from the first dose until 30 days after the last dose of the vaccine. This includes antibiotics, antivirals, antipyretic analgesics, antihistamines, biologics (vaccines), Chinese medicine (and traditional medicine), etc. (excluding vitamins or food supplements). The data management team will use the WHO DD to code the concomitant medications.

Medication Record: In order to understand the impact of medications used during the study on vaccine safety or to collect any potential AEs related to vaccination without omissions, the investigator must collect information about the medications taken by the subject during the observation period. The investigator should guide the subject and/or their legal guardian to record medical visits and medication use in the Diary Card/Contact Card, and the investigator must transcribe all medications taken within 30 days after vaccination into the eCRF.

5.1.10.1 Restricted Medications

At enrollment, the investigator should inquire about any medications the subject is currently using to ensure that the subject has not been taking antipyretics, analgesics, or antihistamines.

Preventive medications are those used to prevent potential solicited adverse events (AEs) that may occur during the solicitation period following vaccination. For example, if an antipyretic is taken to prevent the onset of fever when no fever is present, that antipyretic would be considered a preventive medication.

5.1.10.2 Allowed Medications

Allowed Vaccines: Vaccine use should follow the inclusion/exclusion criteria. Emergency vaccination (such as rabies or tetanus vaccination) is permitted and should not be restricted, but vaccine use must be recorded accurately as required. For vaccines included in the immunization schedule, live attenuated vaccines should be administered at least 28 days before the study vaccine, while inactivated vaccines, recombinant vaccines, or mRNA vaccines should be administered at least 14 days before the study vaccine. After receiving the study vaccine, other vaccines should be administered at least 14 days later.

Allowed Medications: During the study, if a subject experiences an adverse event, necessary medication treatment is allowed, and medication usage must be recorded accurately as required. In this study, as contraception is required for subjects, the use of contraceptives is permitted. However, any medication use must be accurately documented as required.

5.2 Basis for Intervention Selection

The recombinant zoster vaccine (code: LZ901 vaccine) used in this study is developed by Beijing Luzhu Biotechnology Co., Ltd./Luzhu Biopharmaceuticals (Zhuhai) Co., Ltd. It is a recombinant glycoprotein E fusion protein of varicella-zoster virus (VZV), expressed in CHO cells using genetic engineering techniques. The LZ901 cell line, carrying this gene, is cultured in a chemically defined medium, and the cell culture supernatant is harvested. Through a multi-step process including liquid chromatography purification, low pH incubation, and nanofiltration for virus inactivation/removal, a high-purity recombinant VZV glycoprotein E solution is obtained. This is then adsorbed onto an aluminum hydroxide adjuvant to prepare a vaccine with the appropriate concentration. The vaccine is provided in 0.5mL vials, with each human dose of 0.5mL containing 100μg of antigen. The recombinant zoster vaccine developed by the company is an aluminum-adjuvanted recombinant protein vaccine. Based on the results of Phase I and Phase II clinical trials (detailed in Section 3.2.3), the recombinant zoster vaccine has shown good safety in the 50-70year old healthy population with an immunization schedule of 0 and 30 days, administered intramuscularly in the deltoid. The adverse reactions were limited to common vaccine-related reactions, with an incidence rate lower than that of the control vaccine. Post-immunization, the levels of anti-gE antibodies and anti-VZV antibodies were high, with a 100% seroconversion rate, and the immunogenicity was significantly better than the placebo group, with the higher dose group showing more pronounced results. In conclusion, the recombinant zoster vaccine demonstrates acceptable safety and tolerability. The immunization schedule for this study: Experimental group: 100μg/0.5mL/dose of the recombinant zoster vaccine administered with a 0 and 30-day immunization schedule (2 doses in total). Control group: 50μg/0.5mL/dose of the recombinant zoster vaccine administered with a 0 and 60-day immunization schedule (2 doses in total).

5.3 Dosage and Administration

The study vaccine is administered at a dose of 0.5 mL per person, injected intramuscularly into the deltoid muscle. As with all vaccines, the vaccination site should be equipped with appropriate emergency medical measures and have medications such as epinephrine available for use in case of a severe allergic reaction. After vaccination, the subject should be observed on-site for 30 minutes.

5.4 Preparation, Handling, Storage, and Counting

5.4.1 Preparation of Study Intervention

The preparation and administration of both the study vaccine and the control vaccine will be carried out by authorized medical personnel who are not involved in the clinical assessment.The study vaccine is a ready-to-use liquid formulation intended for direct injection.

The control vaccine preparation follows the manufacturer instructions. The AS01B

adjuvant is mixed with the lyophilized varicella-zoster virus glycoprotein E (gE) antigen component from a single-dose vial to prepare the injectable suspension. After mixing, the final dose of the control vaccine is 0.5 mL per single administration.

5.4.1.1 Experimental vaccine

Common name: Recombinant zoster vaccine (CHO Cells)
Manufacturer: Luzhu Biopharmaceuticals (Zhuhai) Co., Ltd.
Lot Number: 20230201
Specification: 0.5 mL/vial
Expiration Date: 2025/02/12
Active Ingredient: Recombinant Varicella-Zoster Virus Glycoprotein E (gE) 100 µg
Adjuvant: Aluminum Hydroxide Adjuvant

5.4.1.2 Control vaccine

Common name: Recombinant Zoster Vaccine (RZV, Shingrix™)
Manufacturer: GlaxoSmithKline Biologicals SA
Lot Numbers: 3472D, X4937
Specification: 0.5 mL/vial
Active Ingredient: Recombinant Varicella-Zoster Virus Glycoprotein E (gE) 50 µg
AS01B Adjuvant System: Quillaja saponaria saponin QS-21 50 µg, 3-O-desacyl-4'-monophosphoryl lipid A (MPL) 50 µg, dioleoyl phosphatidylcholine 1 mg, cholesterol 0.25 mg
Other Components:
Sterile Powder for Injection (gE): Sucrose, Polysorbate 80, Disodium Phosphate Dihydrate, Dipotassium Phosphate
Suspension for Injection (AS01B Adjuvant System): Sodium Chloride, Disodium Hydrogen Phosphate Anhydrous, Potassium Dihydrogen Phosphate, Water for Injection
If the vaccine batch used in the trial differs from the one specified in the protocol, the responsible organization must explain and file the change with the Ethics Committee before the clinical trial begins (or as required by the IRB).

5.4.2 Blinding of Study Interventions

The study employs a partially blinded design, where vaccine blinding is carried out by the randomization statistician and other blinding personnel. According to the randomization code, pre-printed vaccine labels are applied to designated locations on each vaccine. The randomization statistician supervises the blinding process, guiding blinding personnel to label the vaccines according to the randomization code. Once blinding is complete, the randomization code should be sealed by the randomization statistician. The entire blinding process must be documented in writing. Blinding personnel are prohibited from participating in any other tasks related to this study and must not disclose the randomization code to any study personnel.

5.4.3 Handling and Storage of Study Interventions

The study vaccine should be stored and transported at 2-8°C, protected from light, and must not be frozen (depending on the specific characteristics of the vaccine). The storage temperature should be monitored twice daily (once in the morning and once in the afternoon) and recorded. If the temperature monitoring system includes automatic alerts, the temperature may be monitored and recorded based on the local circumstances during holidays. If the storage or transportation conditions fall outside the specified range, the site staff should immediately contact the study unit and the sponsor. The vaccine should not be used until approval is received from the sponsor.

5.4.4 Packaging and Labeling

The study vaccine outer packaging (small box) and inner packaging (vaccine vial) will have the same numbered labels, and one pre-printed adhesive label with the same number will be prepared for placement on the subject vaccination and follow-up record book. The labels will contain the following information:

1) Outer Packaging Label (Large Box): Protocol number, product name, specification, lot number, expiration date, storage temperature, study sponsor, and the label "For Clinical Trial Use Only" should be printed on the large box.
2) Small Box Vaccine Outer Packaging Label: Protocol number, product name, specification, vaccine lot number, expiration date, and vaccine number.
3) Vaccine Vial Label: The study vaccine name, lot number, expiration date, manufacturer, and the label "For Clinical Trial Use Only." A label containing the protocol number, product name, and vaccine number should be placed over the original label on the vaccine vial.
4) Adhesive Label (for the vaccination and follow-up record book): Protocol number, vaccine number.

After the vaccine is activated, the subject initials should be written on both the outer packaging label of the individual vaccine vial and the adhesive label (to be placed in the vaccination and follow-up record book). The administering personnel should verify the details before administering the corresponding vaccine based on the assigned number.

5.4.5 Inventory of Study Interventions

All study drugs shipped to the study site should be accompanied by a quality control report, shipping list, and a receipt confirmation form for site personnel to use. The shipping of study drugs must have complete written documentation, which typically includes the name or code of the study drug, dosage form, specification, lot number or drug code, quantity, expiration date, sponsor, manufacturing entity, packaging type, storage requirements, recipient institution and address, contact information, shipping date, transportation method, and temperature monitoring measures during transit.

Returned study drugs should not be reused in the study. Only after confirming the balance of the quantities of distributed, used, and returned study vaccines can unused

and returned study vaccines be destroyed. Destruction must be fully documented, including at least the reason for destruction, date of destruction, relevant lot numbers and/or drug codes, actual quantity destroyed, person responsible for destruction, and supervisor. Destruction records are retained by the sponsor.

5.4.6 Reserve vaccine

Sixty reserve vaccine doses will be prepared, with 30 doses each of the experimental vaccine and the active control vaccine. If a study vaccine is found to be damaged during the study, the investigator can obtain the reserve vaccine number for the corresponding group through the randomization system and administer the reserve vaccine labeled with the appropriate vaccine number. (The investigator only sees the randomization number; the true group assignments for the study and reserve vaccines are pre-loaded into the system and remain blinded throughout the study. Additional reserve doses may be added if the existing stock is insufficient.)

5.5 Subject Assignment, Randomization, and Blinding

5.5.1 Randomization of Subjects

Subject randomization occurs after screening. Eligible subjects are assigned a study number in sequential order, which is used to identify all procedures after enrollment. A total of 300 subjects aged 50 and above will be randomly assigned to the experimental or control group in a 1:1 ratio. The central randomization system matches each subject assigned group with a corresponding vaccine number, and subjects receive the vaccine according to this number. For further details, see Section 3.1.

5.5.2 Randomization

This study uses block randomization to allocate subjects to the experimental and control groups in a 1:1 ratio. The central randomization system assigns each subject group with a corresponding vaccine number. Researchers strictly follow the assigned vaccine number to obtain and administer the study vaccine.

5.5.3 Blinding and Unblinding

5.5.3.1 Blinding

This study adopts a partially blinded design. Vaccine blinding is conducted by the randomization statistician along with other blinding personnel. Pre-printed vaccine labels are affixed to each designated vaccine position according to the randomization code. The randomization statistician oversees the blinding process, guiding blinding personnel to label the vaccines according to the randomization code. Once blinding is complete, the randomization code should be sealed by the randomization statistician. The entire blinding process must be documented in writing. Blinding personnel are prohibited from participating in any other activities related to this study and must not

disclose the randomization code to any study personnel. Preparation and administration of the experimental/control vaccine will be handled by authorized medical staff who are not involved in the clinical evaluation of subjects.

5.5.3.2 Unblinding Procedures

In this study, unblinding is required before the administration of the second dose, as the dosing intervals differ between the experimental group (30 days) and the control group (60 days). The unblinding process must be initiated by the principal investigator, who submits an unblinding request to the sponsor, and a record of the unblinding must be maintained.

In the event of group adverse events or study interruption for any reason, early unblinding may be conducted with the mutual agreement of the sponsor and the research team.

5.5.3.3 Emergency Unblinding

During the study, individual emergency unblinding may only be conducted if a serious adverse event (SAE) occurs, the subject requires emergency rescue or treatment, and the product information is critical for the subject clinical management. In such cases, a researcher with unblinding authority at the study site should access the Clinflash IRT system to document the reason for unblinding and initiate emergency unblinding. The researcher must report the unblinding within 24 hours to the principal investigator or lead sub-investigator, promptly notify the sponsor and the sponsor designated CRO, and provide a written explanation to the sponsor.

The study will be terminated for the subject with the specific study number (while safety follow-up will continue), and the reason for termination should be documented in the case report form.

The investigator or any other physician managing the subject should contact the sponsor to discuss the necessity of emergency unblinding.

An emergency unblinding request should only be made if there is a medical emergency or serious condition where the study vaccine information is crucial to the subject clinical care or welfare.

The sponsor will permit unblinding only if the investigator responsible for the subject believes that the medical event cannot be managed without knowing the identity of the study vaccine.

5.6 Study Intervention Compliance

To ensure and document vaccination information and compliance with study interventions, subjects are required to remain on-site for 30 minutes after each dose of the study vaccine. The researcher provides each subject with a Diary Card, thermometer, and ruler, instructing them to measure and record their body temperature (axillary temperature) and note any adverse events for 7 days following vaccination. Subjects are trained on how to use the thermometer and ruler, how to observe adverse events, and

how to complete the Diary Card. The Diary Card is collected on day 7 post-vaccination, at which time subjects are also given a Contact Card for recording all adverse events occurring from day 7 to day 30 post-vaccination. The Contact Card is collected and reviewed 30 days after each dose.

6. Discontinuation of Study Intervention and Subject Withdrawal from the Study

6.1 Discontinuation of Study Intervention

6.1.1 Postponement Criteria for the Second Vaccination

The study intervention (second dose of vaccine) should be temporarily suspended (or delayed) until the situation resolves in the following cases:

1) Axillary temperature > 37.0°C/99.0°F;
2) Acute illness within 3 days prior to vaccination (e.g., acute upper respiratory infection with symptoms such as fever, cough, sore throat, difficulty breathing) or during an acute stage of a chronic disease, or use of antipyretics, analgesics, or antihistamines;
3) Insufficient interval between vaccinations (receiving an inactivated vaccine, recombinant vaccine, or mRNA vaccine within 14 days prior to vaccination, or any live attenuated vaccine within 28 days prior to vaccination);
4) Any Grade 3 or higher adverse event on the day of vaccination;
5) Other circumstances where the investigator considers vaccination should be delayed.

6.1.2 Exclusion Criteria for the Second Dose

The investigator will terminate the administration of the second dose of the study vaccine if any of the following occur:

1) Any serious adverse event (SAE) related to the vaccination;
2) Severe allergic reaction following vaccination;
3) Suspected varicella-zoster virus (VZV) infection after the first dose of vaccination;
4) Discovery of any new condition that meets the "exclusion criteria for the first dose";
5) Any other reason, as determined by the investigator, that requires discontinuation of the study vaccine administration.

6.2 Subject Withdrawal from Study

6.2.1 Criteria for Subject Withdrawal from Study

A subject will be withdrawn from the study if any of the following conditions occur:

1) Development of intolerable severe adverse events or any condition that makes the subject unsuitable to continue participation in the study.
2) Occurrence of a severe allergic reaction related to the experimental vaccine, and the investigator assesses that the subject is no longer suitable to continue in the study.
3) The subject voluntarily withdraws from the study.

4) Poor compliance, failure to adhere to the study protocol, or loss to follow-up.
5) If an enrolled subject develops a condition during the study that makes continued participation unsuitable, and the investigator assesses that the risks of participation outweigh the potential benefits, the investigator may decide to withdraw the subject from the study.

6.2.2 Reasons for Subject Withdrawal

Subjects may discontinue their participation in the study at any time and for any reason. The investigator should inform subjects that they have the right to withdraw from the study at any time, and withdrawn subjects will not be replaced. Subjects who withdraw due to SAE/AE should be distinguished from those who withdraw for other reasons. The investigator will follow up with subjects who withdraw due to SAE/AE until the issue is resolved.

If no study procedures, follow-ups, or further information collection occur from the date of withdrawal/last contact, the subject is considered to have "withdrawn" from the study. All data collected up to the withdrawal date/last contact will be used for analysis. The eCRF should record information related to the withdrawal, specifying whether the decision to withdraw was made by the subject or the investigator and indicating which of the following reasons led to the withdrawal:

- Serious adverse event (SAE)
- Non-serious adverse event (AE)
- Protocol deviation
- Withdrawal of informed consent (not due to an adverse event)
- Relocation from the study site
- Loss to follow-up
- Death
- Other (with detailed explanation)

6.3 Discontinuation of Study Vaccine

A subject is considered to have discontinued the study vaccine if they do not receive the complete series of study vaccinations, meaning that from the date of discontinuation, they no longer receive any of the planned study vaccines. Subjects who discontinue the study vaccine are not necessarily withdrawn from the study. Depending on the circumstances, the investigator may arrange for them to continue completing other study procedures or visits as specified in the protocol (e.g., for safety or immunogenicity assessments).

Information related to early discontinuation of the study vaccine will be recorded in the eCRF. The investigator will document whether the decision to stop further vaccination/treatment was made by the subject or the investigator, along with the specific reason, which may include:

- Serious adverse event (SAE).
- Non-serious adverse event.
- Other (with specific details).

6.4 Loss to Follow-Up

Loss to follow-up refers to a situation where a subject, for any reason, is unable to continue through to the final follow-up visit as required by the study protocol. The investigator will attempt to contact subjects who do not return for scheduled visits or follow-up.

6.5 Criteria for study suspension

1) New data regarding the study vaccine obtained from this study or any other study, or management reasons suggesting that the risks outweigh the benefits, prompting the sponsor, investigator, and/or ethics committee to recommend a suspension;
2) The occurrence of a serious adverse event that is potentially life-threatening or leads to the subject death, which may be related to the vaccination;
3) If ≥ 15% of subjects experience Grade 3 or higher adverse events during the study, and these events do not resolve to below Grade 3 within 48 hours.

If the study is suspended, the ethics committee (IRB) and provincial or national drug regulatory authorities or relevant departments must be notified. An expert meeting will be convened to assess safety, and a blinded analysis may be conducted if necessary to determine whether the study should continue. The study may resume once safety, protocol compliance, and data quality issues are resolved and the requirements of the sponsor, ethics committee, and/or national regulatory authorities are met.

6.6 Criteria for study termination

(1) Reaching the study endpoints;
(2) Any subject experiencing a life-threatening or fatal serious adverse event related to the study vaccine during the study;
(3) The study meets the criteria for suspension (⩾15% of subjects experience ⩾ Grade 3 adverse events, and these do not resolve to below Grade 3 within 48 hours), and it is determined that these events are related to the study vaccine.

If the study is terminated early, the sponsor will immediately notify the investigator, clinical trial institution, ethics committee, and regulatory authorities of the reason for termination in accordance with the relevant regulatory requirements. The investigator should report to the clinical trial institution and IRB, providing a detailed written explanation.

Regardless of the reason for suspension or termination, the investigator must immediately inform the subjects and ensure appropriate follow-up is conducted.

7. Study Evaluation and Procedures

7.1 Screening Evaluation and Procedures

The study will be conducted in accordance with all applicable regulatory requirements. Additionally, the study will comply with the Good Clinical Practice (GCP) guidelines, all relevant subject privacy requirements, and the principles of the Declaration of Helsinki. After identifying the study sites and obtaining ethics committee approval, the investigator or their authorized representative will distribute recruitment notices to subjects who meet the age, vaccination history, and health status requirements before the study begins, and contact and register these individuals, inviting them to participate in the study.

The informed consent process must be completed before any study procedures are conducted. Before enrollment, the investigator will inform the subjects about the study, and both the subject and the investigator will sign two copies of the Informed Consent Form, one for the subject and one to be retained at the study site.

The investigator will assess eligibility by conducting an inquiry based on the "Inclusion and Exclusion Criteria," obtaining information on the subject medical and vaccination history, and performing axillary temperature checks and physical examinations for all subjects, including measurements of height, weight, skin condition, and blood pressure. Women of childbearing age will undergo a urine pregnancy test. Screening information and demographic data (such as birth date, gender, and ethnicity) will be recorded in the Vaccination and Follow-up Record Book.

7.2 Immunogenicity Evaluation and Procedures

All subjects will have blood samples collected twice: once before vaccination and once 30 days after the full immunization. ELISA will be used to detect varicella-zoster virus (VZV) gE-specific antibodies, and flow cytometry will be utilized to assess the activation of VZV gE-specific CD4+/CD8+ T cells. The proportion of subjects with at least two cytokines (TNF-α, IFN-γ, IL-2, CD40L) activated will be calculated to evaluate both humoral and cellular immune responses induced by the vaccine.

7.3 Safety Evaluation and Procedures

7.3.1 Physical Examination

A physical examination will be completed during the screening period.

7.3.2 Temperature Check

Temperature checks will be conducted during the screening period and before vaccination.

7.3.3 Safety Assessment

The safety analysis cohort will include all subjects who meet the following criteria:

- Have received at least one dose of the study vaccine or control vaccine according to randomization;
- Have sufficient data for safety analysis (at least one dose and follow-up for safety);
- Have not received other prohibited medications or vaccines per protocol.For detailed information on the safety follow-up time and content, please refer to Section 5.1.9.

7.4 Adverse events and serious adverse events

7.4.1 Definitions of AE and SAE

Adverse Event (AE): Refers to any undesirable medical occurrence experienced by a subject after receiving the study drug. This can include symptoms, signs, diseases, or abnormal laboratory results, but it does not necessarily have a causal relationship with the study drug.

Serious Adverse Event (SAE): Refers to any adverse medical occurrence experienced by a subject after receiving the study drug, meeting any of the following criteria:

1) Results in death;
2) Is life-threatening, meaning the subject was at immediate risk of death at the time of the adverse event (not to be confused with potential risks of death if the condition worsens);
3) Results in hospitalization or prolongation of an existing hospitalization;
4) Results in persistent or significant disability/incapacity;
5) Leads to a congenital anomaly or birth defect;
6) Other important medical events.

Medical and scientific judgment should be used to decide whether to expedite the report of other situations. Events that may not immediately threaten life, result in death, or require hospitalization but necessitate medical intervention to prevent one of the above outcomes are also generally considered serious.

For further details on AE and SAE definitions, refer to Appendices 11.1 and 11.2.

7.4.2 Identification and Documentation of AEs and SAEs

During subject training, the importance of promptly reporting adverse events (AEs) is emphasized, and researchers should remain vigilant in identifying, investigating, and managing these events as they occur. For both solicited and unsolicited AEs experienced by subjects, the researcher should inquire whether the subject required hospitalization, outpatient treatment, or took any medications independently, and these details should be documented. Throughout the study, researchers should carefully observe the subject response to the vaccine without leading questions and encourage truthful reporting.

In the case of a serious adverse event (SAE), the investigator is responsible for reviewing all relevant documentation (e.g., hospital records, physician orders, lab reports, and diagnostic information). To clarify the nature and causality of the SAE, the

investigator may be asked by the sponsor to arrange clinical assessments/tests. If a subject is confirmed to have died during the study or follow-up period, a death certificate stating the cause of death should be obtained from the hospital.

Investigators should collect comprehensive case records as much as possible, but these should not replace study records. All information related to an SAE must be recorded in the original records, the eCRF, and the SAE report form. If medical records need to be disclosed for medical review, all personal identifiers should be redacted before release.

Investigators must establish an emergency plan for handling SAEs within the study and train all relevant personnel accordingly. Measures should be in place to promptly detect any clinically significant illnesses/events following vaccination and ensure that subjects receive appropriate care at designated hospitals in line with national regulations and current medical standards. The investigator should document the AE symptoms, onset time, severity, management, and outcome while analyzing its relationship to the study vaccine. Medications used to treat the AE should be recorded in the subject original records and eCRF.

For further details on assessing AE severity, SAE, and causality with vaccine administration, refer to Appendices 11.3 and 11.4.

7.4.3 Follow-up for AEs and SAEs

Subjects who experience adverse events (AEs) will be followed up until the event has resolved or improved, returned to baseline, or if the investigator determines that further follow-up is unnecessary, or if the subject is lost to follow-up. For AEs that lead to study termination or remain ongoing at the end of the study, the investigator should continue monitoring. Vaccine-related AEs should be followed until resolution, while unrelated events (such as illnesses) may be concluded once a physician diagnosis is obtained.

In case of disagreements or disputes during AE management, the investigator must cooperate with the sponsor to address the issue and assist the subject with medical evaluation if necessary. The sponsor has the obligation and responsibility to unconditionally ensure subject safety, providing humane care and compensation for subjects experiencing vaccine-related AEs during the study.

7.4.4 SAE Reporting

For any SAE occurring during the study, the investigator must inform the principal investigator and sponsor within 24 hours of awareness via phone, email, or online reporting. A complete written report on the Serious Adverse Event Report Form should be submitted, detailing the full description of the adverse reaction/event, onset time and type, duration, intensity, causality with vaccination, outcome, treatment (symptomatic), and other relevant clinical and laboratory data. After the initial SAE report, the investigator should continue to monitor the SAE, submitting follow-up reports with new or updated information, including any changes to the previous report and event

outcomes, focusing on unresolved/recovering SAEs until a definitive outcome is reached. For hospitalized subjects, the investigator should obtain the hospital records, which include the full progression and outcome of symptoms, all drug treatments, and detailed medical records. This information should be promptly reported as new or updated information.

The investigator should track the subject SAE outcome and aim to follow up until symptoms resolve, completing a summary report. For medically irreversible conditions, follow-up should continue until symptoms stabilize, return to pre-treatment levels, or when an alternative explanation is provided, completing the summary report.

In cases involving death, the investigator must report to the sponsor and ethics committee, providing an autopsy report (if available) and a final medical report.

7.4.5 SAE Regulatory Reporting Requirements

The investigator is responsible for promptly reporting SAEs to the sponsor (and to the ethics committee, if required) to enable the sponsor to fulfill their responsibilities. The sponsor must immediately analyze and assess all SAEs reported by the investigator, including evaluating their severity, causality with the study vaccine, and whether the event was anticipated. Any SAE identified by the sponsor as a suspected unexpected serious adverse reaction (SUSAR) must be promptly reported to all participating investigators, study sites, ethics committees, and the National Medical Products Administration's Center for Drug Evaluation (CDE) within seven days, with follow-up information provided within the subsequent eight days. If the sponsor and investigator cannot reach an agreement on the causality assessment between the adverse event and the vaccine, a prompt report should still be made if either party deems it impossible to rule out a connection with the study vaccine.

7.4.6 Suspected Unexpected Serious Adverse Reaction (SUSAR)

A SUSAR is a serious adverse reaction whose nature and severity exceed what is outlined in the Investigator’s Brochure, the approved product label, or summary of product characteristics for marketed drugs.

During vaccine clinical trials, the sponsor is required to promptly report any confirmed or suspected SUSARs related to the study vaccine to the research team, ethics committee, Center for Drug Evaluation (CDE), and national health authorities. Upon receiving a SUSAR report from the sponsor, the investigator should immediately submit it to the ethics committee. If the sponsor and investigator disagree on causality with the vaccine but either party considers a connection possible, the sponsor must still expedite the report.

For SUSARs that are life-threatening or fatal, the sponsor must report as soon as possible within seven days of first awareness, with follow-up information provided within the subsequent eight days (note: the day the sponsor first becomes aware is day

0). For SUSARs that are non-fatal and non-life-threatening, the report must be made as soon as possible within 15 days of awareness. The sponsor should also promptly report any other potential serious safety risks to the national drug evaluation authorities, providing medical and scientific assessments for each case. The sponsor must continue to follow up on SAEs, submitting additional information or updates to the previous report within 15 days of receiving new data.

In the event of a SUSAR, the sponsor, investigator, ethics committee, or regulatory authorities have the authority to propose pausing or terminating the study.

7.4.7 Adverse Events of Special Interest (AESIs)

AESIs, specifically suspected cases of herpes zoster, will be collected from the first dose through 30 days after completing the full immunization.

7.4.8 Disease-Related Events Not Meeting AE or SAE Criteria

1) Elective surgeries planned before subject enrollment are not reported as SAEs.
2) Pregnancy itself is not considered an AE.

7.4.9 Development Safety Update Report (DSUR)

The sponsor must submit the Development Safety Update Report (DSUR) to the National Medical Products Administration's Center for Drug Evaluation (CDE), all investigators and clinical study sites, and the ethics committee in accordance with regulatory and ethics committee requirements. The DSUR should include relevant information on SAEs occurring within six months after full immunization and an assessment of the study risks and benefits.

7.5 Pregnancy and Postpartum Information

7.5.1 Subjects Who Become Pregnant During the Study

Pregnancy is an exclusion criterion for each vaccine dose, and women of childbearing potential are required to use effective contraception for eight months following the first vaccine dose. However, accidental pregnancies may still occur during participation. Any pregnancy occurring from the time of vaccination through six months after full immunization must be reported, and the investigator is required to complete a "Pregnancy Event Report Form."

Researchers will closely follow pregnant subjects to obtain pregnancy outcome information (e.g., details of delivery and newborn status, or termination of pregnancy) and update the "Pregnancy Event Report Form."

Pregnancy itself is not considered an AE, but any complications during pregnancy are considered AEs and, in certain cases, may be classified as SAEs, such as spontaneous

abortion, stillbirth, or congenital anomalies in the infant. Elective abortions due to personal choice, where no fetal abnormalities are present, are not considered adverse events.

Handling Pregnancy Events During Vaccination:

If pregnancy occurs after the first dose but before the completion of the full vaccination course, the subject should not receive the second dose until the pregnancy ends (whether full-term, miscarriage, or abortion). The investigator will maintain regular contact with the subject for pregnancy assessments.

If pregnancy is discovered after the subject has completed the full vaccination course, the subject may complete study visits per the protocol and investigator discretion.

7.6 Immunogenicity Evaluation

Evaluable subjects (those meeting all inclusion criteria, adhering to protocol-defined procedures and intervals, and without any exclusion criteria) will have their immunogenicity assessed using data obtained from ELISA and flow cytometry. The immunogenicity metrics include seroconversion rate, anti-gE antibody GMC, geometric fold increase, and the proportion of VZV gE-specific CD4+/CD8+ T cells expressing at least two cytokines (TNF-α, IFN-γ, IL-2, CD40L), comparing post-vaccination results to baseline.

7.6.1 Biological Sample Collection

Blood sample collection: Section 3.1.1.1. Suspected herpes zoster case sample collection: Section 3.1.1.2.Urine sample collection: Section 3.1.1.3

7.6.2 Biological Sample Processing

Serum Antibody Testing Sample:

Collect 4.0-5.0 mL of venous blood. Separate the serum within 24 hours and divide it into three tubes: one for testing and two as backups. The test sample should contain at least 0.5 mL and be stored below -20°C. Serum samples must remain frozen during transport, with dry ice used if necessary.

Cellular Immunity Testing Sample:

1) Collect 15.0-20.0 mL of venous blood into an anticoagulant tube (EDTA.K2, sodium heparin, or lithium heparin), gently inverting to mix with the anticoagulant. Keep the tube upright at room temperature, labeled correctly.
2) To avoid hemolysis, control the flow to allow blood to flow gently along the tube wall.
3) Within 3 hours of collection, mix fresh anticoagulated blood with saline (1:1 ratio),

carefully layer over lymphocyte separation medium (double the volume of anticoagulated blood), and centrifuge at 400g for 20 minutes. Collect the second layer of cells. Follow the “Human Peripheral Blood Lymphocyte Separation Medium Manual” for detailed instructions.

4) Add 4 mL of cell cryopreservation solution to the separated lymphocytes, aliquot into four cryovials (minimum 0.8 mL per tube), gradually cool, and store in liquid nitrogen.
5) Transport frozen samples with liquid nitrogen.
6) Backup samples should be stored at a third-party laboratory designated by the sponsor.

Herpes Zoster Suspected Case Biological Sample:

Place the swab or crust sample into a tube with viral preservation solution, store at -70°C or lower within 4 hours, and transport on dry ice to the testing laboratory.

7.6.3 Biological Sample Numbering Rules

Each biological sample should correspond to the subject study ID. Specific numbering guidelines should follow the relevant SOP.

7.6.4 Biological sample testing

Immunogenicity Biological Samples

A third-party testing agency will be commissioned to use the ELISA method to detect the anti-gE antibody levels in all serum samples from subjects.

A third-party testing agency or XXX central laboratory will be commissioned to use flow cytometry to analyze gE-specific CD4+/CD8+ T cell activation cytokines and the proportion of cells active for at least two cytokines (TNF-α, IFN-γ, IL-2, CD40L). Detailed procedures for cellular immunity indicators can be found in the operation manual or SOP of the third-party testing agency or the XXX central laboratory.

8. Statistical Considerations

8.1 Statistical Hypotheses

This study hypothesizes that, 30 days after full immunization with the study vaccine, the proportion of gE-specific CD4+/CD8+ T cell activation with at least two cytokines (TNF-α, IFN-γ, IL-2, CD40L) is non-inferior to that of the active control vaccine. The statistical hypotheses are as follows:

H10: On day 30 post-full immunization, the difference in the proportion of specific CD4+ T cell activation with at least two cytokines (TNF-α, IFN-γ, IL-2, CD40L)

between the experimental vaccine group and the active control vaccine group is ≤ -10%.
H11: On day 30 post-full immunization, the difference in the proportion of specific CD4+ T cell activation with at least two cytokines (TNF-α, IFN-γ, IL-2, CD40L) between the experimental vaccine group and the active control vaccine group is > -10%.
H20: On day 30 post-full immunization, the difference in the proportion of specific CD8+ T cell activation with at least two cytokines (TNF-α, IFN-γ, IL-2, CD40L) between the experimental vaccine group and the active control vaccine group is ≤ -10%.
H21: On day 30 post-full immunization, the difference in the proportion of specific CD8+ T cell activation with at least two cytokines (TNF-α, IFN-γ, IL-2, CD40L) between the experimental vaccine group and the active control vaccine group is > -10%.

8.2 Sample Size Determination

Assuming that, 30 days after full immunization, the proportion of gE-specific CD4+ T cell activation with two or more cytokines (IFN-γ, TNF-α, IL-2, CD40L) is 85% in the experimental vaccine group and 75% in the active control group, and the proportion of gE-specific CD8+ T cell activation with two or more cytokines is 55% in the experimental vaccine group and 45% in the active control group, with an assumed dropout rate of 10%, a sample size of 150 subjects per group will yield 135 evaluable subjects per group. At a one-sided significance level of 0.025, this sample size provides approximately 90% power to test the above non-inferiority hypothesis.

8.3 Analysis Sets

Full Analysis Set (FAS): This includes all subjects in both the experimental vaccine group and the similar active control vaccine group who follow the intention-to-treat (ITT) principle. Subjects must have been randomized, completed vaccination, and have baseline and at least one post-immunization immunogenicity result. For subjects with vaccination number errors, immunogenicity evaluation will follow the original randomized group.
Per-Protocol Set (PPS): This set includes all subjects in the experimental and active control vaccine groups who meet inclusion/exclusion criteria, are randomized, complete vaccination, and have baseline and 30-day post-full immunization immunogenicity results. Subjects with major protocol deviations impacting immunogenicity evaluation will be excluded, though data prior to deviations may still be included in the analysis.
Safety Set (SS): This includes all subjects who received any dose of the vaccine.
Safety analyses for each dose will be based on the actual number of subjects who received each dose. Specifically:The first-dose safety set includes all randomized subjects who received the first dose of the study vaccine.The second-dose safety set includes all randomized subjects who completed the second dose of the study vaccine.

Safety analyses for each dose will follow the ASAT (All Subjects As Treated) principle, analyzing subjects based on the vaccine group they actually received.
The determination of the above analysis sets will occur in a blinded data review meeting before database lock, with input from the primary investigator, sponsor, statisticians, and data managers.

8.4 Analysis Methods

8.4.1 General Principles

Quantitative data will be statistically described using mean, median, standard deviation, maximum, and minimum values. Categorical or ordinal data will be presented with frequency and percentage. Antibody levels for immunogenicity indicators will be expressed using geometric mean, median, maximum, minimum values, and 95% confidence intervals. Antibody levels will undergo log transformation for statistical analysis.
All statistical analyses will be conducted using SAS software version 9.4 or higher.

8.4.2 Cellular Immunogenicity

For both the experimental vaccine group and the active control vaccine group, the proportion of gE-specific CD4+ T cell and CD8+ T cell activation with at least two cytokines (IFN-γ, TNF-α, IL-2, CD40L) at 30 days post-full immunization will be calculated along with their 95% confidence intervals (using the Clopper-Pearson method). The difference in proportions between the experimental vaccine group and the active control group and its 95% confidence interval (using the Miettinen and Nurminen method) will also be calculated. If the lower limit of the 95% confidence interval for the difference is greater than -10%, non-inferiority is established. Cellular immunogenicity analysis will primarily be based on the per-protocol set.

8.4.3 Humoral Immunogenicity

Thirty days after full immunization, the anti-gE antibody concentration will undergo a logarithmic transformation, and an analysis of covariance model will be used to analyze the data. In this model, the logarithmic transformation of the antibody concentration at 30 days post-full immunization will be the dependent variable, the logarithmic transformation of the pre-immunization antibody concentration will be the covariate, and the group will be the fixed effect. Based on this model, the least squares mean of the log-transformed geometric mean concentration (GMC) of anti-gE antibodies at 30 days post-full immunization for each group and the between-group difference (experimental vaccine group - active control vaccine group) will be calculated. After

back-transformation, the GMC of anti-gE antibodies at 30 days post-full immunization for each group and their 95% confidence intervals, as well as the between-group ratio (experimental vaccine group / active control vaccine group) and its 95% confidence interval, will be computed.

The seroconversion rate of anti-gE antibodies at 30 days post-full immunization (defined as at least a fourfold increase in antibody levels post-immunization) will be calculated for both the experimental vaccine group and the active control vaccine group. The 95% confidence interval for each group rate will be calculated using the Clopper-Pearson method.

The geometric mean fold rise in anti-gE antibodies from pre-immunization (Day 0) to 30 days post-full immunization will be calculated for each group, along with its 95% confidence interval.

Reverse cumulative distribution plots of anti-gE antibody titers will be created separately for the experimental vaccine group and the active control vaccine group at pre-immunization and at 30 days post-full immunization.

Humoral immunogenicity analysis will primarily be based on the per-protocol set.

8.4.4 Safety

This study will primarily focus on the statistical analysis of treatment-emergent adverse events (TEAEs) occurring after vaccination. Adverse events occurring before vaccination will be listed separately. Unless otherwise specified, the term "adverse events" in the following sections refers to TEAEs.

Solicited adverse events occurring 0-7 days post-vaccination, as well as solicited adverse events related to vaccination, will be categorized and analyzed by group, type of reaction (systemic and local), and the severity of each solicited adverse event as listed in Section 3.1.3.2. For these events, the number of adverse events, the number of subjects experiencing them, and the incidence rate will be calculated.

The following adverse events will be categorized and analyzed by group, System Organ Class (SOC), and Preferred Term (PT), calculating the number of events, the number of affected subjects, and the incidence rate:

- Unsolicited adverse events occurring 0-30 days after each dose;
- Adverse events occurring within 30 minutes after each dose;
- All adverse events occurring 0-30 days after each dose;
- Adverse events of special interest occurring from the first dose to 30 days after full immunization;
- Serious adverse events occurring from the first dose to 6 months after full immunization.

8.5 Protocol Deviations

Protocol Deviations refer to any modifications or actions that do not follow the study

protocol design or procedures without prior approval from the ethics committee. Actions that do not impact subject rights, safety, benefits, or the integrity, accuracy, and reliability of trial data, nor affect safety or primary endpoint evaluations, are considered protocol deviations. Actions that impact subject rights, safety, benefits, or the integrity, accuracy, and reliability of trial data, as well as those affecting safety or primary endpoint evaluations, are considered serious protocol deviations (protocol violations).

Examples of protocol deviations (including but not limited to):

1) Subjects did not sign the updated version of the informed consent form in a timely manner.
2) Storage and management of the study vaccine did not follow protocol requirements, or vaccine use was not confirmed as suitable before administration.
3) Diary cards and contact cards were not collected or distributed as required.
4) Other deviations.

Examples of protocol violations (including but not limited to):

1) Subjects entered the study without informed consent.
2) Subjects did not meet any inclusion criteria but were enrolled in the study.
3) Subjects met any exclusion criteria but were enrolled in the study.
4) Subjects did not receive the study vaccine according to the assigned vaccine allocation.
5) Vaccine dose, frequency, or administration route did not comply with protocol requirements.
6) Subjects used prohibited medications or those restricted by the protocol.
7) Subjects met withdrawal criteria but were not withdrawn from the study.
8) Other violations.

For protocol deviations/violations occurring during the study, site investigators must report the facts, process, cause, and impact of the event to the study responsible organization. The principal investigator or lead collaborative investigator should provide guidance on handling the event. Serious protocol deviations/violations must be reported to the ethics committee.

Researchers should provide targeted training for personnel involved in protocol violations to prevent recurrence and document the training process.

9. General Considerations: Regulatory, Ethical, and Trial Oversight

9.1 Regulatory and Ethical Considerations

This study will be conducted in accordance with the protocol and the following guidelines:

1) Consensus ethical principles derived from international guidelines, including the Declaration of Helsinki and the International Ethical Guidelines by the Council for

International Organizations of Medical Sciences (CIOMS).

2) Good Clinical Practice (GCP) guidelines.
3) Guiding Principles for Quality Management of Vaccine Clinical Trials (Provisional).

9.1.1 Investigator Responsibilities

The responsible organization for the vaccine clinical trial should establish a comprehensive organizational and quality management system, including mechanisms and measures to prevent and manage emergencies that may arise during the study. It must have a team of SAE (Serious Adverse Event) emergency response experts and the technical capability to handle serious adverse events, as well as complete vaccine transportation, storage, and cold chain equipment.

The study site for the vaccine clinical trial must be approved for vaccination by health authorities and have a stable and enough clinical trial personnel. It must be equipped with standard operating procedures (SOPs) related to vaccine clinical trials, conduct training with records of attendance, and establish an SAE medical emergency pathway in collaboration with local medical institutions. Based on the vaccination and visit flow of the vaccine clinical trial, the site should include designated areas for reception, informed consent, consultation and screening, biological sample collection, vaccination, emergency treatment, medical observation, vaccine storage, record-keeping, sample processing and preservation, screening laboratory, and temporary medical waste storage. An emergency pathway should be established, and the study site should have an ambulance, related rescue personnel, and emergency supplies available.

All personnel roles and responsibilities must be confirmed by the principal investigator or lead collaborator to ensure that all researchers participating in this project are qualified, trained, and authorized, with clear tasks and mastery of relevant SOPs. The responsible organization and study site personnel should undergo training in GCP and vaccine clinical trial techniques, with training records maintained. Auxiliary personnel should also have records of participation in relevant training.

9.1.2 Sponsor Responsibilities

The sponsor holds ultimate responsibility for the quality of the study. The sponsor should establish a comprehensive quality management system for vaccine clinical trials, develop corresponding standard operating procedures (SOPs), and organize audits of the study. Systematic checks of trial-related activities and documentation—including study sites, laboratories, and Contract Research Organizations (CROs)—should be conducted to evaluate whether the study is being carried out according to the protocol, SOPs, and regulatory requirements, and whether study data is recorded promptly, truthfully, accurately, and completely. Audits should be conducted by personnel not directly involved in the study.

The study site should cooperate with study audits, maintain relevant records, and

address issues identified during audits by creating improvement plans and implementing appropriate management measures to enhance study quality.

9.2 Ethics Committee

The ethics committee is responsible for reviewing the scientific and ethical soundness of the vaccine clinical trial to ensure the dignity, safety, and rights of subjects, promoting the scientific and healthy development of vaccine trials, and fostering public trust and support.

The ethics committee of the research institution may review the study protocol, the informed consent form, recruitment materials, and other written materials provided to subjects. Revisions to the protocol require consultation with the sponsor, while suggested changes to the informed consent form that do not contradict the protocol and align with local conditions may be adopted based on the ethics committee recommendations.

9.2.1 Follow-Up Review

The ethics committee should conduct follow-up reviews for all approved clinical trials until the study is completed.

9.2.2 Amendment Review

Any modifications to the study protocol during the study must be submitted to the ethics committee for review and approval or filing before implementation. The ethics committee should request the sponsor and/or investigator to submit relevant information for the amendment review, including (but not limited to):

1) Details and reasons for the modifications;
2) Impact of the modification on anticipated risks and benefits;
3) Impact of the modification on the rights and safety of subjects.

The ethics committee will primarily assess the risks and benefits of the modified protocol and issue a review opinion. If protocol modifications are necessary to prevent immediate harm to subjects, the investigator may implement changes prior to ethics committee approval but must promptly provide a written report to the committee afterward.

9.2.3 Annual/Periodic Follow-Up Review

During the initial review, the ethics committee should determine the frequency of annual or periodic follow-up reviews based on the level of study risk, with reviews occurring at least once a year. The ethics committee should require the investigator to submit timely reports, with information for annual/periodic follow-up reviews including (but not limited to):

1) Progress of the study;
2) Number of subjects enrolled, completed, and withdrawn;
3) Confirmation that serious adverse events are reported promptly and handled appropriately;
4) Any events or new information that could affect the study risk-benefit profile.

After reviewing the study progress, the ethics committee will reassess the risks and benefits of the study.

9.2.4 Serious Adverse Event Review

The ethics committee must review serious adverse events reported by the sponsor and/or investigator, including the severity and scope of the events, their impact on the study risk-benefit profile, and the medical protection measures in place for subjects.

9.2.5 Non-Compliance/Protocol Violation Review

For instances of non-compliance or protocol violations occurring during the study, the ethics committee should require the sponsor and/or investigator to provide an explanation of the cause, impact, and corrective measures for the incident. The committee will review whether the event affects subject safety and rights or the study risk-benefit balance.

9.2.6 Premature Termination Review

If the sponsor and/or investigator decides to terminate the study prematurely, they must submit this decision for ethics committee review. The ethics committee should require the sponsor and/or investigator to report the reasons for the early termination and the follow-up measures for subjects, ensuring that subject safety and rights are adequately protected.

9.2.7 Study Completion Review

The ethics committee should require the sponsor and/or investigator to report on the completion status of the study and review the measures taken to protect the safety and rights of subjects.

9.3 Informed Consent Process

The informed consent process must be completed before any study procedures begin. Prior to enrollment, the researcher should provide the subject with relevant information about the study. Both the subject and the researcher will sign the informed consent form, which will be prepared in duplicate: one copy for the subject to keep and one to be retained at the study site.

9.4 Early Closure of Research Center or Study Termination

If the study is terminated early, the sponsor will promptly notify the investigators, the Institutional Review Board (IRB), and regulatory authorities of the reasons for early termination in accordance with applicable registration regulations.

Regardless of the reason for early termination, the investigator must promptly inform the subjects and ensure appropriate follow-up for them.

After study termination, the study may only continue once issues related to safety, protocol compliance, and data quality are resolved, and requirements from the sponsor, ethics committee, and/or national regulatory authorities are met.

For specific trial termination criteria, refer to Section 6.6.

10. General Considerations: Risk Management and Quality Assurance

10.1 Data Quality Assurance

10.1.1 Investigator

The responsible organization for the vaccine clinical trial should establish a comprehensive organizational and quality management system, including mechanisms and measures to prevent and handle emergencies that may arise during the study. It must also have complete equipment for the transport and cold chain storage of vaccines.

The study site must be approved by the relevant health authorities for vaccination and have a stable, adequate number of study personnel. It should be equipped with standard operating procedures (SOPs) related to vaccine clinical trials, maintain training records, and collaborate with local medical institutions to establish an emergency medical treatment pathway for vaccine clinical trial serious adverse events (SAEs). In line with the vaccine study vaccination and visit procedures, the site should include dedicated areas for reception, informed consent, consultation and screening, biological sample collection, vaccination, emergency treatment, medical observation, vaccine storage, record-keeping, sample processing and preservation, and temporary storage of medical waste. An emergency pathway should be in place, and the site should have an ambulance, trained rescue personnel, and emergency supplies.

All roles and responsibilities of the research staff must be confirmed by the principal investigator or lead collaborator, ensuring that all individuals participating in this project are qualified, trained, and authorized, with clear task assignments and knowledge of relevant SOPs. Personnel at the responsible organization and study site must undergo training in Good Clinical Practice (GCP) and vaccine clinical trial techniques, with training records maintained. Auxiliary personnel must also have records of relevant training.

10.1.2 Sponsor

The sponsor holds ultimate responsibility for the quality of the study. The sponsor should establish a comprehensive quality management system for vaccine clinical trials, develop corresponding standard operating procedures (SOPs), and organize audits of the study. Systematic checks of trial-related activities and documentation—including study sites, laboratories, and Contract Research Organizations (CROs)—should be conducted to evaluate whether the study is being conducted according to the protocol, SOPs, and regulatory requirements, and whether study data is recorded promptly, truthfully, accurately, and completely. Audits should be conducted by personnel who are not directly involved in the study.

10.1.3 Clinical Research Associate (CRA)

The sponsor should assign an adequate number of Clinical Research Associates (CRAs) to monitor the study throughout its duration, as specified in the Guiding Principles for Quality Management of Vaccine Clinical Trials (Provisional). CRAs should have relevant education and work experience in fields such as medicine or pharmacy. The sponsor should determine the number of CRAs based on factors like the required monitoring frequency and the complexity of the trial protocol. CRAs are to perform monitoring activities per the monitoring plan and submit monitoring reports.

CRAs are responsible for overseeing the entire study process to ensure compliance with the protocol, SOPs, Good Clinical Practice (GCP), and relevant regulations, ensuring the study is completed within the planned timeframe.

10.1.4 Protocol Amendments

Any modifications to this study protocol must be discussed and approved by the sponsor. If there is consensus on the necessity of the modification, the sponsor will document it in writing, and the revised protocol will replace the previous version. All protocol amendments must be submitted to the Institutional Review Board (IRB); significant amendments (e.g., those impacting trial conduct or subject safety) require IRB approval. Administrative changes that do not affect the trial design, objectives, or subject safety may be submitted for IRB filing or expedited review, as per IRB requirements.

Investigators are responsible for ensuring that any protocol modifications needed during the study are not implemented before IRB review and approval, except when changes are necessary to eliminate immediate risks to the subjects.

10.1.5 Biological Sample Management

Subject blood samples used for immunogenicity testing are to be managed by designated personnel, with established protocols for sample storage and temperature monitoring. On weekdays, storage temperatures must be monitored and recorded daily. On holidays, monitoring and recording may be arranged according to site-specific conditions, provided automatic temperature monitoring and alarm systems are in place.

Whole blood samples should be transported to the testing laboratory or sample processing laboratory at 2-8°C. Processed cellular samples must be stored and transported under low-temperature conditions (liquid nitrogen) to the testing laboratory. Serum samples should be transported to the testing laboratory under frozen conditions (using dry ice, low-temperature ice packs, or other freezing methods). Backup samples should not be transported simultaneously with test samples and must be stored by the investigator at ≤ -20°C until the clinical report is completed and processed as confirmed by the sponsor.

Blood samples should be transported to the testing center in batches according to the relevant SOPs.

Proper handover management must be maintained throughout the process, with the investigator, sample transporter, and laboratory personnel keeping accurate records.

10.1.6 Vaccine Management

The organization responsible for the vaccine clinical trial should guide the study site in establishing a management system for study vaccines. The processes of receiving, storing, preparing, retrieving, returning, or destroying study vaccines must comply with relevant laws and regulations. Both the responsible organization and the study site should designate personnel trained in Good Clinical Practice (GCP) and related protocols to manage study vaccines.

Vaccine Transportation: Throughout the management process, cold chain requirements must be met, with proper transport and storage conditions as outlined in the protocol. The transportation process should include a shipment list and temperature monitoring. Upon arrival, packaging conditions and opening temperature should be recorded. The recipient must sign the shipment list upon verification and send a faxed or copied record to the sender, with both parties retaining the document.

Vaccine Handover Record: The sponsor will provide the experimental and control vaccines. The investigator, upon receipt, must verify the vaccine name, quantity, and packaging integrity and complete a handover record.

Vaccine Storage and Distribution: Study vaccines must be managed in separate, locked storage units with designated personnel. For blinded studies, measures must be in place to maintain blinding. Upon receiving vaccines, the recipient must verify transport conditions, establish tracking, registration, usage, and retrieval logs, and store them in work records as required.

Vaccine Usage Record: The investigator should maintain a record of vaccine registration and usage. Each dose dispensed to a subject should be documented, including study ID, subject’s initials, and the administering person’s signature. Study vaccines must not be used for non-trial individuals.

Vaccine Retrieval Record: The vaccine manager should retrieve unused vaccines promptly, conduct regular inventory counts, and record the results. If usage and

remaining quantities do not match the total amount, an explanation is required. All discarded, expired, or leftover vaccines should be returned to the sponsor. The sponsor, upon receipt, must verify the quantity and complete related records, with signatures from the vaccine manager and sponsor representative.

Cold Chain Disruption: A cold chain disruption is defined as a temperature excursion in the vaccine storage refrigerator exceeding 8°C but remaining below 37°C for more than 24 hours, or when the refrigerator temperature drops below 2°C, resulting in vaccine freezing. The sponsor should specify relevant information on vaccine stability in the Investigator's Brochure. In the event of a cold chain disruption, the investigator should promptly transfer the vaccine to a 2-8°C, light-protected environment, discontinue the use of the affected vaccine, and report to the sponsor as soon as possible. Further use of the vaccine should be determined based on the sponsor's written guidance.

10.1.7 Instrument and Equipment Calibration

Disposable sterile syringes are used for vaccine injection and blood collection, manufactured by licensed companies, with lot numbers and expiration dates recorded.

Measuring instruments such as height gauges, scales, and blood pressure monitors are qualified and in active use.

Thermometers used for monitoring refrigerator temperatures have been calibrated and are within the validity period; continuous temperature monitoring records for three consecutive days are required before the refrigerator is put into use.

Cold storage equipment used on-site for storing vaccines and samples undergoes annual inspection and is in active use.

10.1.8 Original Data Management

Documents such as the informed consent form, vaccination and follow-up records, diary cards, contact cards, and SAE report forms serve as essential sources for clinical trial traceability and must be recorded promptly, accurately, completely, standardized, and truthfully. These records should be securely maintained at the study site.

Authorized and specially trained researchers will enter study data from original records into the electronic Case Report Form (eCRF). Data should not be altered arbitrarily; any necessary corrections due to entry errors must be made according to the completion guidelines. To ensure the accuracy and reliability of study data, the eCRF will be jointly reviewed by monitors and researchers. After the researcher signs off on the data, all materials will be submitted to the clinical trial responsible unit or sponsor, who will assign statistical personnel for data processing.

10.1.9 Study Documentation

The sponsor and research team must manage study documentation in accordance with the Drug Registration Management Measures and Good Clinical Practice (GCP)

guidelines.

The investigator’s file should be organized per GCP requirements and stored at the study site. Records containing actual subject information, such as screening logs, informed consent forms, vaccination and follow-up logs, diary cards, contact cards, and subject medical records, should be securely stored on-site. The research institution coordinator and on-site archive manager should complete an inventory handover, with both parties signing a storage agreement or memorandum.

Archive management should follow standard operating procedures (SOPs), with labels that include the project name, completion date, sponsor, and retention period. Safety measures should be in place to prevent pests, moisture, fire, and theft. Access to and use of these records are restricted to the project researchers. However, in compliance with confidentiality principles and relevant regulations, monitors, auditors, ethics review committees, and regulatory authority inspectors may review subjects' original medical records to verify the trial process and data.

The investigator and sponsor should retain study documentation for at least five years after the study vaccine is approved for marketing. Upon reaching the retention period, the investigator should notify the sponsor, and no one may dispose of the records without the sponsor’s written approval.

10.2 Source Data

10.2.1 eCRF Design

The electronic Case Report Form (eCRF) is designed based on the study procedures and flowchart specified in the protocol. After the initial draft is created, it requires a joint review by project team members, including the project manager, data and statistical personnel, and protocol authors. The eCRF must align with the protocol, comply with relevant laws and regulations, and have a documented version control process to ensure traceability and accuracy.

10.2.2 eCRF Completion Guidelines

The eCRF Completion Guidelines provide specific instructions for filling out each form and data point on the Case Report Form, based on the study protocol. The study site must receive the eCRF and its completion guidelines before enrolling subjects. Staff at the study site should be trained on the protocol, eCRF completion, and data submission procedures, with this training process documented and archived.

10.2.3 eCRF Annotation

The annotated eCRF is a labeled version of the blank eCRF, indicating the location of each data item and its variable name and coding in the database. All data items within the eCRF must be annotated. This annotated version requires review by Data

Management (DM).

10.2.4 Database Design

The database should be established according to the dataset names, variable names, variable types, and variable lengths as specified in the annotated eCRF, adhering as closely as possible to standard database structures and settings. Upon completion, the database should undergo testing, with a database testing report prepared and signed off by the data management lead for confirmation.

10.2.5 Permission Allocation

The system administrator will create accounts for each role and assign specific permissions according to the role requirements.

10.2.6 eCRF Completion

Researchers must collect subject data according to the study protocol and reference the completion guidelines to accurately, promptly, completely, and consistently enter information into the eCRF based on original source data. Any modifications to eCRF data must follow standard operating procedures, with audit trails maintained for all changes.

10.2.7 Query Issuance and Resolution

The Data Management (DM) team will create a detailed Data Review Plan, which must be reviewed and signed off by programmers and the DM project manager (PM) to confirm agreement. After data is entered into the Electronic Data Capture (EDC) system, the system will perform data checks according to the Data Review Plan, using pre-established edit checks to identify data queries automatically. For data that cannot be flagged by automated checks, manual queries will be issued through the EDC. Data entry personnel or researchers must review and respond to both system and manual queries, correcting data if necessary until the query is resolved. If the response does not resolve the query, the data manager and clinical research associate may re-query the data point, with all actions and audit trails recorded in the EDC database.

10.2.8 Data Modification and Review

Data entry personnel or researchers may modify data after verification. For any data modifications, a reason must be provided in the system as prompted. The researcher has the authority to review and approve all final data.

10.2.9 Medical Coding

Medical history and adverse events collected in the study should be coded using standard dictionaries, typically MedDRA and WHODrug Dictionary (WHODD). The

dataset with coded data must clearly document the dictionary and version used for coding.

10.2.10 SAE Consistency Comparison

All SAE-related data points in the eCRF should be programmatically compared with corresponding data points in the Pharmacovigilance (PV) database. Any discrepancies must be discussed with PV personnel and resolved until there are no differences in the data.

10.2.11 Data Review Meeting

Before database lock, the data manager will draft an initial data review report and compile all data listings. The sponsor, researchers, data managers, and statistical analysts will jointly conduct a final review of the database. During this meeting, they will define statistical analysis populations according to the study protocol, verify serious adverse event reports, and review recorded handling information. Following the data review meeting, the finalized data review report and population classification plan resolutions will be documented.

10.2.12 Database Lock and Unlock

Database lock is a key milestone in the study process. The locking process and timing should be clearly documented. Locking restricts edit permissions on the database, preventing any unauthorized accounts from making changes.

If modifications are required after the database is locked, an application must be submitted. The sponsor, researcher, data entry personnel, clinical research associate, and data manager must discuss, approve, and sign off on the request. The reason for unlocking the database must be thoroughly documented.

10.2.13 Data Backup and Recovery

Throughout the data management process in the study, the database should be regularly backed up. In the event of irreparable database damage, the most recent backup should be used for recovery, with any additional data re-entered as needed. The final dataset will be backed up on a read-only CD. All relevant computers must have up-to-date and effective antivirus protection.

10.2.14 Data Storage

During the study, all collected original data (such as eCRF and electronic data) should be securely stored. These original documents are part of the audit trail that traces back to the source data and should be protected with the same rigor as the electronic audit trail, which records any modifications or backups of the database.

10.2.15 Data Confidentiality

Data confidentiality is a fundamental principle in clinical research and development. Organizations involved in drug development should establish appropriate procedures to ensure database confidentiality. This includes creating and signing confidentiality agreements to regulate personnel conduct and implementing security systems to prevent data breaches.

11. Appendix: Adverse Events (AEs) and Serious Adverse Events (SAEs) – Definitions, Severity, and Causality

11.1 Further Details and Explanations on the Definition of AE

Adverse Event (AE): Any unfavorable medical occurrence in a subject after receiving the study product, which may present as symptoms, signs, diseases, or laboratory abnormalities. These events do not necessarily have a causal relationship with the study product.
Pregnancy-related events are not reported as AEs.

11.2 Further Details and Explanations on the Definition of SAE

- The occurrence of herpes zoster (HZ) or postherpetic neuralgia (PHN) does not constitute an SAE.
- Elective surgeries planned prior to subject enrollment are not reported as SAEs.

11.3 Severity

The severity of adverse events following vaccination is assessed according to the Guidelines for Grading Adverse Events in Preventive Vaccine Clinical Trials, issued by the NMPA on December 31, 2019. Adjustments have been made to the grading criteria based on the characteristics of subjects in this study. The grading standards for adverse event severity are as follows:

Tanble 4 Grading table of adverse events at inoculation site (local)

Symptom/Sign	Grade 1	Grade2	Grade3	Grade4
Pain				
Pain	Minimal or mild discomfort, no impact on limb activity	Affects limb activity	Affects daily life	Loss of basic self-care or hospitalization
Induration*, Swelling** (Optional)#				

>14 years	Diameter 2.5–<5 cm or area 6.25–<25 cm², no impact on daily life	Diameter 5–<10 cm or area 25–<100cm², no impact on daily life	Diameter ≥10 cm or area ≥100 cm² or causes symptoms such as restricted limb movement, lymphadenopathy, or drainage affecting daily life	Blistering, ulcerative dermatitis, necrosis, or deep tissue necrosis
Erythema*, Redness** (Optional)#				
>14 years	Diameter 2.5–<5 cm or area 6.25–<25 cm², no impact on daily life	Diameter 5–<10 cm or area 25–<100 cm², affects daily life	Diameter ≥10 cm or area ≥100 cm² or associated symptoms affecting daily life	Blistering, ulcerative dermatitis, necrosis, or deep tissue necrosis
**Other**				
Itching	Itching at the injection site that relieves within 48 hours after self-care or treatment	Itching at the injection site that does not resolve within 48 hours after treatment	Affects daily life	NA
Cellulitis	NA	Need for non-injectable treatment (e.g. oral antibacterial, antifungal, antiviral)	Intravenous therapy is required (e.g. intravenous antibacterial, antifungal, antiviral)	Sepsis, or Tissue Necrosis, etc.

Notes:

* In addition to grading based on direct diameter measurements, the progression of measurement results should also be recorded.

** The maximum measured diameter or area should be used.

The grading of induration and swelling, as well as rash and erythema, should be based on functional impact and actual measurements, with the higher grading criterion being selected.

Table 5 Grading of Vital Signs

Vital Sign	Grade 1	Grade2	Grade3	Grade4
Fever (Axillary Temperature, °C)				
>14 years	37.3～<38.0	38.0～<38.5	38.5～<39.5	≥39.5, lasting more than 3 days

Table 6 Systemic (Non-Local) Adverse Event Grading

Organ System / Symptom	Grade 1	Grade2	Grade3	Grade4
Gastrointestinal System				
Diarrhea	Mild or transient, 3–4 times/day, loose stools, or	Moderate or persistent, 5–7 times/day, loose stools, or	≥7 times/day, loose stools, or resulting in severe symptoms such as hypotension,	Life-threatening, requiring hospitalization

	mild cramping lasting ≤1 week	cramping lasting >1 week	electrolyte imbalance, dehydration, or needing IV fluids >2L	
Constipation*	Requires stool softeners or dietary adjustment	Requires laxatives	Needs manual disimpaction or enemas	Severe toxicity causing intestinal obstruction
Dysphagia	Discomfort with swallowing solids	Limited to soft diet	Limited to liquid diet, cannot tolerate solid foods	Unable to tolerate any oral intake, requires tube feeding
Appetite Loss	Appetite decreases but does not reduce food intake significantly	Decreased intake, significant reduction	Appetite decrease with body weight loss or nutritional decline	Requires nutritional support (e.g., tube feeding or intravenous nutrition)
Vomiting	1–2 times/24 hours, minimal impact on activity	3–5 times/24 hours, activity affected	≥6 times/24 hours or requiring IV fluids	Life-threatening, requires hospitalization or urgent care
Nausea	Mild (<24 hours) with normal food intake	Persistent, affects intake to some extent (24–48 hours)	Persistent nausea limiting food intake ≥48 hours or requiring IV fluids	Threatens life (e.g., risk of aspiration)
Musculoskeletal and Connective Tissue Disorders				
Myalgia (Non-Injection Site)	Does not affect daily activities	Affects daily activities	Severe muscle pain, significantly impacts daily activities	Emergency visit or hospitalization
Arthritis	Mild pain, with swelling, erythema, or joint stiffness, but does not limit function	Moderate pain, with swelling, erythema, or joint stiffness, but does not affect daily activities	Severe pain, requiring analgesics and/or mobility aids, impacting daily activities	Permanent disability or irreversible joint injury
Arthralgia	Mild pain, does not limit function	Moderate pain, may affect function	Severe pain requiring analgesics and/or mobility aids, impacting daily activitie	Persistent pain with functional loss
Nervous System				
Headache	Does not affect daily activities, no treatment needed	It is transient and mildly affects daily activities and may require treatment or intervention	Severe, impacts daily activities, requires intensive treatment	Life-threatening, requires emergency care or hospitalization
Syncope	Near syncope, no loss of consciousness (e.g., pre-syncope)	Loss of consciousness, but no intervention required	Loss of consciousness, requires intervention or hospitalization	NA
New-Onset Seizures				

≥18 岁	NA	NA	1–3 seizures	Prolonged or multiple seizures (e.g., status epilepticus) or difficult to control (e.g., life-threatening epilepsy)
Respiratory System				
Cough	Transient, no treatment needed	Persistent cough, requires treatment, ineffective	paroxysmal cough, uncontrolled by treatment	Emergency or hospitalization
Acute bronchospasm	Transient, no treatment needed; FEV1% 70–80%	Need medical treatment; Bronchodilator treatment returned to normal; FEV1% is 50% ~ 70%	Bronchodilator therapy does not return to normal; FEV1% is 25% ~ 50% or intercostal depression	Severe, FEV1% <25% or requires mechanical ventilation
Dyspnea (Breathing Difficulty)	Mild, occurs with exertion only	Moderate, occurs with normal activity	Severe, occurs with minimal activity	Life-threatening, occurs at rest
Skin and Subcutaneous Tissue Disorders				
Non-injection Site Rash (No Skin Damage)	Mild itching, does not affect daily life	Itching affects daily life	Severe itching prevents daily activities	NA
Skin/Mucosal Abnormalities	Erythema/itching/color change	Diffuse rash/maculopapular rash/dryness/desquamation	Vesicular/exudation/desquamation/ulceration	Exfoliative dermatitis involving mucous membranes, or erythema multiforme, or suspected Stevens-Johnson syndrome
Nervous System				
Insomnia*	Mild sleep difficulty, no or minimal impact on daily life	Moderate sleep difficulty, affects daily life	Severe sleep disturbance, significant impact on daily life, requires treatmentor hospitalization	NA
Irritability/Inhibition	Mild irritability or inhibition	Easily irritable or has difficulty sleeping	Lack of response to external stimuli or low awareness	NA
Psychiatric disorders (including anxiety, depression, mania, and confusion) should report detailed symptoms	Mild symptoms, does not require intervention or minimally affects daily life	With clinical symptoms requiring medical consultation or behavior affecting daily life	Requires hospitalization or inability to perform daily activities independently	Tendency to harm self or others, acute mental confusion, or loss of basic self-care ability
Immune System				

Acute Allergic Reaction**	Localized urticaria (water blisters), no systemic symptoms	Localized urticaria requiring treatment or mild angioedema not requiring treatment	Generalized urticaria or angioedema requiring treatment, or mild bronchospasm	Anaphylactic shock or life-threatening bronchospasm or laryngeal edema
Other				
Fatigue	No impact on daily activities	Affects normal daily activities	Severely affects daily activities, unable to work	Emergency visit or hospitalization
Pain at Non-Injection Sites# (Specify location at reporting)	Mild pain, no or minimal impact on daily life	Pain affects daily activities	Pain preventing daily activities	Disabling pain with loss of basic self-care ability

Notes:

FEV1% refers to the ratio of Forced Expiratory Volume in 1 second (FEV1) to Forced Vital Capacity (FVC).

* Changes in constipation and insomnia before and after vaccination should be monitored.

** Refers to Type I hypersensitivity reactions.

# Refers to non-injection site pain other than myalgia, arthralgia, or headache.

For adverse events not covered in the grading tables above, refer first to the Guidelines for Grading Adverse Events in Preventive Vaccine Clinical Trials. If the guidelines do not provide criteria for evaluation, assess the severity according to the following standards.

Table 7 General Grading Criteria for Other Adverse Events

Grade 1	Grade2	Grade3	Grade4	Grade5
Mild: Short duration (<48h) or slight discomfort, no impact on activities, no treatment required.	Moderate: Moderate or mild impact on activities, may require minimal or no treatment.	Severe: Significant impact on activities, requires intervention, may require hospitalization.	Life-threatening: Potentially life-threatening, severe impact on activities, requires intensive treatment.	Death.

11.4 Causality

The investigator should have measures in place to promptly assess the relationship between adverse events and vaccination, in order to detect SAEs and group-related or trend-related adverse events associated with vaccination during the study. This allows for timely suspension or termination of the study to minimize harm to subjects.

General principles of relevance judgment:

Definitely Related: Evidence of vaccination with the study vaccine exists; the timing of the adverse event is reasonable relative to vaccination; the adverse event is more reasonably explained by the study vaccine than by other causes; active response upon re-vaccination; the adverse event is consistent with prior knowledge of this vaccine or similar vaccines.

Probably Related: Evidence of vaccination with the study vaccine exists; the timing

of the adverse event is reasonable relative to vaccination; the adverse event is more reasonably explained by the study vaccine than by other causes.

Possibly Related: Evidence of vaccination with the study vaccine exists; the timing of the adverse event is reasonable relative to vaccination; the adverse event cannot be ruled out as being caused by the study vaccine, although other causes are also possible.

Unlikely Related: Evidence of vaccination with the study vaccine exists; the adverse event is more likely to be caused by other factors; negative or uncertain response upon re-vaccination.

Definitely Unrelated: The subject did not receive the study vaccine; or the timing of the adverse event is inconsistent with vaccination; or there is another significant cause that can explain the adverse event.

For statistical analysis, “Definitely Related,” “Probably Related,” and “Possibly Related” are considered adverse events "related" to the study vaccine, while “Unlikely Related” and “Definitely Unrelated” are considered adverse events "unrelated" to the study vaccine.

As the investigator’s understanding of AE/SAE is a progressive process, the initial report may contain uncertain causality information. As information accumulates or updates in subsequent visits, the investigator may revise the initial causality assessment. In such cases, AE/SAE follow-up is required, especially for SAEs, to ensure that relevant information is updated.

12. Appendix: Definitions and Supportive Operational Details

12.1 Contraception and Pregnancy Testing

12.1.1 Definitions Related to Fertility

Women of Childbearing Potential: Women from menarche until menopause are considered capable of childbearing unless it has been confirmed that they have undergone a hysterectomy, bilateral salpingectomy, and bilateral oophorectomy.

Postmenopausal Women: Women who have ceased normal menstruation for 12 consecutive months with no other medical cause.

12.1.2 Contraception

Female subjects must not be pregnant or breastfeeding. Women of childbearing potential (WOCBP) must use reliable contraceptive methods and should not have plans for pregnancy or childbirth within 8 months. Pregnancy is an exclusion criterion for each vaccine dose, and WOCBP are required to use effective contraception for 3 months following the first vaccine dose.

12.1.3 Pregnancy Testing

WOCBP (all women from menarche until menopause) must undergo a urine pregnancy test prior to each vaccination. The urine pregnancy test must be performed on the day of vaccination and before vaccination takes place. No sample storage is required after testing.

Pregnancy is an exclusion criterion for each vaccine dose, and WOCBP are required to use effective contraception for 8 months after the first vaccine dose. However, it is still possible for participants to become unintentionally pregnant during the study. Any pregnancy occurring after vaccination and throughout the study period should be reported, and the investigator must complete a "Pregnancy Event Report Form."

Researchers must closely follow up with pregnant subjects to obtain information on pregnancy outcomes (e.g., details of childbirth and neonatal status or pregnancy termination) and update the "Pregnancy Event Report Form."

Pregnancy itself is not considered a serious adverse event; however, any complications during pregnancy will be regarded as adverse events and may be considered serious adverse events in certain cases, such as spontaneous abortion, stillbirth, fetal death, and congenital abnormalities in the infant. Elective termination of pregnancy due to maternal choice, in the absence of fetal abnormalities, is not considered an adverse event.

Management of Pregnancy Events During Vaccination:

- If pregnancy occurs after the first dose but before the vaccination schedule is complete, the subject must not receive the second dose until the pregnancy has concluded (e.g., full-term birth, miscarriage, or abortion). The investigator will maintain regular contact with the subject to conduct pregnancy assessments.
- If pregnancy is discovered after the subject has completed the full vaccination schedule, the subject may complete study visits according to the trial protocol and at the discretion of the investigator.

13. Disclosure and Publication of Data

Upon completion of this study, if the results are to be disclosed and/or published, both active and negative results will be disclosed and/or published. Subject confidentiality will be maintained in accordance with privacy protections.

14. Reference

1. WHO. Recommendations to assure the quality, safety and efficacy of recombinant human papillomavirus virus-like particle vaccines[J].2006.
2. Leroux-Roels, I.; Leroux-Roels, G.; Clement, F.; Vandepapeliere, P.; Vassilev, V.; Ledent, E.; Heineman, T.C. A phase 1/2 clinical trial evaluating safety and immunogEnicity of a varicella zoster glycoprotein e subunit vaccine candidate in

young and older adults.J. Infect. Dis.2012,206, 1280–1290.

3. Godeaux, O.; Kovac, M.; Shu, D.; Grupping, K.; Campora, L.; Douha, M.; Heineman, T.C.; Lal, H. ImmunogEnicity and safety of an adjuvanted herpes zoster subunit candidate vaccine in adults >/= 50 years of agE with a prior history of herpes zoster: A phase III, non-randomized, open-label clinical trial.Hum. Vaccines Immunother.2017,13, 1051–1058.
4. Bharucha, T.; Ming, D.; Breuer, J. A critical appraisal of ‘Shingrix’, a novel herpes zoster subunit vaccine (HZ/Su or GSK1437173A) for varicella zoster virus.Hum. Vaccines Immunother.2017,13, 1789–1797）.
5. Dai X, Jayapal M, Tay HK,Reghunathan R, et al. Differential signal transduction, membrane trafficking, and immune effector functions mediated by FcγRI versus FcγRIIa. Blood. 2009,114,318-27.
