# Supplementary material for: Immunogenicity and safety of a recombinant gE-Fc fusion protein subunit vaccine for herpes zoster in adults ≥50 years of age: a randomised, active-controlled, non-inferiority trial
Source: Nat Commun. 2025 Aug 15;16:7590. doi: 10.1038/s41467-025-62800-z (PMC12356908; doi:10.1038/s41467-025-62800-z)
Supplement: Supplementary file 3 — Reporting Summary [file 41467_2025_62800_MOESM3_ESM.pdf]

nature portfolio

Corresponding author(s): DAPR NCOMMS-25-25257A

Last updated by author(s): Jul 12, 2025

Reporting Summary

Nature Portfolio wishes to improve the reproducibility of the work that we publish. This form provides structure for consistency and transparency in reporting. For further information on Nature Portfolio policies, see our Editorial Policies and the Editorial Policy Checklist.

Statistics

For all statistical analyses, confirm that the following items are present in the figure legend, table legend, main text, or Methods section.

n/a	Confirmed	
☐	☒	The exact sample size (*n*) for each experimental group/condition, given as a discrete number and unit of measurement
☐	☒	A statement on whether measurements were taken from distinct samples or whether the same sample was measured repeatedly
☐	☒	The statistical test(s) used AND whether they are one- or two-sided<br>*Only common tests should be described solely by name; describe more complex techniques in the Methods section.*
☒	☐	A description of all covariates tested
☐	☒	A description of any assumptions or corrections, such as tests of normality and adjustment for multiple comparisons
☐	☒	A full description of the statistical parameters including central tendency (e.g. means) or other basic estimates (e.g. regression coefficient) AND variation (e.g. standard deviation) or associated estimates of uncertainty (e.g. confidence intervals)
☐	☒	For null hypothesis testing, the test statistic (e.g. *F*, *t*, *r*) with confidence intervals, effect sizes, degrees of freedom and *P* value noted<br>*Give P values as exact values whenever suitable.*
☒	☐	For Bayesian analysis, information on the choice of priors and Markov chain Monte Carlo settings
☒	☐	For hierarchical and complex designs, identification of the appropriate level for tests and full reporting of outcomes
☒	☐	Estimates of effect sizes (e.g. Cohen's *d*, Pearson's *r*), indicating how they were calculated

Our web collection on statistics for biologists contains articles on many of the points above.

Software and code

Policy information about availability of computer code

Data collection	Clinflash EDC (Tigermed, China)
Data analysis	Statistical analyses were conducted using SAS software (version 9.4) and R software (version 4.3.3). Flow cytometry data were analyzed with FlowJo X 10.0.7 R2 (Tree Star)

For manuscripts utilizing custom algorithms or software that are central to the research but not yet described in published literature, software must be made available to editors and reviewers. We strongly encourage code deposition in a community repository (e.g. GitHub). See the Nature Portfolio guidelines for submitting code & software for further information.

Data

Policy information about availability of data

All manuscripts must include a data availability statement. This statement should provide the following information, where applicable:

- Accession codes, unique identifiers, or web links for publicly available datasets
- A description of any restrictions on data availability
- For clinical datasets or third party data, please ensure that the statement adheres to our policy

All data supporting the finding of this study are available in the supplementary appendix. Researchers who provide a scientifically sound proposal are allowed access to the de-identified individual participant data. Individual participant data can be obtained with a request to the corresponding authors.

Research involving human participants, their data, or biological material

Policy information about studies with human participants or human data. See also policy information about sex, gender (identity/presentation), and sexual orientation and race, ethnicity and racism.

Reporting on sex and gender	Our study was designed to include participants of both sexes, which were determined based on their identify cards. Disaggregated sex data was collected at enrollment, and consent has been obtained for sharing of individual-level data before enrollment. We collected information on the sex of each participant, and 180 (60.0%) of 300 participants were female. We used generalized linear regression models to analysis the effect of sex on gE-specific IgG antibodies and CMI responses.
Reporting on race, ethnicity, or other socially relevant groupings	The ethnicity of each participant was determined and collected in this study through each participant's identity cards. Since the percentage of Han Chinese participants was 98.7%, ethnicity was not corrected in the analysis.
Population characteristics	The mean age was approximately 60·2 years in both groups, with 152 (50·7%) participants aged 50-59 years, 114 (38·0%) participants aged 60-69 years and 34 (11·3%) participants aged 70 years and older. 180 (60·0%) of 300 participants were female, and 296 (98·7%) of 300 participants identified as Han Chinese.
Recruitment	Recruitment notices were issued to volunteers who met the enrollment criteria. The informed consent was explained to the volunteers in detail. Under the condition of voluntary participation, the volunteers and the study doctors sign the informed consent. Participants who are normal in physical examination and screened qualified as per other inclusion/exclusion criteria will be enrolled and given Research Number based on enrollment order. There were not any bias that might impact the results.
Ethics oversight	The protocol and informed consent were approved by the institutional review board of the Jiangsu Provincial Center of Disease Control and Prevention.

Note that full information on the approval of the study protocol must also be provided in the manuscript.

Field-specific reporting

Please select the one below that is the best fit for your research. If you are not sure, read the appropriate sections before making your selection.

☒ Life sciences ☐ Behavioural & social sciences ☐ Ecological, evolutionary & environmental sciences

For a reference copy of the document with all sections, see nature.com/documents/nr-reporting-summary-flat.pdf

Life sciences study design

All studies must disclose on these points even when the disclosure is negative.

Sample size	The phase 1 trial of LZ901 vaccine conducted in China, estimated that 85% of participants would have CD42+ T-cell responses and 55% would have CD82+ T-cell responses after LZ901 vaccination, compared to 75% and 45% for HZ/su participants. Assuming LZ901's T-cell responses are non-inferior to HZ/su, 150 participants per group are needed to achieve 90% power to show the lower limit of the 95% CI for the proportion difference is greater than -10%, with a one-sided α of 0.025, accounting for a 10% dropout rate.
Data exclusions	A total of 357 participants underwent eligibility screening. Among them, 55 were excluded due to failure to meet inclusion criteria, and 1 withdrew voluntarily. Of the 301 participants successfully enrolled, 1 withdrew prior to receiving the first vaccine dose; 9 withdrew before the second vaccine dose; and 6 withdrew 30 days after the second dose. The primary immunogenicity analysis was performed in the per-protocol cohort consisted of all participants who met all the eligibility criteria, completed two-dose vaccination, and had pre- and post-vaccination immunogenicity data available.
Replication	No replication had been performed in this study due to the study design (a randomised, active-controlled, non-inferiority trial). We have the protocol with the manuscript to ensure the reproducibility of this study. All experiments were performed using validated assays.
Randomization	We used an interactive web-based response-randomisation system for randomisation. Eligible participants were randomly assigned in a 1:1 ratio to either the experimental vaccine LZ901 group or active control vaccine HZ/su group. Randomisation lists were generated by an independent statistician using SAS (version 9.4), with a block size of 4.
Blinding	Unblinded staff were designated for preparing vaccines out of sight of participants and other investigators, concealing the syringes with a label of randomisation number, and administering vaccinations. Unblinded staff were aware of the treatment allocation, but were not allowed to be involved in any other trial procedures or to reveal this information to any participants or other investigators. We maintained the blinding for the safety evaluation of the study from day 0 until 30 days following the first dose, and then, unblinded the group allocations and administrated the second dose. Therefore, both the participants and investigators including safety assessors were blinded for the adverse event (AE) assessments of the first dose, but were unblinded for that of the second dose. The laboratory personnel remained blinded throughout the trial. All samples were labeled with anonymized identifiers (participant ID + timepoint) and analyzed by an independent central laboratory with no access to randomization data or clinical records that could reveal group assignment.

Reporting for specific materials, systems and methods

We require information from authors about some types of materials, experimental systems and methods used in many studies. Here, indicate whether each material, system or method listed is relevant to your study. If you are not sure if a list item applies to your research, read the appropriate section before selecting a response.

Materials & experimental systems

n/a	Involved in the study
☐	☒ Antibodies
☒	☐ Eukaryotic cell lines
☒	☐ Palaeontology and archaeology
☒	☐ Animals and other organisms
☐	☒ Clinical data
☒	☐ Dual use research of concern
☒	☐ Plants

Methods

n/a	Involved in the study
☒	☐ ChIP-seq
☐	☒ Flow cytometry
☒	☐ MRI-based neuroimaging

Antibodies

Antibodies used	Multiparametricflowcytometrypanelwasperformedusingabatteryofantibodies: PE Mouse Anti-Human CD3/UCHT1/2238757;FITC Mouse Anti-Human CD4/RPA-T4/3152878;APC-Cy™7 Mouse Anti-Human CD8/TRAP1/3360086;BV650 Mouse Anti-Human IFN-γ/4S.B3/3278390;PE-Cy™7 Rat Anti-Human IL-2/MQ1-17H12/3306154;BV421 Mouse Anti-Human TNF/MAb11/3018685;BB700 Mouse Anti-Human CD154/TRAP1/3360085;Human BD Fc Block™/Fc1/3075820 (all from BD Biosciences)
Validation	All antibodies were commercially available. See the corresponding manufacturer datasheets on webpages for reference and validation.

Clinical data

Policy information about clinical studies

All manuscripts should comply with the ICMJEguidelines for publication of clinical research and a completedCONSORT checklist must be included with all submissions.

Clinical trial registration	The study was registered with Chinese Clinical Trial Registry, with registration number ChiCTR2300079076
Study protocol	The study protocol is available in the Supplementary Information file.
Data collection	This study was conducted in Wuxi, Jiangsu province, China. Blood samples were collected for immunogenicity assessments at baseline before vaccination and day 30 after the second dose. Peripheral blood mononuclear cells (PBMCs) were collected from all participants to assess T-cell mediated immune responses using intracellular cytokine staining (ICS) flow cytometry. Serum anti-gE antibody concentrations were measured by China National Institutes for Food and Drug Control (NIFDC) with an in-house enzyme-linked immunosorbent assay (ELISA).
Outcomes	The primary outcomes was the proportion of participants with simultaneous positive responses to two or more cytokines (IFN-γ, IL-2, TNF-α, or CD40L) 30 days after the second dose (referred to as gE-specific CD4+/CD82+ T-cell responses). For each cytokine, a positive responder was defined as a 2-fold increase in cytokine-secreting T cells post-vaccination compared to pre-vaccination levels.<br>The secondary immunogenicity outcomes were the frequencies of cytokine-producing CD4+/CD8+ T cells per 100000 PBMCs, geometric mean concentrations (GMCs), geometric mean fold increase (GMFI) and seroconversion of gE-specific IgG antibodies day 30 after the second dose. Seropositivity of gE-specific IgG antibodies was defined as anti-gE antibodies concentration ≥ 100 milli-International Units (mIU)/mL. While, the seroconversion was defined as a ≥4-fold increase in anti-gE antibodies concentration at day 30 compared to that at pre-vaccination. Safety outcomes included the incidence of adverse reactions within 30 days after each dose and serious adverse events (SAEs) over 6 months.

Plants

Seed stocks	*Report on the source of all seed stocks or other plant material used. If applicable, state the seed stock centre and catalogue number. If plant specimens were collected from the field, describe the collection location, date and sampling procedures.*
Novel plant genotypes	*Describe the methods by which all novel plant genotypes were produced. This includes those generated by transgenic approaches, gene editing, chemical/radiation-based mutagenesis and hybridization. For transgenic lines, describe the transformation method, the number of independent lines analyzed and the generation upon which experiments were performed. For gene-edited lines, describe the editor used, the endogenous sequence targeted for editing, the targeting guide RNA sequence (if applicable) and how the editor was applied.*
Authentication	*Describe any authentication procedures for each seed stock used or novel genotype generated. Describe any experiments used to assess the effect of a mutation and, where applicable, how potential secondary effects (e.g. second site T-DNA insertions, mosiacism, off-target gene editing) were examined.*

Flow Cytometry

Plots

Confirm that:

- [x] The axis labels state the marker and fluorochrome used (e.g. CD4-FITC).
- [x] The axis scales are clearly visible. Include numbers along axes only for bottom left plot of group (a 'group' is an analysis of identical markers).
- [x] All plots are contour plots with outliers or pseudocolor plots.
- [x] A numerical value for number of cells or percentage (with statistics) is provided.

Methodology

Sample preparation	Blood samples were collected for immunogenicity assessments at baseline before vaccination and day 30 after the second dose. Peripheral blood mononuclear cells (PBMCs) were collected from all participants to assess T-cell mediated immune responses using intracellular cytokine staining (ICS) flow cytometry. Peripheral Mononuclear cells (PBMC), serum and whole blood were frozen in Wuxi CDC. Cryopreserved PBMC were thawed and rested, fixed and stained in China National Institutes for Food and Drug Control (NIFDC) according to the demands on each experiment.
Instrument	BD FACSCanto™ Flow Cytometer, BD Biosciences, San Jose, CA, USA
Software	FlowJo X 10.0.7 R2 (Tree Star)
Cell population abundance	1 million cells were stained and collected on cytometer.
Gating strategy	We have provided gating strategies for flow cytometry in the supplementary information.

- [x] Tick this box to confirm that a figure exemplifying the gating strategy is provided in the Supplementary Information.
